# Discovery of 3,6-disubstituted pyridazines as a novel class of anticancer agents targeting cyclin-dependent kinase 2: synthesis, biological evaluation and in silico insights

**DOI:** 10.1080/14756366.2020.1806259

**Published:** 2020-08-11

**Authors:** Ahmed Sabt, Wagdy M. Eldehna, Tarfah Al-Warhi, Ohoud J. Alotaibi, Mahmoud M. Elaasser, Howayda Suliman, Hatem A. Abdel-Aziz

**Affiliations:** a Chemistry of Natural Compounds Department, National Research Centre, Dokki, Egypt; b Department of Pharmaceutical Chemistry, Faculty of Pharmacy, Kafrelsheikh University, Kafrelsheikh, Egypt; c Department of Chemistry, College of Science, Princess Nourah bint Abdulrahman University, Riyadh, Saudi Arabia; d The Regional Center for Mycology and Biotechnology, Al-Azhar University, Cairo, Egypt; e Department of Medical Biochemistry, Faculty of Medicine, Alexandria University, Alexandria, Egypt; f Department of Applied Organic Chemistry, National Research Center, Giza, Egypt

**Keywords:** Breast cancer, aminopyridazines, CDK2 inhibitors, apoptosis inducers, molecular docking, cell cycle arrest

## Abstract

Human health in the current medical era is facing numerous challenges, especially cancer. So, the therapeutic arsenal for cancer should be unremittingly enriched with novel small molecules that selectively target tumour cells with minimal toxicity towards normal cells. In this context, herein a new series of 3,6-disubstituted pyridazines **11a–r** has been synthesised and evaluated for *in vitro* anticancer activity. They possessed good anti-proliferative action towards human breast cancer T-47D (IC_50_ range: 0.43 ± 0.01 − 35.9 ± 1.18 µM) and MDA-MB-231 (IC_50_ range: 0.99 ± 0.03 − 34.59 ± 1.13 µM) cell lines, whereas they displayed weak activity against the tested ovarian cancer cell line SKOV-3. Among the studied compounds, the methyltetrahydropyran-bearing pyridazine **11m** emerged as the unique submicromolar growth inhibitor herein reported towards both T-47D (IC_50_ = 0.43 ± 0.01 µM) and MDA-MB-231 (IC_50_ = 0.99 ± 0.03 µM) cell lines. In addition, the biological results indicated that pyridazines **11l** and **11m** exerted an efficient alteration within the cell cycle progression as well as induction of apoptosis in both T-47D and MDA-MB-231 cells. Moreover, pyridazines **11l** and **11m** displayed good mean tumour S. I. values of 13.7 and 16.1 upon assessment of their cytotoxicity towards non-tumorigenic breast MCF-10A cells. Furthermore, an *in silico* study proposed CDK2 as a probable enzymatic target for pyridazines **11**, and explored their binding interactions within the vicinity of CDK2 binding site. Subsequently, pyridazines **11e**, **11h**, **11l**, and **11m** were selected to be evaluated for their ability to inhibit CDK2, where they exerted good inhibitory activity (IC_50_ = 151, 43.8, 55.6 and 20.1 nM, respectively). Finally, the *in silico* study implied that target pyridazines **11** exhibited not only an efficient anticancer activity but also an acceptable ADME, physicochemical and druglikeness properties, specifically pyridazines **11l** and **11m**. Overall the obtained results from this study quite sustained our strategy and gave us a robust opportunity for further development and optimisation of 3,6-disubstituted pyridazine scaffold to enrich therapeutic arsenal with efficient and safe anticancer CDK inhibitors.

## 1. Introduction

Cancer is a very complex disease, which affects diverse systems and organs within the body. Cancer develops because of the abnormal and uncontrolled cells division, initiated as a result of chemicals, viruses, smoking, or diet. Complications of cancer disease lead to death if left without treatment^1^. As a serious health problem, mortality due to cancer is expected to surpass that attributable to cardiovascular disorders in a short time. Around seven million cancer-related cases die per year, whereas about more than 26 million new cancer cases and 17 million cancer-related deaths are estimated to be reached per year by 2030^2^. Accordingly, the therapeutic arsenal for cancer treatment should be urgently enriched with novel small molecules directed towards a certain signalling factor that could be implicated in tumour growth and/or tumorigenesis.

Cyclin-dependent kinases (CDKs) are a family of comparatively small proteins, from 34 to 40 kDa, which is classified as serine/threonine protein kinases^3^. CDKs have essential roles in the cell cycle regulation as well as in apoptosis, transcription and differentiation through binding to a regulatory protein known as “cyclin”; only the CDK-cyclin complexes are active kinases^4^^,^^5^.

**Figure 1.**
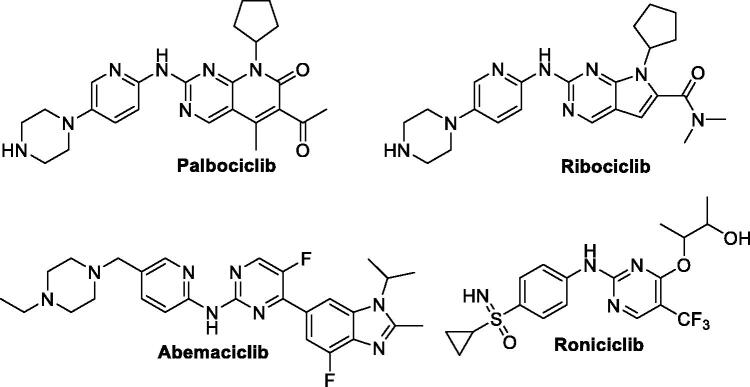
Chemical structure of the clinically used (palbociclib, abemaciclib and ribociclib) or currently in clinical trials (roniciclib) CDK inhibitors.

It is well established that excessive production of CDKs, such as CDK1, CDK2, CDK4 and CDK6, or cyclins may result in a disruption of the normal regulation controls and eventually leads to cancer. For this reason, therapeutic approach based on inhibition of CDKs represents an auspicious opportunity and promising strategy for drug discovery and development of novel efficient and targeted chemical entities that can fight different types of human malignancies^6^. To date, three small molecule CDK inhibitors (palbociclib, abemaciclib and ribociclib) have been approved for clinical use, Figure 1^7^, whereas there are several CDK inhibitors in the clinical trials; such as roniciclib (BAY 1000394, Figure 1)^8^. However, the side effects and resistance to these inhibitors somehow limits their therapeutic value and discloses the necessity of development of novel CDK inhibitors^9^.

Over the last two decades, non-fused pyridazine nucleus has emerged as an important class of heterocycles that represents a promising and privileged scaffold in medicinal chemistry^10^. Literature survey revealed that plenty of pyridazine-based small molecules have been extensively investigated for a broad array of significant biological actions, such as anti-inflammatory^11^^,^^12^, anti-hypertensive^13^, anti-diabetic^14^, anti-obesity^15^, neuroprotective^16^, anti-Alzheimer’s^17^, anti-tubercular^18^, anti-HIV^19^ and anticancer^20^ activities.

**Figure 2.**
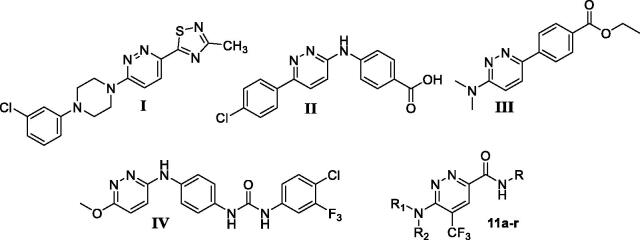
Chemical structure for some 3,6‐disubstituted pyridazine derivatives reported as efficient anticancer small molecules.

While several studies and research work have explored different anticancer activities for diverse fused pyridazine derivatives, little attention has been paid to investigate the anticancer activity for the non-fused 3,6-disubstituted pyridazine derivatives over the last two decades^21–30^. For example; compound **I** (Figure 2) displayed potent *in vitro* and *in vivo* antitumor and anti-angiogenesis activities^21^, compound **II** (Figure 2) efficiently inhibited the cell proliferation in a panel of breast, colon, prostate and liver human tumours^28^. In addition, compound **III** (Figure 2) elicited excellent cytotoxic activity towards human colon cancer HT-29 cell line^22^, whereas compound **IV** (Figure 2) emerged as promising VEGFR-2 inhibitor with IC_50_ in the nanomolar range^29^.

Inspired by the aforementioned findings and as part of our research work on the discovery of efficient anticancer candidates, herein we report in details the synthesis and anticancer activities assessment for a new series of non-fused 3,6-disubstituted pyridazine derivatives **11a–r** (Figure 2). In this context, the anticancer actions for the 3,6-disubstituted pyridazine derivatives here reported; **11a–r** will be evaluated against three human cancer cell lines, namely, T-47D and MDA-MB-231 (breast cancers), and SKOV-3 (ovarian cancer) cell lines utilising the protocol of SRB assay. Thereafter, the most potent anti-proliferative pyridazines will be selected to explore their plausible mechanism of action through cell cycle analysis as well as Anx V-FITC apoptosis assay in both breast cancer (T-47D and MDA-MB-231) cell lines. Finally, an *in silico* study suggested CDK2 as a probable enzymatic target for the herein reported 3,6-disubstituted pyridazines **11a–r** and explored their binding interactions within the vicinity of CDK2 binding site, thereafter, target pyridazines will be explored for their potential inhibitory activity against CDK2.

## 2. Results and discussion

### 2.1. Chemistry

**Scheme 1.**
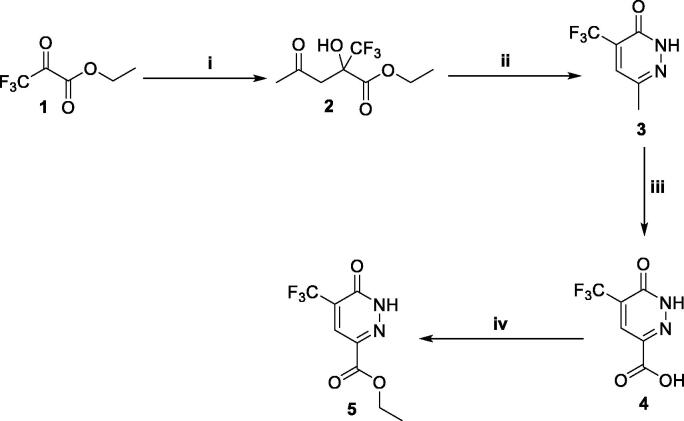
Preparation of ester **5**; reagents and conditions: (**i**) Acetone, DMF, L-proline, r.t., 48 h; (**ii**) NH_2_NH_2_, AcOH, reflux, 6 h; (**iii**) K_2_Cr_2_O_7_, Conc. H_2_SO_4_, 0 °C to r.t. overnight; (**iv**) EtOH, H_2_SO_4_, reflux, 4 h.

**Scheme 2.**
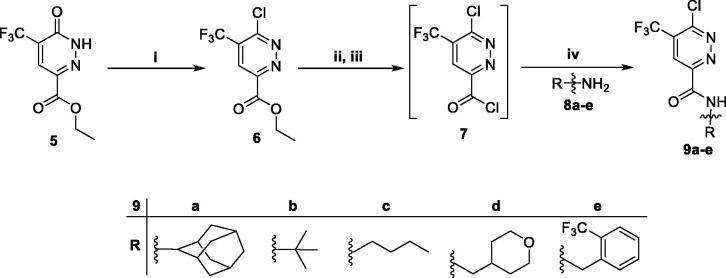
Preparation of intermediateds **9a–e**; reagents and conditions: (**i**) POCl_3_, 100 °C, 5 h; (**ii**) LiOH, THF, H_2_O, r.t., 1 h; (**iii**) SOCl_2_, DMF (catalytic), 1,2-dichloroethane, reflux, 3 h; (**iv**) Methylene chloride, Et_3_N, r.t., 4 h.

**Scheme 3.**
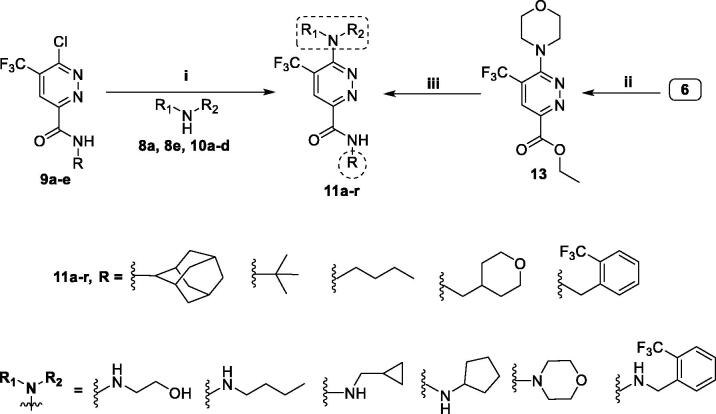
Preparation of target pyridazines **11a–r**; reagents and conditions: (**i**) 1,4-Dioxane, Hünig’s base, reflux, 6 h; (**ii**) Morpholine, 1,4-dioxane, Hünig’s base, reflux, 12 h; (**iii**) Primary amines **8a–e**, EtOH, piperdine, reflux, 6 h.

The target 5-(trifluoromethyl)pyridazine-3-carboxamide derivatives (**11a–r**) were prepared through several synthetic steps (Schemes 1–3) starting from the commercially available ethyl 3,3,3-trifluoropyruvate. With respect to Scheme 1, ethyl trifluoropyruvate (**1**) reacted with acetone in the existence of L-proline and DMF following a reported method^31^ to yield ethyl 2-hydroxy-4-oxo-2-(trifluoromethyl)pentanoate (**2**), the later compound was converted into 6-methyl-4-(trifluoromethyl)pyridazin-3(2H)-one (**3**) upon reaction with hydrazine hydrate in the existence of acetic acid^32^. Compound (**3**) was then subjected to oxidation process using potassium chromate and sulphuric acid at room temperature to give 6-oxo-5-(trifluoromethyl)-1,6-dihydropyridazine-3-carboxylic acid (**4**). Fischer esterification was then carried out for compound (**4**) through reflux with ethanol in the presence of sulphuric acid (catalytic amount) in order to afford the corresponding ester derivative (**5**), Scheme 1.

Chlorination of 6-oxo-1,6-dihydropyridazine derivative (**5**) was performed *via* its reflux with excess phosphorous oxychloride to furnish ethyl 6-chloro-5-(trifluoromethyl)pyridazine-3-carboxylate (**6**), which subsequently hydrolysed *via* treatment with the alkali lithium hydroxide in presence of THF/H_2_O (4/1) then chlorinated with thionyl chloride to afford the crude acid chloride derivative (**7**). Stirring of acid chloride (**7**) with the different primary amines **8a–e** in dichloromethane at r.t. and in the existence of Et_3_N led to formation of the key amide intermediates **9a–e** (Scheme 2).

The target pyridazine derivatives (**11a–r**) were prepared *via* two synthetic routes. The first route utilised the key amide intermediates **9a–e**, which reacted with the amines **8a**, **8e** and **10a–d** in refluxing 1,4-dioxane in the presence of Hünig’s base in order to furnish target pyridazines **11a–r** (Scheme 3). This synthetic pathway proved successful to prepare target pyridazines **11a–r** with low to good yields; 36–79%. In the second route, ethyl 6-chloro-5-(trifluoromethyl)pyridazine-3-carboxylate (**6**) was subjected to a nucleophilic substitution with morpholine in boiling 1,4-dioxane in the existence of Hünig’s base to furnish ethyl 6-morpholino-5-(trifluoromethyl)pyridazine-3-carboxylate (**13**), which subsequently underwent direct amidation that accomplished *via* reaction with primary amines **8a–e** in absolute ethyl alcohol in the existence of piperidine to yield the corresponding target pyridazines **11d**, **11g**, **11i**, **11m** and **11r**, respectively, with overall 29–54% yield for the two steps; nucleophilic substitution and direct amidation (Scheme 3)^33^.

It is worth emphasising that pyridazines **11d**, **11g**, **11i**, **11m** and **11r** were also prepared utilising the first route, from the key amide intermediates **9a–e**, with higher yields (50–73%) than those afforded from the second route (29–54%), suggesting first route as more advantageous to synthesise final target pyridazines.

### 2.2. Biological evaluation

#### 2.2.1. *In vitro* anti-proliferative activities

The newly herein reported pyridazines **11a–r** were assessed for their anti-proliferative potential towards three human cancer cell lines, namely, T-47D (breast cancer), MDA-MB-231 (breast cancer) and SKOV-3 (ovarian cancer) cell lines by using the protocol of the SRB assay^34^. MDA-MB-231 cell line is classified as a triple-negative/basal-like cell line, which is hormone (estrogen and progesterone)-receptor negative and HER2 negative, whereas T-47D is considered as hormone-receptor positive and HER2 negative breast cancer cell line. The IC_50_ values for target pyridazines **11a–r** were determined, and were represented in Table 1.

**Table 1.** Anti-proliferative activities for the newly prepared pyridazines (**11a–r**) towards T-47D, MDA-MB-231 and SKOV-3 cell lines.




^a^IC_50_s are displayed as mean ± SD for three separate experiments.

^b^NA: Pyridazines with IC_50_ value more than 100 µM.

The obtained results disclosed that all the screened pyridazines possessed excellent to moderate anticancer action against the two tested breast cancer (T-47D and MDA-MB-231) cell lines, whereas they displayed weak to non-significant cytotoxic impact against the tested ovarian cancer (SKOV-3) cell line, Table 1. Nonetheless, it was noted that the screened pyridazines **11a–r** exhibited differential activities against the two tested human breast cancer cell lines; 8 out of 18 compounds were more active against T-47D than MDA-MB-231, whereas 10 out of 18 compounds were more active against MDA-MB-231 than T-47D as indicated in Table 1. In details, T-47D cells were more sensitive (IC_50_ range 0.44 ± 0.01 − 11.44 ± 0.37 µM) to the influence of all 2-adamantyl-bearing pyridazines **11a–e** (except **11d**), *tert*-butyl-bearing pyridazines **11f–h** (except **11 g**) and butyl-bearing pyridazine (**11i**) than MDA-MB-231 cells (IC_50_ range 2.18 ± 0.07 − 34.59 ± 1.13 µM), Table 1. On the contrary, the methyltetrahydropyran-bearing pyridazines **11j–l** and 2-(trifluoromethyl)benzyl-bearing pyridazines **11n–r** displayed better cytotoxic activity against MDA-MB-231 (IC_50_ range 1.30 ± 0.04 − 19.71 ± 0.64 µM) than T-47D cell line (IC_50_ range 1.57 ± 0.05 − 35.9 ± 1.18 µM).

Concerning the anti-proliferative activity of target pyridazines **11a–r** against breast cancer T-47D cell line, the results indicated that the growth of the T-47D cells was effectively inhibited by all pyridazines reported here with IC_50_s spanned in the range 0.43 ± 0.01 − 15.76 ± 0.51 µM, except for **11j** and **11r** which weakly inhibited the T-47D growth (IC_50_ = 35.9 ± 1.18 and 24.80 ± 0.81 µM, respectively). Outstandingly, two pyridazines displayed submicromolar potency against T-47D cells were identified, that were the butyl-bearing pyridazine (**11i**) and methyltetrahydropyran-bearing pyridazine (**11m**), whose IC_50_s of 0.44 ± 0.01 and 0.43 ± 0.01 µM represent the best growth inhibition here reported for T-47D cells (Table 1). Interestingly, both pyridazines **11i** and **11m** are decorated with morpholine moiety at C-6 position. Moreover, pyridazines **11b**, **11e**, **11f**, **11h** and **11l** showed excellent cytotoxic activity against pyridazines **11m** T-47D cell line with IC_50_ values equal 1.37 ± 0.04, 2.62 ± 0.08, 1.94 ± 0.06, 1.60 ± 0.05 and 1.57 ± 0.05 µM, respectively.

With respect to the anticancer activity against MDA-MB-231 cells, the obtained growth inhibitory data (Table 1) ascribed to most newly synthesised pyridazines **11** good efficacy in inhibiting the growth of MDA-MB-231 cells, with IC_50_s ranging between 0.99 ± 0.03 and 19.71 ± 0.64 µM. Uniquely, compound **11a** induced weak growth inhibition towards MDA-MB-231 cell line with IC_50_ equals 34.59 ± 1.13 µM (Table 1). It is worth stressing that methyltetrahydropyran-bearing pyridazine (**11m**) superiorly displayed submicromolar potency towards MDA-MB-231 cell line (IC_50_ = 0.99 ± 0.03 µM), and thus **11m** emerged as the unique submicromolar growth inhibitor herein reported towards both breast cancer T-47D and MDA-MB-231 cell lines. In addition, pyridazines **11d**, **11h**, **11l** and **11n** elicited excellent growth inhibitory action towards MDA-MB-231 cell lines with IC_50_ values of 2.18 ± 0.07, 2.44 ± 0.08, 1.30 ± 0.04 and 2.94 ± 0.09 µM, respectively (Table 1).

On the other hand, the cytotoxic action for pyridazines **11l** and **11m**, the most potent anti-proliferative agents with dual growth inhibitory activities towards T-47D and MDA-MB-231, against non-tumorigenic human breast (MCF-10A) cells has been assessed by the use of the MTT assay in order to explore the selectivity and safety for target pyridazines reported here towards the non-tumorigenic normal cells. The obtained IC_50_ values were displayed in Table 2, in addition to the calculated mean tumour selectivity index (S. I.); IC_50_ for MCF-10A/ IC_50_ average for (T-47D and MDA-MB-231). As results indicated, examined pyridazines **11l** and **11m** displayed good mean tumour S. I. values of 13.7 and 16.1, respectively, which discloses good safety profile for target pyridazines as antitumor agents.

**Table 2.** Cytotoxic impact for pyridazines **11l** and **11m** against non-tumorigenic MCF-10A human breast cell line, as well as mean tumour selectivity index (S. I.) (MCF-10A/T-47D and MDA-MB-231).

Comp.	IC_50_ (µM)			Mean tumour selectivity
	MCF-10A	T-47D	MDA-MB-231	
**11l**	19.63 ± 0.38	1.57 ± 0.05	1.30 ± 0.04	13.7
**11m**	11.44 ± 0.22	0.43 ± 0.01	0.99 ± 0.03	16.1

#### 2.2.2. Cell cycle analysis

In this study, the efficient anti-proliferative activities of target pyridazines **11**a-r towards breast cancer cell lines promoted us to explore and get insights into the plausible cellular mechanisms for these small molecules. To this end, we decided to determine if the target pyridazines exerted their cytotoxic activity through alteration in cell cycle progression and/or induction of apoptosis in both breast cancer T-47D and MDA-MB-231 cells. As a reliable tool, the flow cytometric analysis was utilised to inspect the cell cycle distribution after treatment of T-47D and MDA-MB-231 cells with IC_50_s of pyridazines **11l** and **11m**; the most potent cytotoxic agents towards both examined breast cancer cell lines (Table 1).

**Figure 3.**
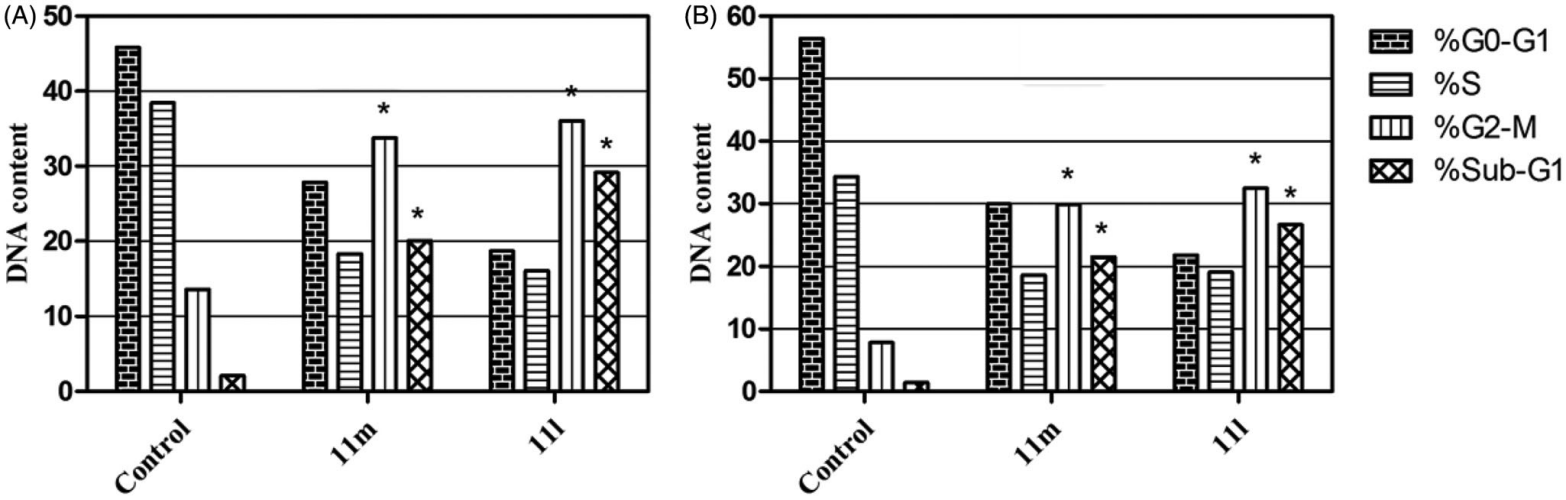
Influence of pyridazines **11l** and **11 m** on the distribution of cell cycle phases in breast cancer T-47D (**A**) and MDA-MB-231 (**B**) cells. Asterisk indicates a significant difference from control at *p* < 0.05.

As results indicated, pyridazines **11l** and **11m** were able to provoke a significant rise in the cellular population of the G2/M phase in T-47D cells by 2.7- and 2.5-fold, respectively, with concurrent significant increase in the sub-G1 phase by 13.8- and 9.5-fold, respectively, Figure 3(A). In addition, treatment of MDA-MB-231 cells by pyridazines **11l** and **11m** elicited a significant increase in the cell population within both G2/M phase (by 4.1 and 3.8-fold, respectively) and sub-G1 phase (by 18.9- and 15.2-fold, respectively) with respect to untreated control cells (Figure 3(B)). Conclusively, both alteration of the Sub-G_1_ phase as well as arrest of G_2_-M phase are featured as significant remarks for pyridazines **11l** and **11m** to trigger apoptosis in T-47D and MDA-MB-231 cancer cells.

#### 2.2.3. Annexin V-FITC apoptosis assay

**Figure 4.**
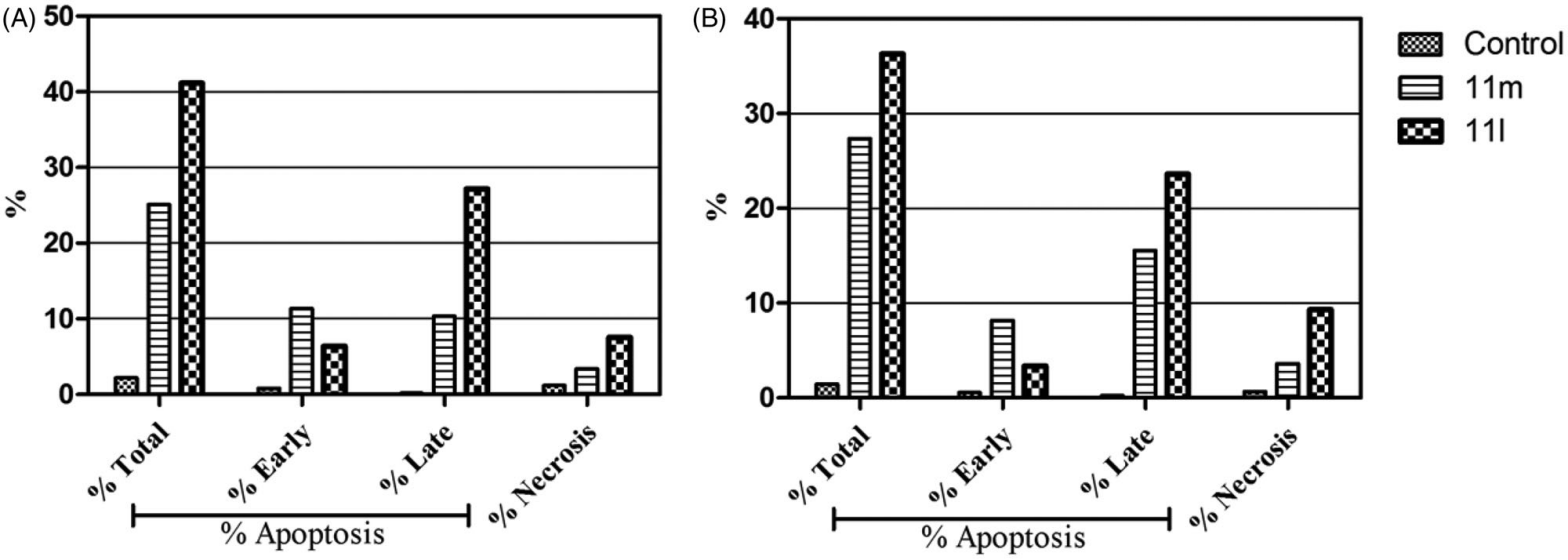
Percentage of apoptotic and necrotic cells in T-47D (**A**) and MDA-MB-231 (**B**), with respect to untreated control cells, upon treatment with pyridazines **11l** and **11m**.

**Figure 5.**
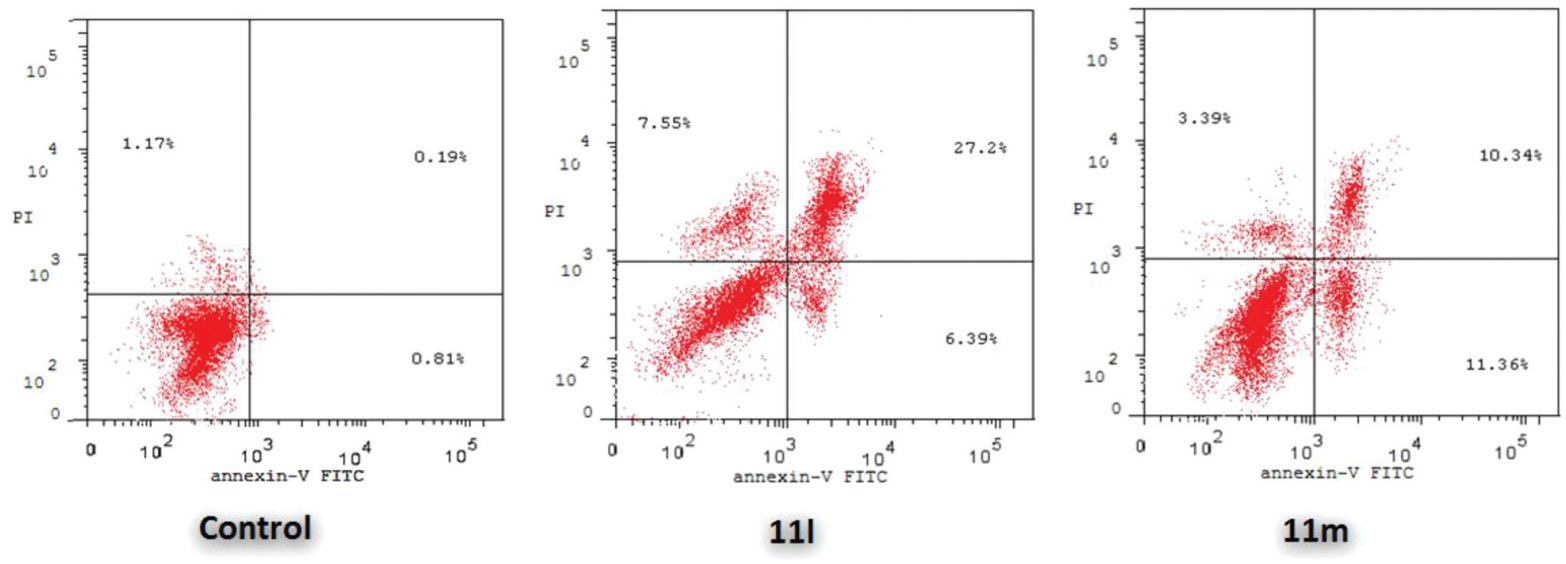
Influence of pyridazines **11l** and **11m** on the percentage of Annexin V^+^ apoptotic cells in T-47D cells.

**Figure 6.**
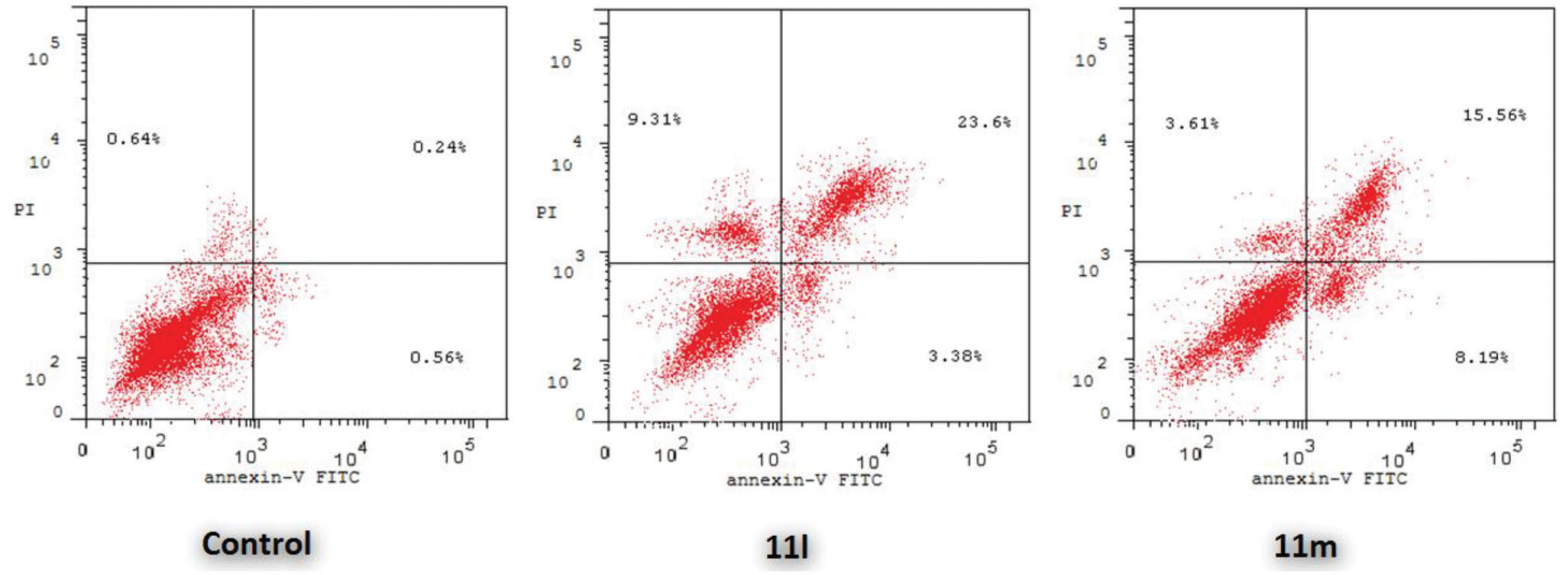
Influence of pyridazines **11l** and **11m** on the percentage of Annexin V^+^ apoptotic cells in MDA-MB-231 cells.

In this study, the apoptotic potential for pyridazines **11l** and **11m**, the most potent anti-proliferative agents herein reported, has been assessed in both breast cancer T-47D and MDA-MB-231 cell lines using Annexin V FITC/PI dual staining assay. Both T-47D and MDA-MB-231 cells were treated with pyridazines **11l** and **11m** at their IC_50_s for 48 h. The obtained results have been depicted in Figures 4–6.

The outcomes from the flow cytometric analysis revealed that T-47D cells treated with pyridazines **11l** and **11m** exhibited a significant rise in the percent of Anx V-FITC positive (Annexin V^+^) apoptotic cells within both early (from 0.81% to 6.39% and 11.36%, respectively) and late (from 0.19% to 27.2% and 10.34%, respectively) apoptotic phases (UR + LR), which involves about 33- and 21-fold increase with respect to untreated control cells (Figures 4(A) and 5).

On the other hand, the flow cytometric analysis disclosed that the apoptotic rates (Annexin V^+^) of MDA-MB-231 cells were significantly increased from 0.56% for untreated control cells to 3.38% and 8.19%, respectively, in the early apoptotic phase, as well as from 0.24% for untreated control cells to 23.60% and 15.56%, respectively, in the late apoptotic phase; after treatment of MDA-MB-231 cells with the IC_50_s of pyridazines **11l** and **11m** (Figures 4(B) and 6).

### 2.3. In silico target prediction

Swiss Target Prediction is an online tool that created in 2014 in order to predict the potential targets for any small molecule^35^. This tool can deduce the best potential macromolecular targets for the small molecules, assumed as bioactives. The predictions are based on 2D and 3D similarity combination with a library includes more than 370’000 known actives towards more than three thousands proteins from 3 different species^36^. In this study we utilised the online SwissTargetPrediction tool to explore the potential enzymatic targets for the herein reported pyridazine derivatives.

The generated prediction results, for two representative compounds **11a** and **11j**, suggested the protein kinases as the most probable targets (Supporting materials). In particular, CDK2 is listed as one of the top suggested kinases. It is interesting to note that the suggestion of CDK2 as a potential target is perfectly tuned with its known expression in breast cancer^37^, and with the obtained results from the cell cycle analysis, as the role of CDK2 in the cell cycle is well-established^3^. Accordingly, a molecular docking work was conducted in order to examine the plausible binding modes and interactions of the target pyridazines **11a–r** within the active site of CDK2.

### 2.4. Cdk2 inhibitory activity

#### 2.4.1. Molecular docking

Molecular docking is among the most utilised drug design techniques that provide insights on how ligands could bind to their targets and the efficiency of theses ligands by scoring them on basis of energy and interactions. Based on suggestion of SwissTargetPrediction tool that CDK2 kinase could be a potential target for the synthesised pyridazines, docking was used to validate this assumption and also to select the most promising analogues for a further biological screening.

Initially, the protocol of the molecular docking has been validated *via* redocking of the co-crystalized ligand Roniciclib within the vicinity of CDK2 binding site. The performed redocking procedure re-produced the original binding manner for the co-crystallized Roniciclib quite adequately implying the convenience of the utilised steps for the desired docking analysis. This was evidenced by the small RMSD of 0.53 Å between the co-crystallized Roniciclib and the docked pose (energy score (*S*) = −9.2 kcal/mol), as well as by the reproducing of all interactions accomplished by the co-crystallized Roniciclib within the CDK2 active site.

**Figure 7.**
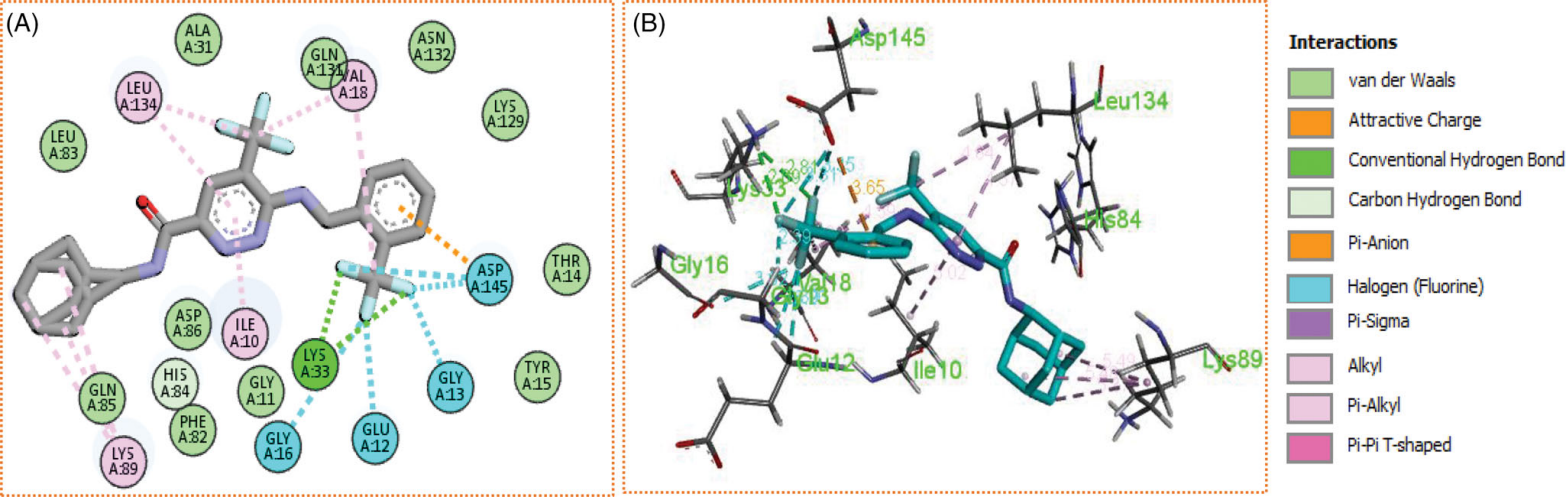
(A) 2D, and (B) 3D diagram for pyridazine **11e** demonstrating its interactions within the CDK2 active site.

**Figure 8.**
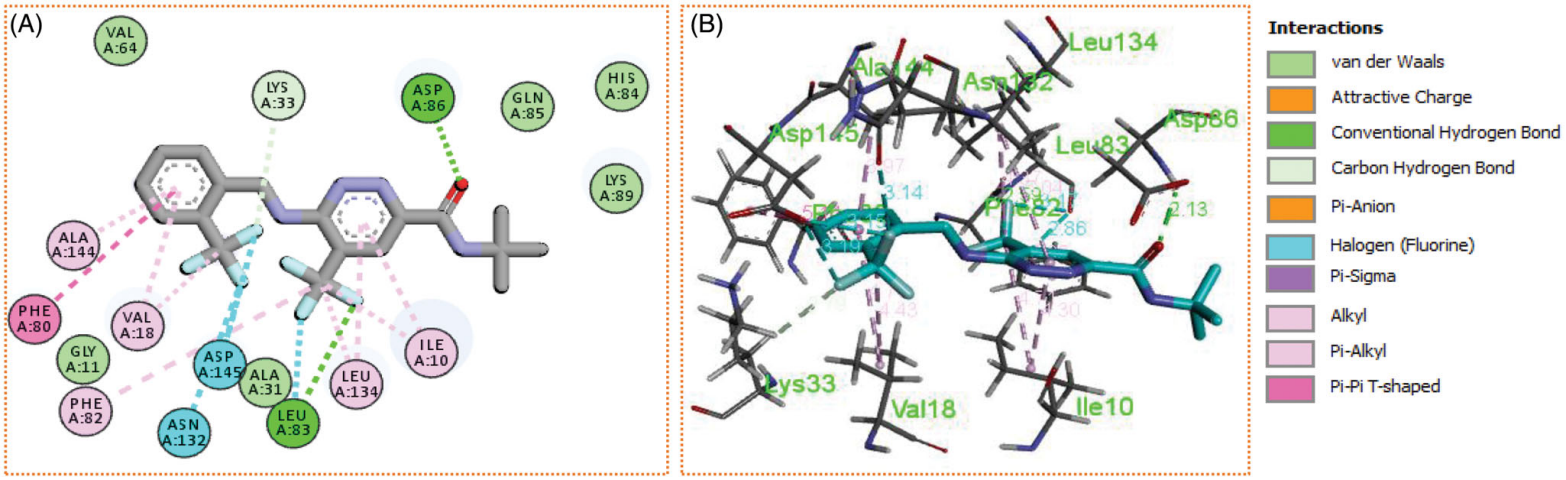
(A) 2D, and (B) 3D diagram for pyridazine **11h** demonstrating its interactions within the CDK2 active site.

**Figure 9.**
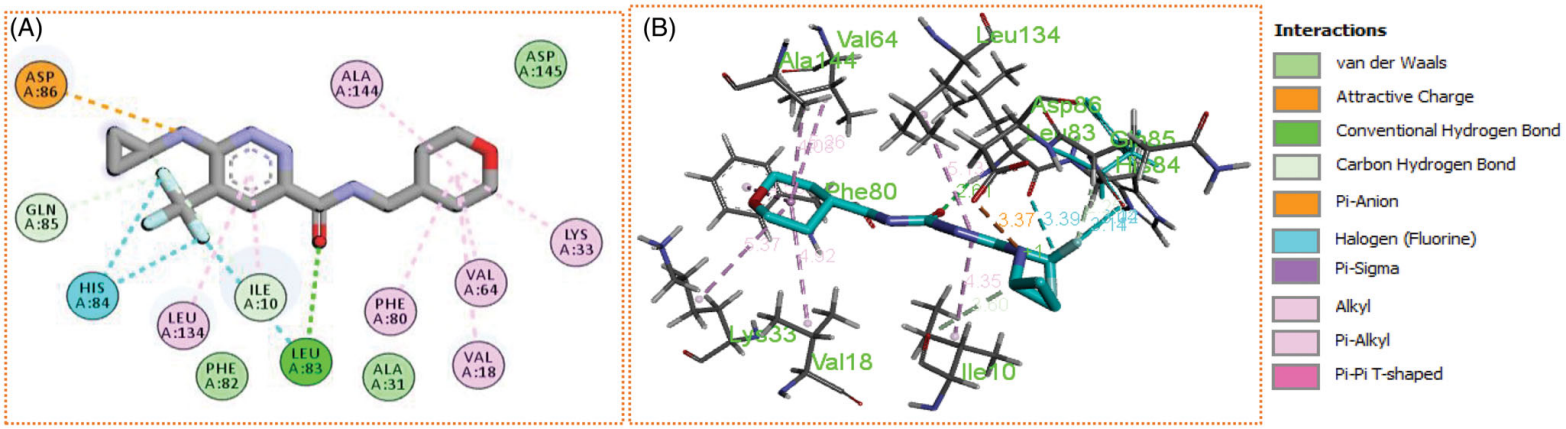
(A) 2D, and (B) 3D diagram for pyridazine **11l** demonstrating its interactions within the CDK2 active site.

**Figure 10.**
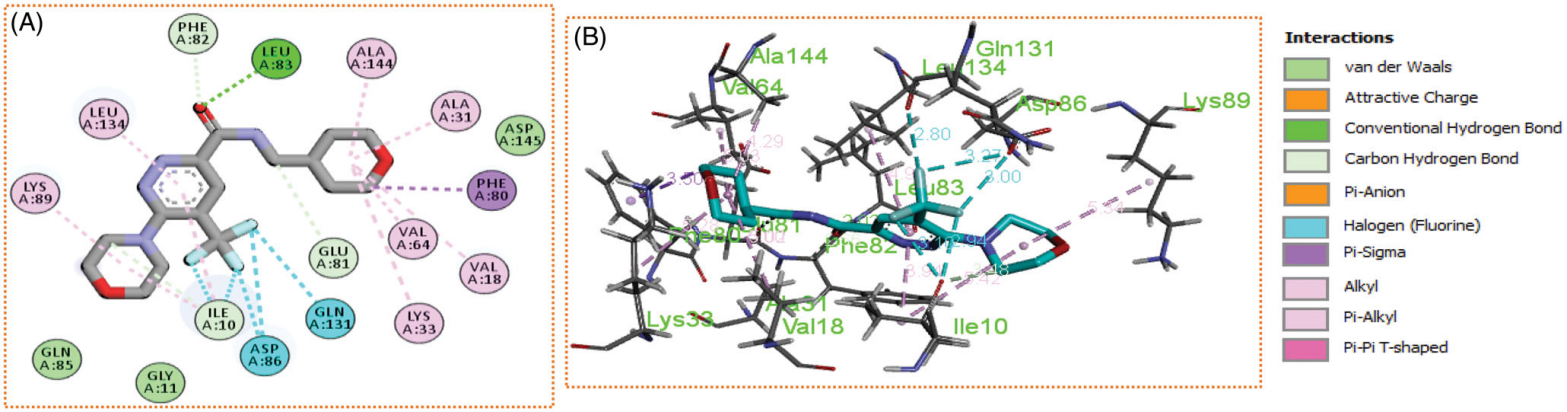
(A) 2D, and (B) 3D diagram for pyridazine **11m** demonstrating its interactions within the CDK2 active site.

Generally, all the target pyridazines **11a–r** achieved acceptable binding interactions and energy scores. In particular, compounds **11m**, **11e**, **11h** and **11l** achieved the highest scores (−10.2, −10.1, −9.5 and −9.0 Kcal\mole, respectively, Table 3). The four compounds (**11e**, **11h**, **11l**, and **11m**) have successfully bound strongly to the CDK2 active site; forming different types of interaction including hydrophobic interactions with non-polar residues (such as Ala31, Val18, Val64, Phe80, Ala144, Phe82 and Leu134), as well as hydrogen bonding with other residues (such as Asp86, Leu83 and Lys89), Figures 7–10. All the bonding interactions within CDK2 active site for pyridazines **11e**, **11h**, **11l**, and **11m** were outlined in Table 4. The obtained results from the docking study supported the assumption generated from the SwissTargetPrediction server and directed us to conduct a biological evaluation for the best energy scoring pyridazines **11e**, **11h**, **11l**, and **11m** against CDK2 enzyme.

**Table 3.** Docking energy scores (*S*) for pyridazines **11a–r** and the co-crystallized ligand Roniciclib (in in kcal/mol).

Cpd.	Energy score (*S*) Kcal\mole	Cpd.	Energy score (*S*) Kcal\mole
**11a**	−8.1	**11j**	−8.3
**11b**	−8.0	**11k**	−8.7
**11c**	−8.2	**11l**	**−9.5**
**11d**	−7.9	**11m**	**−10.2**
**11e**	**−9.0**	**11n**	−8.6
**11f**	−8.3	**11o**	−8.0
**11g**	−7.9	**11p**	−8.7
**11h**	**−10.1**	**11q**	−8.2
**11i**	−8.5	**11r**	−8.4
**Roniciclib**	−9.2		

Bold values are for pyridazines with best docking energy scores.

**Table 4.** The different bonding types and their distances (in Å) for pyridazines **11e**, **11h**, **11l**, and **11m** within the CDK2 active site.

Cpd	Bond type (Involved A. A.)	Distance	Cpd	Bond type (Involved A. A.)	Distance
**11e**	Hydrogen bond (Lys33)	2.69	**11l**	Hydrogen bond (Leu83)	2.61
	Hydrogen bond (Lys33)	2.81		Halogen interaction (Leu83)	3.39
	Halogen interaction (Asp145)	3.31		Halogen interaction (His84)	3.14
	Halogen interaction (Asp145)	3.35		Halogen interaction (His84)	3.02
	Halogen interaction (Gly13)	2.39		Pi-Alkyl (Ile10)	4.35
	Halogen interaction (Gly16)	3.62		Non-classical hydrogen bond (Ile10)	3.60
	Halogen interaction (Glu12)	3.26		Ionic bond (Asp86)	3.37
	Pi-Anion (Asp145)	3.65		Pi-Alkyl (Leu134)	5.13
	Pi-Alkyl (Leu134)	4.57		Pi-Alkyl (Phe80)	4.53
	Alkyl-Alkyl (Leu134)	4.64		Alkyl-Alkyl (Val18)	4.92
	Alkyl-Alkyl (Val18)	4.99		Alkyl-Alkyl (Val64)	5.26
	Alkyl-Alkyl (Val18)	4.59		Alkyl-Alkyl (Ala144)	4.08
	Pi-Alkyl (Ile10)	5.02		Alkyl-Alkyl (Lys33)	5.37
	Alkyl-Alkyl (Lys89)	5.37		Non-classical hydrogen bond (Gln85)	2.25
	Alkyl-Alkyl (Lys89)	5.48			
	Alkyl-Alkyl (Lys89)	5.49			
**11h**	Hydrogen bond (Asp86)	2.13	**11m**	Hydrogen bond (Leu83)	2.02
	Hydrogen bond (Leu83)	2.59		Alkyl-Alkyl (Lys89)	5.34
	Halogen interaction (Asp145)	3.15		Halogen interaction (Ile10)	2.94
	Halogen interaction (Asp145)	3.19		Halogen interaction (Ile10)	3.10
	Halogen interaction (Asn132)	3.14		Halogen interaction (Asp86)	3.00
	Halogen interaction (Leu83)	2.86		Halogen interaction (Asp86)	3.27
	Halogen interaction (Leu83)	3.17		Halogen interaction (Gln131)	2.80
	Pi-Alkyl (Ile10)	4.30		Pi-Alkyl (Ile10)	3.94
	Alkyl-Alkyl (Ile10)	4.71		Alkyl-Alkyl (Ile10)	5.42
	Pi-Alkyl (Leu134)	5.04		Non-classical hydrogen bond (Ile10)	3.48
	Alkyl-Alkyl (Leu134)	4.37		Alkyl-Alkyl (Lys33)	5.28
	Pi-Alkyl (Phe82)	5.02		Non-classical hydrogen bond (Phe82)	2.66
	Pi-Alkyl (Val18)	4.76		Non-classical hydrogen bond (Glu81)	3.74
	Alkyl-Alkyl (Val18)	4.43		Pi-Sigma interaction (Phe80)	3.50
	Pi-Alkyl (Ala144)	3.97		Alkyl-Alkyl (Val18)	5.02
	Pi-Pi interaction (Phe80)	5.14		Alkyl-Alkyl (Val64)	5.03
	Non-classical hydrogen bond (Lys33)	2.79		Alkyl-Alkyl (Ala144)	4.29
				Alkyl-Alkyl (Ala31)	5.00
				Pi-Alkyl interaction (Leu134)	4.96

#### 2.4.2. *In vitro* CDK2 kinase assay

On account of their best scores achieved in the docking study (Table 3) as well as their efficient anti-proliferative actions towards both the examined breast cancer cell lines (Table 1), pyridazines **11e**, **11h**, **11l**, and **11m** were selected to be examined for their ability to inhibit CDK2. The results have been obtained as IC_50_ values which listed in Table 5.

**Table 5.** IC_50_ values for the CDK2 inhibitory action of pyridazines **11e**, **11h**, **11l**, and **11m**.

Comp.	IC_50_ (nM)
	CDK2
**11e**	151 ± 6.16
**11h**	43.8 ± 1.79
**11l**	55.6 ± 2.27
**11m**	20.1 ± 0.82
**Staurosporine**	12.4 ± 0.51

The results revealed that the examined pyridazines **11e**, **11h**, **11l**, and **11m** possessed good inhibitory action towards CDK2 kinase with IC_50_ values equal 151 ± 6.16, 43.8 ± 1.79, 55.6 ± 2.27 and 20.1 ± 0.82 nM, respectively (Table 5). Remarkably pyridazine derivative **11m**, bearing two morpholine moieties, elicited the best CDK2 inhibitory activity in this study (IC_50_ = 20.1 ± 0.82 nM), alongside to its superior anti-proliferative activity that has been reported above (Table 1). It’s worth stressing that this is the first study, to the best of our knowledge, that reports on 3,6-disubstituted pyridazines as anticancer CDK inhibitors.

### 2.5. In silico ADME calculation

As a rule, novel small molecules considered as potential drug candidates when they possess acceptable pharmacokinetic and pharmacodynamic profiles. Thus, assessment of the efficiency for the synthesised compounds should rely not only on basis of their biological activities but also with taking in consideration their pharmacokinetics, druglikness and physicochemical properties.

**Figure 11.**
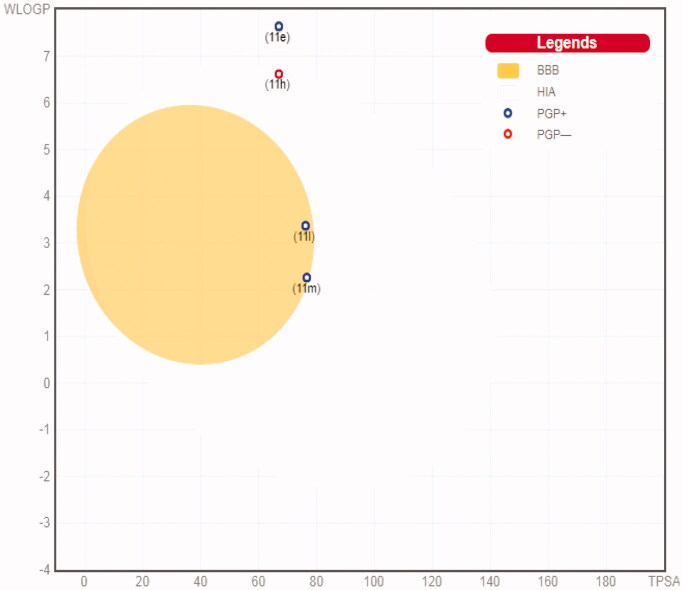
Boiled-Egg chart for pyridazines **11e**, **11h**, **11l**, and **11m**.

The SwissADME online tool has been adopted to calculate the ADME profiles for target pyridazines **11e**, **11h**, **11l** and **11m**^38^. Target pyridazines **11h**, **11l** and **11m** were predicted to have high gastrointestinal tract (GIT) absorption, as they were present within the region of human intestinal absorption (HIA) in the BOILED-Egg chart^39^, whereas compound **11e** was located outside the region of HIA in the BOILED-Egg chart (Figure 11) and thus predicted to possess low GIT absorption that may hinder its oral bioavailability. The low GIT absorption predicted for compound **11e** could be attributed to the pronounced lipophilicity of the rigid polycyclic cage hydrocarbon adamantine, as well as incorporation of *ortho*-trifluoromethyl phenyl moiety. At the same time, the BOILED-Egg graph (Figure 11) pointed out that target pyridazines **11e** and **11h** have no BBB permeability and thus could be used as antitumor agents with no predicted CNS concerns, whereas, compounds **11l** and **11m** have the ability to penetrate through the BBB and so they could be of great value for brain malignancies (Figure 11).

**Figure 12.**
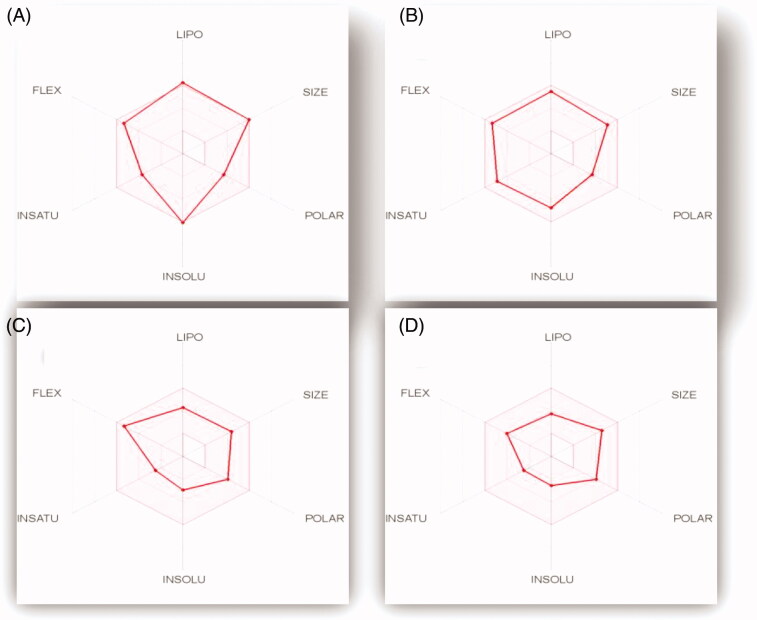
The oral bioavailability radar chart for pyridazines **11e (A)**, **11h (B)**, **11l (C)** and **11m (D)**, produced by swissADME online web tool.

The bioavailability radar plot (Figure 12) explains the extent of GIT absorption for the four examined pyridazines **11e**, **11h**, **11l** and **11m**. The radar chart comprises six critical parameters for oral bioavailability; LIPO (Lipophilicity), INSOLU (Solubility), INSATU (saturation), FLEX (Flexibility), SIZE (SIZE), and POLAR (polarity). The pink area of the radar chart represents the optimal range for each property value within the six properties, while the red lines represents the predicted physicochemical features for the examined pyridazines **11e**, **11h**, **11l** and **11m**, Figure 12.

All the predicted physicochemical properties for pyridazines **11e**, **11h**, **11l** and **11m** were located in the desired pink area for all the six parameters, with an exception for compound **11e** which barely violated the LIPO (Lipophilicity) and INSOLU (Solubility) parameters (Figure 12), which clarifies its location outside the region of HIA in the BOILED-Egg graph. The other three compounds (**11h**, **11l** and **11m**) have the optimal combination of physicochemical properties that guaranteed a high GIT absorption with no single violation to any component of the bioavailability radar chart.

The metabolism for the four pyridazines **11e**, **11h**, **11l** and **11m** are predicted to take place partially in the liver by one or more of the five major Cytochrome P (CYP) isoforms (CYP2C9, CYP1A2, CYP2D6, CYP2C19, CYP3A4), and thus target pyridazines are suggested to be administered alone to minimise the possible drug-drug interactions. In details, pyridazines **11e**, **11h** and **11l** are predicted to exert inhibition for two of CYP isoforms. Compound **11e** was predicted to inhibit CYP2D6 and CYP3A4, compound **11h** was predicted to inhibit CYP2C19 and CYP3A4, and compound **11l** was predicted to inhibit CYP1A2 and CYP2D6. Furthermore, compound **11m** was predicted to inhibit CYP1A2 only (Table 6).

**Table 6.** The *in silico* predicted ADME for pyridazines **11e**, **11h**, **11l**, and **11m**.

Cpd.	BBB	GIA	P-gP substrate	CYP1A2 inhibitor	CYP2C19 inhibitor	CYP2C9 inhibitor	CYP2D6 inhibitor	CYP3A4 inhibitor
**11e**	No	Low	Yes	No	No	No	Yes	Yes
**11h**	No	High	No	No	Yes	No	No	Yes
**11l**	Yes	High	Yes	Yes	No	No	Yes	No
**11m**	Yes	High	Yes	Yes	No	No	No	No

GIA: gastrointestinal absorption; BBB: Blood Brain Barrier; PgP: P-glyco protein transporter.

On another note, SwissADME online web tool revealed that all the examined pyridazines were found to comply with all druglikeness rules defined by the pioneer pharmaceutical companies; Veber’s (GSK)^40^, Lipinski’s (Pfizer)^41^, Egan’s (Pharmacia)^42^, Ghose’s (Amgen)^43^ and Muegge’s (Bayer)^44^ filters, with an exception for compound **11e** (towards Egan, Ghose and Muegge rules) and compound **11h** (towards Egan and Ghose rules) (Table 7). Interestingly enough, from the medicinal chemistry point of view the four examined pyridazines have neither PAINS (Pan Assay Interference Structures)^45^ alerts nor Brenks (Structural) alerts^46^, which emphasises that their chemical structures lack any interfering framework that could result in an artefact in any protein assay implying that the obtained results from the *in vitro* biological assays are to be robust.

**Table 7.** Compliance with Druglikeness rules (Lipinski, Veber, Egan, Ghose and Muegge), as well as PAINS and Brenk filters for target pyridazines **11e**, **11h**, **11l**, and **11m**.

Cpd.	Lipinski violations	Veber violations	Muegge violations	Egan violations	Ghose violations	PAINS alerts	Brenk alerts
**11e**	Yes	Yes	No	No	No	0	0
**11h**	Yes	Yes	Yes	No	No	0	0
**11l**	Yes	Yes	Yes	Yes	Yes	0	0
**11m**	Yes	Yes	Yes	Yes	Yes	0	0

PAINS: Pan Assay Interference Structures.

In conclusion, the four lead pyridazines (**11e**, **11h**, **11l** and **11m**) proved not only efficient biological actions but also an acceptable ADME and physicochemical properties, in particular pyridazines **11l** and **11m**.

## 3. Materials and methods

### 3.1. Chemistry

#### 3.1.1. General

Melting points have been obtained on a Büchi Melting PointB-540 apparatus which uncorrected and determined. ^1^H NMR spectra have been recorded on a Bruker Advance III instrument at 400 MHz. Chemical shifts (*δ*) of NMR have been reported in parts per million (*ppm*) units relative to internal standard (TMS). All reactions have been monitored by thin-layer chromatography (TLC), using silica gel plates (GF254) and UV light visualisation. Flash chromatography separations have been obtained on silica gel (200–300 mesh).

#### 3.1.2. Synthesis of ethyl 2-hydroxy-4-oxo-2-(trifluoromethyl)pentanoate (2)

Compound 2 was reported previously^31^.

#### 3.1.3. Synthesis of 6-methyl-4-(trifluoromethyl)pyridazin-3(2H)-one (3)

A solution of ethyl 2-hydroxy-4-oxo-2-(trifluoromethyl)pentanoate **2** (0.45 g, 2 mmol) and 99% hydrazine monohydrate (0.53 ml, 11 mmol) in acetic acid (7 ml), was refluxed with stirring for 6 h. The solution was then poured into H_2_O and extracted with EtOAc, dried by NaSO_4_ and purified by column flash chromatography (petroleum ether/EtOAc 70:30) to produce pyridazinone **3** as white crystals (0.27 g, 69%); m.p. 184–185 °C (reported m.p. 187–189 °C^32^).

#### 3.1.4. Synthesis of 6-oxo-5-(trifluoromethyl)-1,6-dihydropyridazine-3-carboxylic acid (4)

Pyridazinone **3** (0.1 g, 0.561 mmol) was dissolved in conc. H_2_SO_4_ (5 ml) and stirred at 0 °C, then K_2_Cr_2_O_7_ (0.24 g, 0.846 mmol) was added portionwise. The reaction mixture was left to be stir at r.t. overnight, then added to ice-water and extracted to be extracted with ethyl acetate. The organic phases were dried with NaSO_4_ and concentrated under reduced pressure and the residue was purified by flash chromatography (DCM/MeOH: 90/10) to obtain pyridazine-3-carboxylic acid derivative **4** as white crystals (0.06 g, 54%); m.p. 239–240 °C.

#### 3.1.5. Synthesis of ethyl 6-oxo-5-(trifluoromethyl)-1,6-dihydropyridazine-3-carboxylate (5)

Dihydropyridazine-3-carboxylic acid derivative **4** (2.4 g, 11.5 mmol) was dissolved in ethanol (18 ml) and H_2_SO_4_ (0.5 ml), then the reaction mixture was heated under reflux for 4 h. The solution was extracted with ethyl acetate and purified by column chromatography (petroleum ether / EtOAc 70:30) to produce white solid (3.0 g, 90%); m.p. 147–148 °C.

#### 3.1.6. Synthesis of ethyl 6-chloro-5-(trifluoromethyl) pyridazine-3-carboxylate (6)

Ethyl 6-oxo dihydropyridazine-3-carboxylate derivative **5** (2 g, 8.47 mmol) was added portionwise to a cooled stirred phosphorus oxychloride (POCl_3_) (10 ml) which subsequently heated at 100 °C for 5 h. The reaction mixture, after cooling, was poured into iced-water, neutralised by aqueous solution of sodium hydroxide, extracted with ethyl acetate and purified by column chromatography (petroleum ether/EtOAc 85:15) to yield ester **6** as yellow oil (1.1 g, 51%).

#### 3.1.7. General procedures for preparation of 6-chloro-5-(trifluoromethyl)pyridazine-3-carboxamide derivatives (9a–e)

Ethyl 6-chloro-5-(trifluoromethyl)pyridazine-3-carboxylate **6** (0.04 g, 0.16 mmol) was added to a mixture of THF/H_2_O (4:1) and LiOH hydrate (0.80 mmol) at 0 °C, then the reaction mixture was stirred for 1 h at room temperature. The reaction mixture was then evaporated to dryness and the residue was dissolved in H_2_O, neutralised carefully with a 1 N HCl, and extracted with ethyl acetate. Organic layers were collected, dried over anhydrous sodium sulphate, filtered and the solvent was removed under reduced pressure to yield the corresponding carboxylic acid. The later acid derivative was dissolved in dry 1,2-dichloroethane (5 ml), then thionyl chloride (0.9 mmol) and DMF (2–3 drops) were added to the solution. The mixture was refluxed for 3 h then evaporated to dryness to produce the crude product of acyl chloride **7**. This compound was used immediately for the next step to prepare key amide intermediates **9a–e** *via* stirring with primary amines **8a–e** (0.32 mmol) in methylene chloride (4 ml) and in the presence of TEA (0.80 mmol) for 4 h at room temperature. The obtained precipitate was filtered off and washed with petroleum ether, then used in the next step without further purification.

#### 3.1.8. Synthesis of ethyl 6-morpholino-5-(trifluoromethyl)pyridazine-3-carboxylate (13)

A mixture of ethyl 6-chloro-5-(trifluoromethyl)pyridazine-3-carboxylate **6** (0.06 g, 0.23 mmol), morpholine **12** (0.1 g, 1.12 mmol) and Hünig’s base (0.29 g, 2.24 mmol) were dissolved in 1,4-dioxane (10 ml), and the reaction mixture was heated under reflux for 12 h. After cooling to room temperature, the reaction mixture was evaporated under reduced pressure and the residue was dissolved in ethyl acetate and washed with H_2_O. The organic layer was then dried, evaporated *in vacuo* and purified with flash chromatography on silica gel (petroleum ether/EtOAc 70/30) to obtain compound **13** as yellow oil (0.0476 g, 70%).

#### 3.1.9. General procedures for the preparation of target pyridazine derivatives (11a-r)

##### 3.1.9.1. Route (A)

The appropriate aliphatic amine **8a**, **8e** and **10a**–**d** (5 mmol) and Hünig’s base (1.3 g, 10 mmol) were added to a solution of intermediates **9a–e** (1 mmol) in dioxane (5 ml), and then the reaction mixture was refluxed for 6 h. After the reaction completed, the excess dioxane was evaporated under reduced pressure, the residue was dissolved in ethyl acetate and washed with water and the organic layer was dried over anhydrous Na_2_SO4, and evaporated *in vacuo*. The crude product was purified by flash chromatography (petroleum ether: ethyl acetate 70:30) to yield target pyridazine **11a–r**.

##### 3.1.9.2. Route (B)

Ethyl 6-morpholino-5-(trifluoromethyl)pyridazine-3-carboxylate **(13)** (2.54 g, 10 mmol) was dissolved in ethyl alcohol (8 ml), then primary amines (**8a**–**e**) (15 mmol) and piperidine (2–3 drops) were added. The reaction mixture was refluxed for 6 h and then the solvent was evaporated under reduce pressure. The crude product was purified by using flash column chromatography (petroleum ether: ethyl acetate 70:30) to afford the corresponding target pyridazines **11d**, **11g**, **11i**, **11m** and **11r**, respectively^33^.

For the full characterisation details for the intermediates and the target pyridazines 11a-r have been provided in the Supplementary Materials.

### 3.2. Biological evaluations

The utilised procedures in the biological assays were performed as described earlier; cytotoxicity^47^, cell cycle^48^, Annexin V-FITC Apoptosis^49^ and CDK2^50^ assays, whereas all detailed procedures were mentioned in the Supplementary Materials.

### 3.3. In silico studies

#### 3.3.1. In silico target prediction

To deduce a potential mechanism of action for herein reported target pyridazines **11**, the SwissTargetPrediction online tool was utilised^35^. Two representative compounds **11a** and **11j** were drawn and submitted to the server for target prediction.

#### 3.3.2. Docking studies

The crystal structure of CDK2 in complex with roniciclib was collected from the protein data bank PDB ID (5iev) ^51^. The molecular docking procedures reliability was ensured through re-docking of the co-crystalized roniciclib in the vicinity of the CDK2 binding site, as mentioned above in the discussion section. Molecular docking of the target pyridazines into CDK2 active site was conducted by Vina Autodock, a more accurate and twice speed higher than Autodock 4 software^52^. Vina Autodock requires both ligands and receptors in pdbqt format, thus, MGL tools 1.5.7 were used to prepare all the necessary files to carry out the docking^53^. Also the MGL tools were used to generate a grid box around the binding site of Roniciclib. The docking results were visualised by Biovia discovery studio 2020 free visualiser^54^, and then the best scoring candidates were selected for further investigation.

#### 3.3.3. In silico ADME calculation and Drug-Likeness properties prediction

The SwissADME online tool was utilised to calculate the ADME profiles for target pyridazines **11e**, **11h**, **11l** and **11m**, in addition to prediction of their medicinal chemistry friendliness and drug-likeness properties^38^.

## 4. Conclusions

To the best of our knowledge, this is the first study that reports on 3,6-disubstituted pyridazines as anticancer CDK inhibitors. Herein, a new series of 3,6-disubstituted pyridazines **11a–r** has been synthesised, characterised and evaluated for *in vitro* anticancer activity against three human cancer cell lines, namely, T-47D (breast cancer), MDA-MB-231 (breast cancer) and SKOV-3 (ovarian cancer) cell lines by the SRB assay. While, the examined pyridazines elicited good activity against T-47D cells (IC_50_ range: 0.43 ± 0.01 − 35.9 ± 1.18 µM) and MDA-MB-231 cells (IC_50_ range: 0.99 ± 0.03 − 34.59 ± 1.13 µM), they exerted weak activity against SKOV-3 cells. Uniquely, the methyltetrahydropyran-bearing pyridazine **11m** showed a submicromolar growth inhibitory potency towards both breast T-47D and MDA-MB-231 (IC_50_ = 0.43 ± 0.01 and 0.99 ± 0.03 µM, respectively) cell lines. In addition, the biological results indicated that pyridazines **11l** and **11m** exerted an efficient alteration in cell cycle progression and induction of apoptosis in both T-47D and MDA-MB-231 cells, alongside, with their good mean tumour selectivity indexes (13.7 and 16.1, respectively) upon assessment of their cytotoxicity towards non-tumorigenic breast MCF-10A cells. Based on a suggestion from a conducted *in silico* study, pyridazines **11e**, **11h**, **11l**, and **11m** were selected to be evaluated for their ability to inhibit CDK2, where they exerted good inhibitory activity (IC_50_ = 151, 43.8, 55.6 and 20.1 nM, respectively). Finally, the *in silico* study implied that target pyridazines **11** exhibited not only an efficient anticancer activity but also an acceptable ADME, physicochemical and druglikeness properties, specifically pyridazines **11l** and **11m**. Overall the obtained results from this study quite sustained our strategy and gave us a robust opportunity for further development and optimisation of 3,6-disubstituted pyridazine scaffold to enrich therapeutic arsenal with efficient and safe anticancer CDK inhibitors.

## Supplementary Material

Supplemental Material
