# Supplementary material for: Discovery of 3,6-disubstituted pyridazines as a novel class of anticancer agents targeting cyclin-dependent kinase 2: synthesis, biological evaluation and in silico insights
Source: J Enzyme Inhib Med Chem. 2020 Aug 11;35(1):1616–30. doi: 10.1080/14756366.2020.1806259 (PMC7470104; doi:10.1080/14756366.2020.1806259)
Supplement: Supplemental Material [file IENZ_A_1806259_SM2316.pdf]

Supporting Information

Discovery of 3,6-Disubstituted Pyridazines as a Novel Class of Anticancer Agents Targeting Cyclin-Dependent Kinase 2: Synthesis, Biological Evaluation and In Silico Insights

Ahmed Sabt, Wagdy M. Eldehna*, Tarfah Al-Warhi, Ohoud J. Alotaibi, Mahmoud M. Elaasser, Howayda Suliman, Hatem A. Abdel-Aziz

Tables of Contents

**1.**	Characterization of intermediate (**2**, **3**, **4**, **5**, **6** and **13**) and target pyridazines **11a-r**	3
**2.**	Anti-proliferative Activity	11
**3.**	Cell Cycle Analysis	11
**4.**	AnnexinV-FITC/PI Apoptosis Assay	12
**5.**	CDK Kinase Inhibitory Activity Statistical Analysis	12
**6.**	Statistical Analysis	12
**7.**	Figure S1. Different target classes suggested by SwissTargetPrediction online tool	13
**8.**	Table S1. Different targets suggested by SwissTargetPrediction online tool	14-18
**9.**	NMR Spectra	19-68

1. Characterization of intermediate (2, 3, 4, 5, 6 and 13) and target pyridazines 11a-r

Ethyl 2-hydroxy-4-oxo-2-(trifluoromethyl)pentanoate (*2*)

Yellow oil; ^{1}H NMR ($CDCl_3$, 400 MHz) *δ ppm*: 1.20 (t, 3H, *J* = 7.2 Hz, CH_3), 2.11 (s, 3H, CH_3), 3.03 (q, 2H, *J* = 17.6Hz, OCH_2), 4.19 (brs, 1H, OH), Ha 4.24 (d, 1H, *J* = 2.4 Hz), Hb 4.26 (d, 1H, *J* = 2.4 Hz).

6-Methyl-4-(trifluoromethyl)pyridazin-3(2*H*)-one (*3*)

White crystals (0.27 g, 69%); m.p. 184-185 °C (reported m.p. 187-189 °C [32]); ^{1}H NMR ($CDCl_3$, 400 MHz) *δ ppm*: 2.42 (s, 3H,CH_3), 7.49 (s, 1H, CH), 12.44 (brs, 1H,NH); ^{13}C NMR ($CDCl_3$, 100 MHz) *δ ppm*: 20.67 (CH_3), 116.92 (q, $J_{(C,F)}$ = 271.4 Hz, CF_3), 128.90 (q, $J_{(C,F)}$ = 32.7 Hz, C4), 132.38 (q, $J_{(C,F)}$ = 4.6 Hz, C5), 144.69 (C=N), 157.58 (C=O); ^{19}F NMR ($CDCl_3$, 376 MHz) *δ ppm*: 67.33 (s, 3F).

6-Oxo-5-(trifluoromethyl)-1,6-dihydropyridazine-3-carboxylic acid (4)

White crystals (0.06 g, 54%); m.p. 239-240 °C; ^{1}H NMR (DMSO-d_6, 400 MHz) *δ ppm*: 3.17 (brs, 1H, NH), 8.20 (s, 1H, CH-pyridazinone), 14.18 (s, 1H, COOH); Analysis for $C_6H_3F_3N_2O_3$ (208), Calcd.: % C, 34.63; H, 1.45; N, 13.46; Found: C, 34.51; H, 1.47; N, 13.52.

Ethyl 6-oxo-5-(trifluoromethyl)-1,6-dihydropyridazine-3-carboxylate (*5*)

White solid (3.0 g, 90%); m.p. 147-148 ºC; ^{1}H NMR ($CDCl_3$, 400 MHz) *δ ppm*: 1.35 (t, 3H, *J* = 6.8 Hz, CH_3), 4.39 (q, 2H, *J* = 7.2 Hz, CH_2), 7.9 (brs, 1H, NH), 8.18 (s, 1H, CH-pyridazine), 12.26 (brs, 1H, NH); Analysis for $C_8H_7F_3N_2O_3$ (236), Calcd.: % C, 40.69; H, 2.99; N, 11.86; Found: C, 40.56; H, 3.00; N, 11.91.

Ethyl 6-chloro-5-(trifluoromethyl) pyridazine-3-carboxylate (*6*)

Yellow liquid (1.1 g, 51%); ^{1}H NMR ($CDCl_3$, 400 MHz) *δ ppm*: 1.49 (t, 3H, *J* = 7.2 Hz, CH_3), 4.57 (q, 2H, *J* = 7.2 Hz, OCH_2), 8.44 (s, 1H, CH-pyridazine); ^{13}C NMR ($CDCl_3$, 100 MHz) *δ ppm*: 14.09 (CH_3), 63.42 (OCH_2), 116.**59** (q, $J_{(C,F)}$ = 27**3** Hz, CF_3), 12**6.31** (q, $J_{(C,F)}$

= 4.7 Hz, C4), 128.86 (q, $J_{(C,F)}$ = 35.6 Hz, C5), 151.45 (C=N), 154.95 (C-Cl), 162.16 (C=O); Analysis for $C_8H_6ClF_3N_2O_2$ (254), Calcd.:C, 37.74; H, 2.38; N, 11.00; Found: C, 37.82; H, 2.36; N, 10.95.

Ethyl 6-morpholino-5-(trifluoromethyl)pyridazine-3-carboxylate (*13*)

Yellow oil (0.0476 g, 70%); ^{1}H NMR ($CDCl_3$, 400 MHz) δ *ppm*: 1.37 (t, 3H, *J* = 7.2 Hz, CH_3), 3.68 (t, 4H, *J* = 4.4 Hz, 2xCH_2-N), 3.78 (t, 4H, *J* = 5.2 Hz, 2xCH_2-O), 4.41 (q, 2H, *J* = 7.2 Hz, OCH_2), 8.11 (s, 1H, CH-pyridazine); Analysis for $C_{12}H_{14}F_3N_3O_3$ (305), Calcd.:C, 47.22; H, 4.62; N, 13.77; Found: C, 47.40; H, 4.59; N, 13.93.

Target pyridazine derivatives (*11a-r*)

N-(Adamantan-2-yl)-6-((2-hydroxyethyl)amino)-5-(trifluoromethyl)pyridazine-3-carboxamide (11a)

Pale yellow crystals; Yield (63%); m.p. 202-203 °C; ^{1}H NMR ($CDCl_3$, 400 MHz) δ *ppm*: 1.59 (d, *J* = 12.8 Hz, 2H), 1.70 (s, 2H), 1.83 (s, 6H), 1.86 (d, *J* = 13.6 Hz, 2H), 1.96 (brs, 2H), 2.60 (brs, 1H, OH), 3.83 (t, 2H, *J* = 5.2 Hz, CH_2-NH), 3.87 (t, 2H, *J* = 4.8 Hz, CH_2-OH), 4.17 (d, 1H, *J* = 8.4 Hz, 1H-adamantyl), 5.71 (brs, 1H, NH), 8.14 (s, 1H, CH-pyridazine), 8.18 (d, 1H, *J* = 8.4 Hz, NH); ^{13}C NMR ($CDCl_3$, 100 MHz) δ *ppm*: 26.10, 26.20, 30.85 (2xC), 31.07 (2xC), 36.10 (2xC), 36.49, 43.45 (CH_2-NH), 52.54 (NH–C-adamantyl), 60.23 (CH_2-OH), 113.20 (q, $J_{(C,F)}$ = 33.7 Hz, C5), 117.56 (q, $J_{(C,F)}$ = 272 Hz,CF_3), 122.67 (q, $J_{(C,F)}$ = 5.0 Hz, C4), 144.37 (C=N), 153.84 (C=O), 160.22 (-N-C=N); Analysis for $C_{18}H_{23}F_3N_4O_2$ (384), Calcd.: C, 56.24; H, 6.03; N, 14.58; Found: C, 56.47; H, 5.98; N, 14.64.

N-(Adamantan-2-yl)-6-(butylamino)-5-(trifluoromethyl)pyridazine-3-carboxamide (11b)

Yellow solid; Yield (72%); m.p. 110-111°C; ^{1}H NMR ($CDCl_3$, 400 MHz) δ *ppm*: 0.89 (t, 3H, *J* = 7.2 Hz, CH_3), 1.34-1.43 (m, 2H, CH_2), 1.55 (d, *J* = 13.6 Hz, 2H), 1.62-1.70 (m, 4H), 1.83 (s, 6H), 1.88 (d, *J* = 13.2 Hz, 2H), 1.97 (s, 2H), 3.64 (d, 2H, *J* = 4.0 Hz, CH_2-NH), 4.18 (d, 1H, *J* = 8.4 Hz, 1H-adamantyl), 5.11 (brs, 1H, NH), 8.12 (s, 1H, CH-pyridazine), 8.19 (d, 1H, *J* = 8.0 Hz, NH-C=O); ^{13}C NMR ($CDCl_3$, 100 MHz) δ *ppm*: 12.74 (CH_3), 19.07,

26.14, 26.22, 30.07, 30.85 (2xC), 31.09 (2xC), 36.13 (2xC), 36.52, 41.13 (CH_2-NH), 52.47 (NH–C-adamantyl), 112.58 (q, $J_{(C,F)}$ = 33.5 Hz, C5), 120.46 (q, $J_{(C,F)}$ = 271.5 Hz, CF_3), 122.45 (q, $J_{(C,F)}$ = 5.0 Hz, C4), 144.05 (C=N), 153.52 (C=O), 160.31 (-N-C=N); Analysis for $C_{20}H_{27}F_3N_4O$ (396), Calcd.: C, 60.59; H, 6.86; N, 14.13; Found: C, 60.67; H, 6.91; N, 14.05.

N-(Adamantan-2-yl)-6-(cyclopentylamino)-5-(trifluoromethyl)pyridazine-3-carboxamide (11c)

White solid; Yield (36%); m.p. 135-136 °C; ^{1}H NMR ($CDCl_3$, 400 MHz) *δ ppm*: 1.44-1.52 (m, 2H, CH_2), 1.58 (d, 2H, *J* = 13.2 Hz), 1.63-1.69 (m, 6H), 1.83 (s, 6H), 1.88 (d, *J* = 13.2 Hz, 2H), 1.96 (s, 2H), 2.11-2.19 (m, 2H, CH_2), 4.18 (d, 1H, *J* = 8.0 Hz, 1H-adamantyl), 4.57-4, 65 (m, 1H, CH-cyclopentyl), 5.05 (d, 1H, *J* = 6.0 Hz, NH), 8.11 (s, 1H, CH-pyridazine), 8.19 (d, 1H, *J* = 8.4 Hz, NH); ^{13}C NMR ($CDCl_3$, 100 MHz) *δ ppm*: 22.70 (2xC), 26.15, 26.23, 30.84 (2xC), 31.09 (2xC), 32.25 (2xC), 36.14 (2xC), 36.53, 52.49 (CH-cyclopentyl), 52.83 (NH–C-adamantyl), 112.59 (q, $J_{(C,F)}$ = 33.4 Hz, C5), 117.74 (q, $J_{(C,F)}$ = 271.6 Hz, CF_3), 122.44 (q, $J_{(C,F)}$ = 5.1 Hz, C4), 143.92 (C=N), 153.22 (C=O), 160.35 (-N-C=N); Analysis for $C_{21}H_{27}F_3N_4O$ (408), Calcd.: C, 61.75; H, 6.66; N, 13.72; Found: C, 61.61; H, 6.63; N, 13.68.

N-(Adamantan-2-yl)-6-morpholino-5-(trifluoromethyl)pyridazine-3-carboxamide (11d)

White solid; Yield (50%); m.p. 177-178 °C; ^{1}H NMR ($CDCl_3$, 400 MHz) *δ ppm*: 1.60 (d, *J* = 12.8 Hz, 2H), 1.70 (s, 2H), 1.84 (s, 6H), 1.87 (d, *J* = 13.6 Hz, 2H), 1.98 (s, 2H), 3.57 (t, 4H, *J* = 4.0 Hz, 2xCH_2-N), 3.79 (t, 4H, *J* = 4.4 Hz, 2xCH_2-O), 4.20 (d, 1H, *J* = 8.0 Hz, 1H-adamantyl), 8.24 (d, 1H, *J* = 8.0 Hz, NH-C=O), 8.28 (s, 1H, CH-pyridazine); ^{13}C NMR ($CDCl_3$, 100 MHz) *δ ppm*: 26.10, 26.20, 30.86 (2xC), 31.08 (2xC), 36.09 (2xC), 36.48, 49.32 (2xCH_2-N), 52.60 (adamantyl-C-NH), 65.57 (2xCH_2-O), 117.63 (q, $J_{(C,F)}$ = 272.3 Hz, CF_3), 117.83 (q, $J_{(C,F)}$ = 34.1 Hz, C5), 124.98 (q, $J_{(C,F)}$ = 5.2 Hz, C4), 146.54 (C=N), 157.54 (C=O), 159.75 (-N-C=N); Analysis for $C_{20}H_{25}F_3N_4O_2$ (410), Calcd.: C, 58.53; H, 6.14; N, 13.65; Found: C, 58.45; H, 6.15; N, 13.69.

N-(Adamantan-2-yl)-5-(trifluoromethyl)-6-((2(trifluoromethyl)benzyl)amino)pyridazine-3-carboxamide (11e)

White solid; Yield (41%); m.p. 150-151 °C; ^{1}H NMR ($CDCl_3$, 400 MHz) *δ ppm*: 1.59 (d, *J* = 12.4 Hz, 2H), 1.70 (s, 2H), 1.83 (s, 6H), 1.87 (d, *J* = 13.2 Hz, 2H), 1.97 (brs, 2H), 4.18-4.20 (m, 1H, 1H-adamantyl), 5.09 (d, *J* = 5.6 Hz, 2H, CH_2-NH), 5.50 (t-like, 1H, *J* = 5.6 Hz, NH), 7.34 (t, 1H, *J* = 7.6 Hz, C5-Ar), 7.45 (t, 1H, *J* = 7.6 Hz, C4-Ar), 7.53 (d, 1H, *J* = 8.00 Hz, C6-Ar), 7.63 (d, 1H, *J* = 8.00 Hz, C3-Ar), 8.18 (s, 1H, CH-pyridazine), 8.20 (d, 1H, *J* = 3.6 Hz, NH-CO); ^{13}C NMR ($CDCl_3$, 100 MHz) *δ ppm*: 26.11, 26.20, 30.85 (2xC), 31.05 (2xC), 31.09, 36.11 (2xC), 36.49, 40.51, 41.69 (CH_2-NH), 52.57 (NH–C-adamantyl), 113.03 (q, $J_{(C,F)}$ = 33.6 Hz, C5), 120.29 (q, $J_{(C,F)}$ = 271.9 Hz,CF_3-pyridazine), 122.20 (q, $J_{(C,F)}$ = 256.5 Hz, CF_3-Ar), 122.72 (q, $J_{(C,F)}$ = 4.9 Hz, C4), 124.49 (q, $J_{(C,F)}$ = 26.7 Hz, C2-Ar), 125.38 (q, $J_{(C,F)}$ = 5.8 Hz, C3-Ar), 127.04 (C5-Ar), 129.19 (C4-Ar), 131.40 (C6-Ar), 134.55 (C1-Ar), 144.92 (C=N), 153.16 (C=O), 160.11 (-N-C=N); Analysis for $C_{24}H_{24}F_6N_4O$ (498), Calcd.: C, 57.83; H, 4.85; N, 11.24; Found: C, 57.94; H, 4.88; N, 11.19.

N-(tert-Butyl)-6-(cyclopentylamino)-5-(trifluoromethyl)pyridazine-3-carboxamide (11f)

Brown solid; Yield (71%); m.p. 62-63 °C; ^{1}H NMR ($CDCl_3$, 400 MHz) *δ ppm*: 1.40 (s, 9H, $3xCH_3$), 1.43-1.51 (m, 2H, CH_2), 1.58-1.72 (m, 4H, $2xCH_2$), 2.11-2.18 (m, 2H, CH_2), 4.53-4.61 (m, 1H, CH-cyclopentyl), 5.05 (brs, 1H, NH), 7.76 (brs, 1H, NH-C=O), 8.10 (s, 1H, CH-pyridazine); ^{13}C NMR ($CDCl_3$, 100 MHz) *δ ppm*: 22.72 ($2xCH_2$), 27.78 ($3xCH_3$), 32.22 ($2xCH_2$-CH), 50.32 (NH-C-t-butyl), 52.87 (CH-NH), 112.68 (q, $J_{(C,F)}$ = 33.6 Hz, C5), 120.43 (q, $J_{(C,F)}$ = 271.4 Hz, CF_3), 122.10 (q, $J_{(C,F)}$ = 5.0 Hz, C4), 144.20 (C=N), 153.11 (C=O), 160.40 (-N-C=N); Analysis for $C_{15}H_{21}F_3N_4O$ (330), Calcd.: C, 54.54; H, 6.41; N, 16.96; Found: C, 54.72; H, 6.38; N, 17.05.

N-(tert-Butyl)-6-morpholino-5-(trifluoromethyl)pyridazine-3-carboxamide (11g)

Light yellow solid; Yield (73%); m.p. 121-122 °C; ^{1}H NMR ($CDCl_3$, 400 MHz) *δ ppm*: 1.43 (s, 9H, $3xCH_3$), 3.56 (t, 4H, *J* = 4.4 Hz, $2xCH_2$-N), 3.79 (t, 4H, *J* = 4.8 Hz, $2xCH_2$-O), 7.79 (brs, 1H, NH), 8.27 (s, 1H, CH-pyridazine); ^{13}C NMR ($CDCl_3$, 100 MHz) *δ ppm*: 27.74 ($3xCH_3$), 49.30 ($2xCH_2$-N), 50.53 (C-NH), 65.55 ($2xCH_2$-O), 117.61 (q, $J_{(C,F)}$ = 272.4 Hz, CF_3), 117.92 (q, $J_{(C,F)}$ = 34.1 Hz, C5), 124.66 (q, $J_{(C,F)}$ = 5.2 Hz, C4), 146.86 (C=N), 157.44 (C=O), 159.79 (-N-C=N); Analysis for $C_{14}H_{19}F_3N_4O_2$ (332), Calcd.: C, 50.60; H, 5.76; N, 16.86; Found: C, 50.49; H, 5.77; N, 16.90.

N-(tert-Butyl)-5-(trifluoromethyl)-6-((2-(trifluoromethyl) benzyl) amino)pyridazine-3-carboxamide (11h)

White solid; Yield (43%); m.p. 132-133 °C; ^{1}H NMR ($CDCl_3$, 400 MHz) *δ ppm*: 1.40 (s, 9H, $3xCH_3$), 5.07 (d, 2H, J = 6.0 Hz, CH_2-NH), 5.51 (t-like, 1H, J = 6.0 Hz, NH), 7.33 (t, 1H, J = 7.6 Hz, C5-Ar), 7.44 (t, 1H, J = 7.6 Hz, C4-Ar), 7.51 (d, 1H, J = 7.6 Hz, C6-Ar), 7.62 (d, 1H, J = 7.6 Hz, C3-Ar), 7.72 (brs, 1H, NH-C=O), 8.16 (s, 1H, CH-pyridazine); ^{13}C NMR ($CDCl_3$, 100 MHz) *δ ppm*: 27.75 ($3xCH_3$), 41.66 (CH_2-NH), 50.42 (NH-C-t-butyl), 113.13 (q, $J_{(C,F)}$ = 33.6 Hz, C5), 120.28 (q, $J_{(C,F)}$ = 271.8 Hz, CF_3-pyridazine), 122.05 (q, $J_{(C,F)}$ = 272.1 Hz, CF_3-Ar), 122.42 (q, $J_{(C,F)}$ = 4.8 Hz, C4), 125.37 (q, $J_{(C,F)}$ = 5.7 Hz, C3-Ar), 127.26 (q, $J_{(C,F)}$ = 30.5 Hz, C2-Ar), 126.97 (C6-Ar), 129.06 (C4-Ar), 131.34 (C5-Ar), 134.58 (C1-Ar), 145.26 (C=N), 153.06 (C=O), 160.15 (-N-C=N); Analysis for $C_{18}H_{18}F_6N_4O$ (420), Calcd.: C, 51.43; H, 4.32; N, 13.33; Found: C, 51.50; H, 4.29; N, 13.31.

N-Butyl-6-morpholino-5-(trifluoromethyl)pyridazine-3-carboxamide (11i)

Brown oil; Yield (69%); ^{1}H NMR ($CDCl_3$, 400 MHz) *δ ppm*: 0.86 (t, 3H, J = 7.2 Hz, CH_3), 1.31-1.40 (m, 2H, CH_2), 1.51-1.59 (m, 2H, CH_2), 3.41 (q, 2H, J = 6.8Hz, CH_2-NH), 3.57 (t, 4H, J = 4.8 Hz, $2xCH_2$-N), 3.79 (t, 4H, J = 4.8 Hz, $2xCH_2$-O), 7.83 (t-like, J = 4.0 Hz, 1H, NH), 8.27 (s, 1H, CH-pyridazine); ^{13}C NMR ($CDCl_3$, 100 MHz) *δ ppm*: 12.09 (CH_3), 19.03, 30.53 (CH_2), 38.30 (CH_2-NH), 49.26 ($2xCH_2$-N), 65.56 ($2xCH_2$-O), 117.66 (q, $J_{(C,F)}$ = 34.2 Hz, C5), 120.35 (q, $J_{(C,F)}$ = 272.5 Hz, CF_3), 124.93 (q, $J_{(C,F)}$ = 5.2 Hz, C4), 146.14, 157.46 (C=O), 160.73 (-N-C=N); Analysis for $C_{14}H_{19}F_3N_4O_2$ (332), Calcd.: C, 50.60; H, 5.76; N, 16.86; Found: C, 50.77; H, 5.78; N, 16.93.

6-((2-Hydroxyethyl)amino)-N-((tetrahydro-2H-pyran-4-yl)methyl)-5-(trifluoro methyl)pyridazine-3-carboxamide (11j)

White solid; Yield (79%); m.p. 95-96 °C; ^{1}H NMR ($CDCl_3$, 400 MHz) *δ ppm*: 1.24-1.37 (m, 2H), 1.59 (d, 2H, J = 12.8 Hz), 1.76-1.82 (m, 1H, CH), 3.27-3.34 (m, 4H, $2xCH_2$-O), 3.83 (t, 2H, J = 4.4 Hz, CH_2-NH), 3.87 (t, 2H, J = 4.8 Hz, CH_2-OH), 3.91 (d, 2H, J = 3.2 Hz), 5.74 (brs, 1H, NH), 7.89 (t-like, 1H, J = 4.4 Hz, NH-C=O), 8.12 (s, 1H, CH-pyridazine); ^{13}C NMR ($CDCl_3$, 100 MHz) *δ ppm*: 29.57 ($2xCH_2$), 34.47 (CH), 43.44, 44.10

(CH_2-NH), 60.12 (CH_2-O), 66.54 (2xCH_2-O), 113.16 (q, $J_{(C,F)}$ = 33.8 Hz, C5), 120.23 (q, $J_{(C,F)}$ = 271.7 Hz, CF_3), 122.67 (q, $J_{(C,F)}$ = 5.1 Hz, C4), 143.82 (C=N), 153.90 (C=O), 161.43 (-N-C=N); Analysis for $C_{14}H_{19}F_3N_4O_3$ (348), Calcd.: C, 48.27; H, 5.50; N, 16.08; Found: C, 48.16; H, 5.49; N, 16.11.

6-(Butylamino)-N-((tetrahydro-2*H*-pyran-4-yl)methyl)-5-(trifluoromethyl)pyridazine-3-carboxamide (11k)

Yellow oil; Yield (48%); ^{1}H NMR ($CDCl_3$, 400 MHz) *δ ppm*: 0.89 (t, 3H, *J* = 7.2 Hz, CH_3), 1.26-1.43 (m, 4H), 1.59-1.69 (m, 4H), 1.75-1.83 (m, 1H, CH), 3.27 (m, 2H), 3.33 (t, 2H, *J* = 6.4 Hz), 3.64 (q, 2H, *J* = 6.8 Hz, CH_2-NH), 3.89 (dd, 2H, *J* = 3.2, 11.2 Hz, 2xCH_2-N), 5.17 (brs, 1H, NH), 7.89 (t-like, 1H, *J* = 5.2 Hz, NH-C=O), 8.11 (s, 1H, CH-pyridazine); ^{13}C NMR ($CDCl_3$, 100 MHz) *δ ppm*: 13.22 (CH_3), 29.57 (2xCH_2), 34.45, 34.51, 43.44, 44.07 (CH_2-NH), 46.34, 66.55 (2xCH_2-O), 112.50 (q, $J_{(C,F)}$ = 33.5 Hz, C5), 120.37 (q, $J_{(C,F)}$ = 271.7 Hz, CF_3), 122.44 (q, $J_{(C,F)}$ = 4.9 Hz, C4), 143.55 (C=N), 153.46 (C=O), 161.49 (-N-C=N); Analysis for $C_{16}H_{23}F_3N_4O_2$ (360), Calcd.: C, 53.33; H, 6.43; N, 15.55; Found: C, 53.48; H, 6.44; N, 15.59.

6-((Cyclopropylmethyl)amino)-N-((tetrahydro-2*H*-pyran-4-yl)methyl)-5-(trifluoromethyl) pyridazine-3-carboxamide (11l)

Yellow oil; Yield (38%); ^{1}H NMR ($CDCl_3$, 400 MHz) *δ ppm*: 0.25 (q-like, 2H, *J* = 4.8 Hz), 0.53 (q-like, 2H, *J* = 5.6 Hz), 1.10-1.17 (m, 1H), 1.26-1.37 (m, 2H,), 1.59 (d, 2H, *J* = 12.8 Hz), 1.76-1.80 (m, 1H), 3.27-3.35 (m, 4H, 2xCH_2-THP), 3.49 (dd, 2H, *J* = 4.8, 6.8 Hz, CH_2-NH), 3.85 (dd, 2H, *J* = 2.8, 11.2 Hz), 5.30 (brs, 1H, NH), 7.90 (t-like, 1H, *J* = 6.4 Hz, NH-C=O), 8.11 (s, 1H, CH-pyridazine); ^{13}C NMR ($CDCl_3$, 100 MHz) *δ ppm*: 12.74, 13.22, 19.06, 29.60 (2xCH_2), 30.02, 34.51 (CH), 41.12 (CH_2-NHCO), 44.07 (CH_2-NH), 66.55 (2xCH_2-O), 112.53 (q, $J_{(C,F)}$ = 33.0 Hz, C5), 120.23 (q, $J_{(C,F)}$ = 271.6 Hz, CF_3), 122.67 (q, $J_{(C,F)}$ = 5.0 Hz, C4), 143.45 (C=N), 153.59 (C=O), 161.49 (-N-C=N); Analysis for $C_{16}H_{21}F_3N_4O_2$ (358), Calcd.: C, 53.63; H, 5.91; N, 15.63; Found: C, 53.71; H, 5.86; N, 15.69.

6-Morpholino-N-((tetrahydro-2H-pyran-4-yl)methyl)-5-(trifluoromethyl)pyridazine-3-carboxamide (11m)

Light Yellow solid; Yield (73%); m.p. 67-68 °C; ^{1}H NMR ($CDCl_3$, 400 MHz) δ *ppm*: 1.27-1.38 (m, 2H), 1.60 (d, 2H, *J* = 11.6 Hz), 1.77-1.85 (m, 1H, CH), 3.27-3.37 (m, 2H), 3.33 (t, 2H, *J* = 6.4 Hz), 3.58 (t, 4H, *J* = 4.4 Hz, 2xCH_2-N), 3.79 (t, 4H, *J* = 4.8Hz, 2xCH_2-O), 3.89 (dd, 2H, *J* = 3.6 Hz, CH_2-NH), 7.94 (t-like, 1H, *J* = 5.6 Hz, NH-C=O), 8.26 (s, 1H, CH-pyridazine); ^{13}C NMR ($CDCl_3$, 100 MHz) δ *ppm*: 29.59 (2xCH_2), 34.49 (CH), 44.17 (CH_2-NH) 49.24 (2xCH_2-N), 65.54 (2xCH_2-O), 66.52 (2xCH_2-O, THP), 117.55 (q, $J_{(C,F)}$ = 33.9 Hz, C5), 120.32 (q, $J_{(C,F)}$ = 272.4 Hz, CF_3), 124.98 (q, $J_{(C,F)}$ = 5.3 Hz, C4), 145.86 (C=N), 157.46 (C=O), 160.99 (-N-C=N); Analysis for $C_{16}H_{21}F_3N_4O_3$ (374), Calcd.: C, 51.33; H, 5.65; N, 14.97; Found: C, 51.46; H, 5.68; N, 14.95.

6-((2-Hydroxyethyl)amino)-5-(trifluoromethyl)-N-(2-(trifluoromethyl) benzyl)pyridazine-3-carboxamide (11n)

Yellow oil; Yield (57%); ^{1}H NMR ($CDCl_3$, 400 MHz) δ *ppm*: 3.80-3.84 (m, 4H, 2xCH_2), 4.77 (d, 2H, *J* = 6.0 Hz, CH_2-NH), 5.76 (brs, 1H, NH), 7.29 (t, 1H, *J* = 7.6 Hz, C5-Ar), 7.41 (t, 1H, *J* = 7.6 Hz, C4-Ar), 7.50 (d, 1H, *J* = 7.6 Hz, C6-Ar), 7.57 (d, 1H, *J* = 7.6 Hz, C3-Ar), 8.13 (s, 1H, CH-pyridazine), 8.16 (t-like, 1H, *J* = 5.2 Hz, NH-CO); Analysis for $C_{16}H_{14}F_6N_4O_2$ (408), Calcd.: C, 47.07; H, 3.46; N, 13.72; Found: C, 46.92; H, 3.44; N, 13.76.

6-(Butylamino)-5-(trifluoromethyl)-N-(2-(trifluoromethyl)benzyl)pyridazine-3-carboxamide (11o)

Yellow oil; Yield (60%); ^{1}H NMR ($CDCl_3$, 400 MHz) δ*ppm*: 0.87 (t, 3H, *J* = 7.6 Hz, CH_3), 1.33-1.41 (m, 2H, CH_2), 1.59-1.67 (m, 2H, CH_2), 3.62 (q, 2H, *J* = 7.2 Hz, CH_2-NH), 4.78 (d, 2H, *J* = 6.4 Hz, CH_2), 5.17 (brs, 1H, NH), 7.29 (t, 1H, *J* = 7.6 Hz, C5-Ar), 7.42 (t, 1H, *J* = 7.6 Hz, C4-Ar), 7.52 (d, 1H, *J* = 8.0 Hz, C6-Ar), 7.58 (d, 1H, *J* = 8.0 Hz, C3-Ar), 8.12 (s, 1H, CH-pyridazine), 8.14 (t-like, 1H, *J* = 5.6 Hz, NH-CO); Analysis for $C_{18}H_{18}F_6N_4O$ (420), Calcd.:C, 51.43; H, 4.32; N, 13.33; Found: C, 51.54; H, 4.30; N, 13.39.

6-((Cyclopropylmethyl)amino)-5-(trifluoromethyl)-N-(2-(trifluoromethyl)benzyl) pyridazine-3-carboxamide (11p)

Yellow oil; Yield (61%); ^{1}H NMR ($CDCl_3$, 400 MHz) *δ ppm*: 0.23 (q-like, 2H, *J* = 5.2 Hz, CH_2), 0.52 (q-like, 2H, *J* = 5.2 Hz, CH_2), 1.09-1.15 (m, 1H, CH), 3.47 (dd, 2H, *J* = 5.6, 6.4 Hz, CH_2), 4.78 (d, 2H, *J* = 6.4 Hz, CH_2), 5.28 (brs, 1H, NH), 7.29 (t, 1H, *J* = 7.6 Hz, C5-Ar), 7.42 (t, 1H, *J* = 7.6 Hz, C4-Ar), 7.52 (d, 1H, *J* = 7.6 Hz, C6-Ar), 7.58 (d, 1H, *J* = 7.6 Hz, C3-Ar), 8.13 (s, 1H, CH-pyridazine), 8.16 (t-like, 1H, *J* = 5.6 Hz, NH-CO); Analysis for $C_{18}H_{16}F_6N_4O$ (418), Calcd.: C, 51.68; H, 3.86; N, 13.39; Found: C, 51.59; H, 3.87; N, 13.41.

6-(Cyclopentylamino)-5-(trifluoromethyl)-N-(2-(trifluoromethyl)benzyl)pyridazine-3-carboxamide (11q)

Yellow oil; Yield (51%); ^{1}H NMR (DMSO-d_6, 400 MHz) *δ ppm*: 1.42-1.50 (m, 2H, CH_2), 1.58-1.71 (m, 4H, 2xCH_2), 2.09-2.16 (m, 2H, CH_2), 4.77 (d, 2H, *J* = 6.4 Hz, CH_2-NH), 5.08 (d, 1H, *J* = 5.6 Hz, NH), 7.29 (t, 1H, *J* = 7.6 Hz, C5-Ar), 7.42 (t, 1H, *J* = 7.6 Hz, C4-Ar), 7.52 (d, 1H, *J* = 7.6 Hz, C6-Ar), 7.58 (d, 1H, *J* = 7.6 Hz, C3-Ar), 8.12 (s, 1H, CH-pyridazine), 8.14 (t-like, 1H, *J* = 5.6 Hz, NH-CO); Analysis for $C_{19}H_{18}F_6N_4O$ (432), Calcd.: C, 52.78; H, 4.20; N, 12.96; Found: C, 52.90; H, 4.16; N, 12.94.

6-Morpholino-5-(trifluoromethyl)-N-(2-(trifluoromethyl)benzyl)pyridazine-3-carboxamide (11r)

Yellow oil; Yield (60%); ^{1}H NMR ($CDCl_3$, 400 MHz) *δ ppm*: 3.65 (t, 4H, *J* = 4.4 Hz, 2xCH_2-N), 3.85 (t, 4H, *J* = 4.8 Hz, 2xCH_2-O), 4.87 (d, 2H, *J* = 6 Hz, CH_2-NH), 7.38 (t, 1H, *J* = 7.6 Hz, C5-Ar), 7.50 (t, 1H, *J* = 8.0 Hz, C4-Ar), 7.61 (d, 1H, *J* = 7.6 Hz, C6-Ar), 7.67 (d, 1H, *J* = 8.0 Hz, C3-Ar), 8.26 (t-like, 1H, *J* = 5.2 Hz, NH-CO), 8.35 (s, 1H, CH-pyridazine); Analysis for $C_{18}H_{16}F_6N_4O_2$ (434), Calcd.: C, 49.78; H, 3.71; N, 12.90; Found: C, 49.84; H, 3.72; N, 12.87.

2. Anti-proliferative activities

The three examined human cancer cell lines; T-47D (breast cancer), MDA-MB-231 (breast carcinoma) and SKOV-3 (ovarian cancer), as well as the non-tumorigenic human breast cell line (MCF-10A) have been obtained from American Type Culture Collection (ATCC). The cells were maintained in Dulbecco's modified Eagle's medium (DMEM) supplemented with 10% heat inactivated fetal calf serum (GIBCO), penicillin (100 U/ml) and streptomycin (100 µg/ml) at 37 ^{o}C in humidified atmosphere containing 5% CO_2. Cells at a concentration of 0.50 x 10^6 were grown in a 25 cm^2 flask in 5 ml of culture medium.

The anti-proliferative activity of the tested pyridazines **11a-r** was measured *in vitro* using the Sulfo-Rhodamine-B stain (SRB) assay. Briefly, Cells were inoculated in 96-well microtiter plate (5X10^4 cells/ well) for 24 h before treatment with the tested pyridazines to allow attachment of cell to the wall of the plate. Tested pyridazines were dissolved in DMSO at 1 mg/ml immediately before use and diluted to the appropriate volume just before addition to the cell culture. Different concentrations of tested pyridazines and staurosporine were added to the cells (three wells were prepared for each individual dose). Cells were incubated with the pyridazines for 48 h at 37°C and in atmosphere of 5% CO_2. After 48 h cells were fixed, washed, and stained for 30 min with 0.4% (w/v) SRB dissolved in 1% acetic acid. Unbound dye was removed by four washes with 1% acetic acid, and attached stain was recovered with Tris-EDTA buffer. Color intensity was measured in an ELISA reader. The relation between percent of surviving fraction and drug concentration is plotted to get the survival curve for each cell line. The concentration required for 50% inhibition of cell viability (IC_{50}) was calculated.

3. Cell Cycle Analysis

Breast cancer T-47D and MDA-MB-231 cells were treated with pyridazines **11l** and **11m** for 24 h (at their IC_{50} concentration), and then cells were washed twice with ice-cold phosphate buffered saline (PBS). Subsequently, the treated cells were collected by centrifugation, fixed in ice-cold 70% (*v*/*v*) ethanol, washed with PBS, re-suspended with 100 µg/mL RNase, stained with 40 µg/mL PI, and analyzed by flow cytometry using FACS Calibur (Becton Dickinson, BD, Franklin Lakes, NJ, USA). The cell cycle distributions were calculated using CellQuest software 5.1 (Becton Dickinson).

4. Annexin V-FITC Apoptosis Assay

Phosphatidylserine externalization was assayed using Annexin V-FITC/PI apoptosis detection kit (BD Biosciences, USA) according to the manufacturer's instructions. Breast cancer T-47D and MDA-MB-231 cells were cultured to a monolayer then treated with pyridazines **11l** and **11m** at their IC_{50} concentration. Briefly, cells were then harvested *via* trypsinization, and rinsed twice in PBS followed by binding buffer. Moreover, cells were re-suspended in 100 µL of binding buffer with the addition of 1 µL of FITC-Annexin V followed by an incubation period of 30 min at 4 °C. Cells were then rinsed in binding buffer and resuspended in 150 µL of binding buffer with the addition of 1 µL of DAPI (1 µg/µL in PBS). Cells were then analyzed using the flow cytometer BD FACS Canto II and the results were interpreted with FlowJo7.6.4 software (Tree Star, Ashland, OR, USA).

5. CDK Kinase Inhibitory Activity

The CDK2 enzyme inhibitory activity was determined for pyridazines **11e**, **11h**, **11l**, and **11m** using the CDK2/CyclinA2 Kinase Enzyme System (catalog No. V2971) (Promega, Milan, Italy), according to the manufacturer's instructions.

6. Statistical Analysis

Data are presented as means ± S.D. Individual groups were compared using the two-tailed independent Student's *t*-test. Multiple group comparisons were carried out using one-way analysis of variance (ANOVA) followed by the Tukey–Kramer test for post-hoc analysis. Statistical significance was accepted at a level of $p < 0.05$. All statistical analyses were performed using GraphPad InStat software, version 3.05 (GraphPad Software, Inc., La Jolla, CA, USA). Graphs were sketched using GraphPad Prism software, version 5.00 (GraphPad Software, Inc., La Jolla, CA, USA).

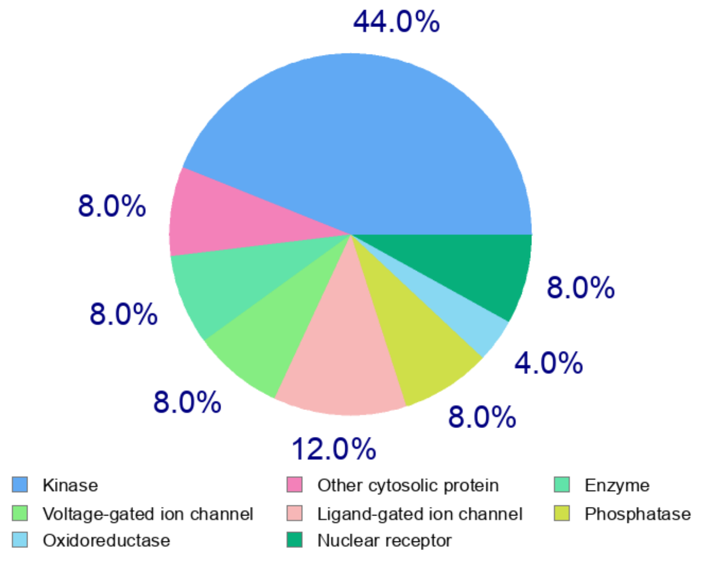

Figure S1. Different target classes suggested by SwissTargetPrediction online tool (for compound 5J), showing the protein kinases as the most probable targets

SwissTargetPrediction

Target	Common name	Uniprot ID	ChEMBL ID	Target Class	Probability*	Known actives (3D/2D)
Pyruvate dehydrogenase kinase isoform 2	PDK2	Q15119	CHEMBL3861	Kinase	0.104671941128	131 / 0
MAP kinase signal-integrating kinase 2	MKNK2	Q9HBH9	CHEMBL4204	Kinase	0.104671941128	1 / 0
Tyrosine-protein kinase JAK1	JAK1	P23458	CHEMBL2835	Kinase	0.104671941128	78 / 0
Tyrosine-protein kinase JAK2	JAK2	O60674	CHEMBL2971	Kinase	0.104671941128	87 / 0
Leucine-rich repeat serine/threonine-protein kinase 2	LRRK2	Q5S007	CHEMBL1075104	Kinase	0.104671941128	88 / 0
Cyclin-dependent kinase 1/cyclin B	CCNB3<br>CDK1<br>CCNB1<br>CCNB2	Q8WWL7<br>P06493<br>P14635<br>O95067	CHEMBL2094127	Other cytosolic protein	0.104671941128	4 / 0
Cyclin-dependent kinase 2/cyclin A	CDK2<br>CCNA1<br>CCNA2	P24941<br>P78396<br>P20248	CHEMBL2094128	Other cytosolic protein	0.104671941128	1 / 0
Acyl-CoA desaturase	SCD	O00767	CHEMBL5555	Enzyme	0.104671941128	19 / 110
Tyrosine-protein kinase SRC	SRC	P12931	CHEMBL267	Kinase	0.104671941128	10 / 0
Tyrosine-protein kinase ZAP-70	ZAP70	P43403	CHEMBL2803	Kinase	0.104671941128	6 / 0
Sodium channel protein type X alpha subunit (by homology)	SCN10A	Q9Y5Y9	CHEMBL5451	Voltage-gated ion channel	0.104671941128	4 / 0
Glutamate NMDA receptor; GRIN1/ GRIN2A	GRIN2A<br>GRIN1	Q12879<br>Q05586	CHEMBL1907604	Ligand-gated ion channel	0.104671941128	3 / 0
Glutamate receptor ionotropic, AMPA 2	GRIA2	P42262	CHEMBL4016	Ligand-gated ion channel	0.104671941128	19 / 0
PI3-kinase p110-alpha subunit	PIK3CA	P42336	CHEMBL4005	Enzyme	0.104671941128	51 / 0
Protein-tyrosine phosphatase 1B	PTPN1	P18031	CHEMBL335	Phosphatase	0.104671941128	27 / 0
T-cell protein-tyrosine phosphatase	PTPN2	P17706	CHEMBL3807	Phosphatase	0.104671941128	16 / 0
Serine/threonine-protein kinase AKT	AKT1	P31749	CHEMBL4282	Kinase	0.104671941128	21 / 0
HERG	KCNH2	Q12809	CHEMBL240	Voltage-gated ion channel	0.104671941128	87 / 0
Inhibitor of nuclear factor kappa B kinase epsilon subunit	IKBKE	Q14164	CHEMBL3529	Kinase	0.104671941128	1 / 0
Arachidonate 5-lipoxygenase	ALOX5	P09917	CHEMBL215	Oxidoreductase	0.104671941128	6 / 0
LXR-alpha	NR1H3	Q13133	CHEMBL2808	Nuclear receptor	0.104671941128	66 / 0
LXR-beta	NR1H2	P55055	CHEMBL4093	Nuclear receptor	0.104671941128	78 / 0

Target	Common name	Uniprot ID	ChEMBL ID	Target Class	Probability*	Known actives (3D/2D)
Cyclin-dependent kinase 2	CDK2	P24941	CHEMBL301	Kinase	0.104671941128	18 / 0
Cyclin-dependent kinase 1	CDK1	P06493	CHEMBL308	Kinase	0.104671941128	15 / 0
P2X purinoceptor 7	P2RX7	Q99572	CHEMBL4805	Ligand-gated ion channel	0.104671941128	21 / 0
GABA receptor alpha-5 subunit	GABRA5	P31644	CHEMBL5112	Ligand-gated ion channel	0.104671941128	20 / 0
MAP kinase ERK2	MAPK1	P28482	CHEMBL4040	Kinase	0.104671941128	9 / 0
Microtubule-associated protein 2	MAP2	P11137	CHEMBL2390810	Unclassified protein	0.104671941128	10 / 0
Cathepsin K	CTSK	P43235	CHEMBL268	Protease	0.104671941128	15 / 0
Cathepsin S	CTSS	P25774	CHEMBL2954	Protease	0.104671941128	12 / 0
Cholesteryl ester transfer protein	CETP	P11597	CHEMBL3572	Other ion channel	0.104671941128	61 / 0
11-beta-hydroxysteroid dehydrogenase 1	HSD11B1	P28845	CHEMBL4235	Enzyme	0.104671941128	88 / 0
Sodium channel protein type IX alpha subunit	SCN9A	Q15858	CHEMBL4296	Voltage-gated ion channel	0.104671941128	66 / 0
Hormone sensitive lipase	LIPE	Q05469	CHEMBL3590	Enzyme	0.104671941128	25 / 0
Vanilloid receptor	TRPV1	Q8NER1	CHEMBL4794	Voltage-gated ion channel	0.104671941128	268 / 0
Fibroblast growth factor receptor 3	FGFR3	P22607	CHEMBL2742	Kinase	0.104671941128	32 / 0
15-hydroxyprostaglandin dehydrogenase [NAD+]	HPGD	P15428	CHEMBL1293255	Enzyme	0.104671941128	3 / 0
ADAM17	ADAM17	P78536	CHEMBL3706	Protease	0.104671941128	7 / 0
Signal transducer and activator of transcription 3	STAT3	P40763	CHEMBL4026	Transcription factor	0.104671941128	1 / 0
Dihydroorotate dehydrogenase	DHODH	Q02127	CHEMBL1966	Oxidoreductase	0.104671941128	7 / 0
Beta-glucocerebrosidase	GBA	P04062	CHEMBL2179	Enzyme	0.104671941128	1 / 0
Epoxide hydratase	EPHX2	P34913	CHEMBL2409	Protease	0.104671941128	29 / 0
Sodium channel protein type V alpha subunit	SCN5A	Q14524	CHEMBL1980	Voltage-gated ion channel	0.0	5 / 0
Tyrosine kinase non-receptor protein 2	TNK2	Q07912	CHEMBL4599	Kinase	0.0	1 / 0
5-lipoxygenase activating protein	ALOX5AP	P20292	CHEMBL4550	Other cytosolic protein	0.0	16 / 0
Beta-secretase 1	BACE1	P56817	CHEMBL4822	Protease	0.0	78 / 0
Diacylglycerol O-acyltransferase 1	DGAT1	O75907	CHEMBL6009	Enzyme	0.0	14 / 0
Thrombin and coagulation factor X	F10	P00742	CHEMBL244	Protease	0.0	97 / 0

Target	Common name	Uniprot ID	ChEMBL ID	Target Class	Probability*	Known actives (3D/2D)
Probable G-protein coupled receptor 52	GPR52	Q9Y2T5	CHEMBL3297639	Family A G protein-coupled receptor	0.0	11 / 0
Cathepsin L	CTSL	P07711	CHEMBL3837	Protease	0.0	1 / 0
Phosphodiesterase 5A	PDE5A	O76074	CHEMBL1827	Phosphodiesterase	0.0	21 / 0
Mitogen-activated protein kinase kinase kinase 12	MAP3K12	Q12852	CHEMBL1908389	Enzyme	0.0	62 / 0
Glucagon-like peptide 1 receptor	GLP1R	P43220	CHEMBL1784	Family B G protein-coupled receptor	0.0	3 / 0
Glucagon receptor	GCGR	P47871	CHEMBL1985	Family B G protein-coupled receptor	0.0	10 / 0
Glucocorticoid receptor	NR3C1	P04150	CHEMBL2034	Nuclear receptor	0.0	25 / 0
Fibroblast growth factor receptor 1	FGFR1	P11362	CHEMBL3650	Kinase	0.0	2 / 0
Monocarboxylate transporter 1	SLC16A1	P53985	CHEMBL4360	Electrochemical transporter	0.0	6 / 0
Gastric inhibitory polypeptide receptor	GIPR	P48546	CHEMBL4383	Family B G protein-coupled receptor	0.0	5 / 0
Anandamide amidohydrolase	FAAH	O00519	CHEMBL2243	Enzyme	0.0	19 / 0
Serine/threonine-protein kinase AKT2	AKT2	P31751	CHEMBL2431	Kinase	0.0	5 / 0
Serine/threonine-protein kinase AKT	AKT3	Q9Y243	CHEMBL4816	Kinase	0.0	5 / 0
Transient receptor potential cation channel subfamily M member 8	TRPM8	Q7Z2W7	CHEMBL1075319	Voltage-gated ion channel	0.0	26 / 0
Integrin alpha-4/ beta-1	ITGB1 ITGA4	P05556 P13612	CHEMBL1907599	Membrane receptor	0.0	9 / 0
Liver glycogen phosphorylase	PYGL	P06737	CHEMBL2568	Enzyme	0.0	3 / 0
Serine/threonine-protein kinase PIM1	PIM1	P11309	CHEMBL2147	Kinase	0.0	9 / 0
Peroxisome proliferator-activated receptor gamma	PPARG	P37231	CHEMBL235	Nuclear receptor	0.0	66 / 0
Peroxisome proliferator-activated receptor alpha	PPARA	Q07869	CHEMBL239	Nuclear receptor	0.0	17 / 0
Serine/threonine-protein kinase PIM2	PIM2	Q9P1W9	CHEMBL4523	Kinase	0.0	7 / 0
Phosphodiesterase 10A (by homology)	PDE10A	Q9Y233	CHEMBL4409	Phosphodiesterase	0.0	16 / 0
Receptor protein-tyrosine kinase erbB-2	ERBB2	P04626	CHEMBL1824	Kinase	0.0	15 / 0
Nerve growth factor receptor Trk-A	NTRK1	P04629	CHEMBL2815	Kinase	0.0	30 / 0
Neurokinin 1 receptor	TACR1	P25103	CHEMBL249	Family A G protein-coupled receptor	0.0	155 / 0

Target	Common name	Uniprot ID	ChEMBL ID	Target Class	Probability*	Known actives (3D/2D)
Tyrosine-protein kinase ABL (by homology)	ABL1	P00519	CHEMBL1862	Kinase	0.0	10 / 0
Cyclin T1	CCNT1	O60563	CHEMBL2108	Other cytosolic protein	0.0	6 / 0
Thymidine kinase, mitochondrial	TK2	O00142	CHEMBL4580	Enzyme	0.0	1 / 0
Serine/threonine-protein kinase B-raf	BRAF	P15056	CHEMBL5145	Kinase	0.0	43 / 0
Glucokinase regulatory protein	GCKR	Q14397	CHEMBL1075152	Enzyme	0.0	25 / 0
Histone deacetylase 3	HDAC3	O15379	CHEMBL1829	Eraser	0.0	2 / 0
Cyclin-dependent kinase 2/cyclin E1	CCNE1 CDK2	P24864 P24941	CHEMBL1907605	Kinase	0.0	3 / 0
CDK3/Cyclin E	CCNE1 CDK3	P24864 Q00526	CHEMBL3038471	Kinase	0.0	3 / 0
Inhibitor of nuclear factor kappa B kinase beta subunit	IKBKB	O14920	CHEMBL1991	Kinase	0.0	2 / 0
c-Jun N-terminal kinase 3	MAPK10	P53779	CHEMBL2637	Kinase	0.0	8 / 0
Inhibitor of NF-kappa-B kinase (IKK)	CHUK	O15111	CHEMBL3476	Kinase	0.0	2 / 0
2-acylglycerol O-acyltransferase 2	MOGAT2	Q3SYC2	CHEMBL2439944	Transferase	0.0	5 / 0
Kinesin-like protein 1	KIF11	P52732	CHEMBL4581	Other cytosolic protein	0.0	2 / 0
Sodium/glucose cotransporter 1	SLC5A1	P13866	CHEMBL4979	Electrochemical transporter	0.0	136 / 0
Hepatocyte growth factor receptor	MET	P08581	CHEMBL3717	Kinase	0.0	12 / 0
Focal adhesion kinase 1	PTK2	Q05397	CHEMBL2695	Kinase	0.0	21 / 0
Mitogen-activated protein kinase kinase kinase 14	MAP3K14	Q99558	CHEMBL5888	Kinase	0.0	22 / 0
Tyrosine-protein kinase JAK3	JAK3	P52333	CHEMBL2148	Kinase	0.0	11 / 0
Cannabinoid receptor 1 (by homology)	CNR1	P21554	CHEMBL218	Family A G protein-coupled receptor	0.0	17 / 0
Cannabinoid receptor 2	CNR2	P34972	CHEMBL253	Family A G protein-coupled receptor	0.0	27 / 0
PI3-kinase p110-delta subunit	PIK3CD	O00329	CHEMBL3130	Enzyme	0.0	29 / 0
PI3-kinase p110-beta subunit	PIK3CB	P42338	CHEMBL3145	Enzyme	0.0	27 / 0
Androgen Receptor (by homology)	AR	P10275	CHEMBL1871	Nuclear receptor	0.0	76 / 0
Metabotropic glutamate receptor 2 (by homology)	GRM2	Q14416	CHEMBL5137	Family C G protein-coupled receptor	0.0	8 / 0

Target	Common name	Uniprot ID	ChEMBL ID	Target Class	Probability*	Known actives (3D/2D)
Interleukin-8 receptor B	CXCR2	P25025	CHEMBL2434	Family A G protein-coupled receptor	0.0	2 / 0
Fructose-1,6-bisphosphatase	FBP1	P09467	CHEMBL3975	Enzyme	0.0	9 / 0
Interleukin-8 receptor A	CXCR1	P25024	CHEMBL4029	Family A G protein-coupled receptor	0.0	2 / 0
Vasopressin V2 receptor	AVPR2	P30518	CHEMBL1790	Family A G protein-coupled receptor	0.0	3 / 0

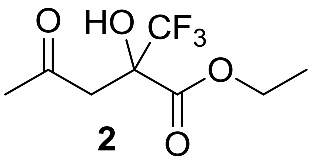
O
HO
CF3
O
O

2

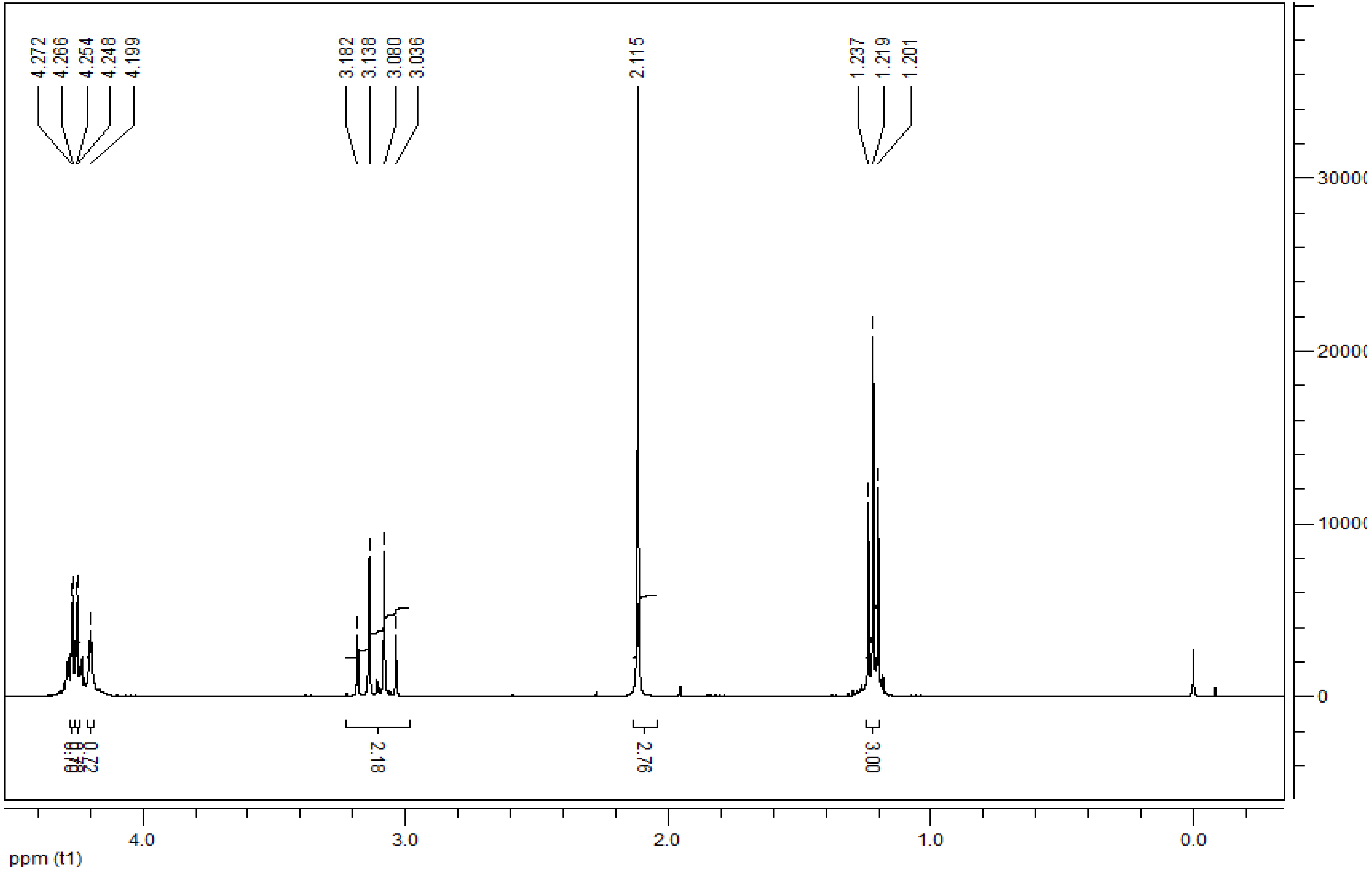
4.272
4.266
4.254
4.248
4.199
3.182
3.138
3.080
3.036
2.115
1.237
1.219
1.201
30000
20000
10000
0
0.72
2.18
2.76
3.00
4.0
3.0
2.0
1.0
0.0
ppm (t1)

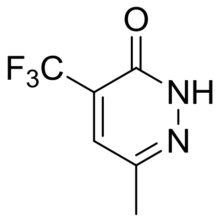

3

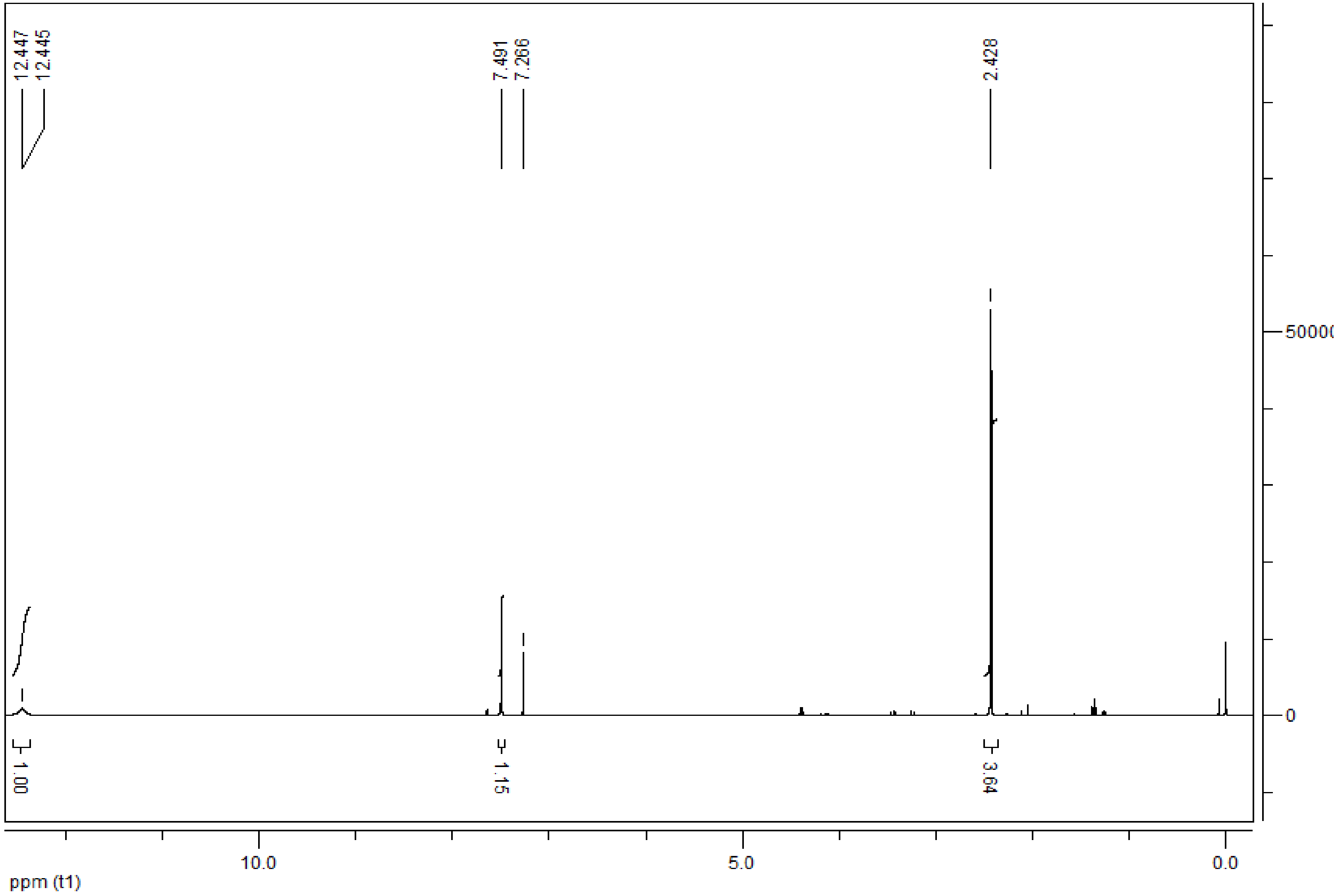

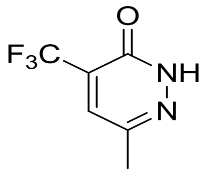

3

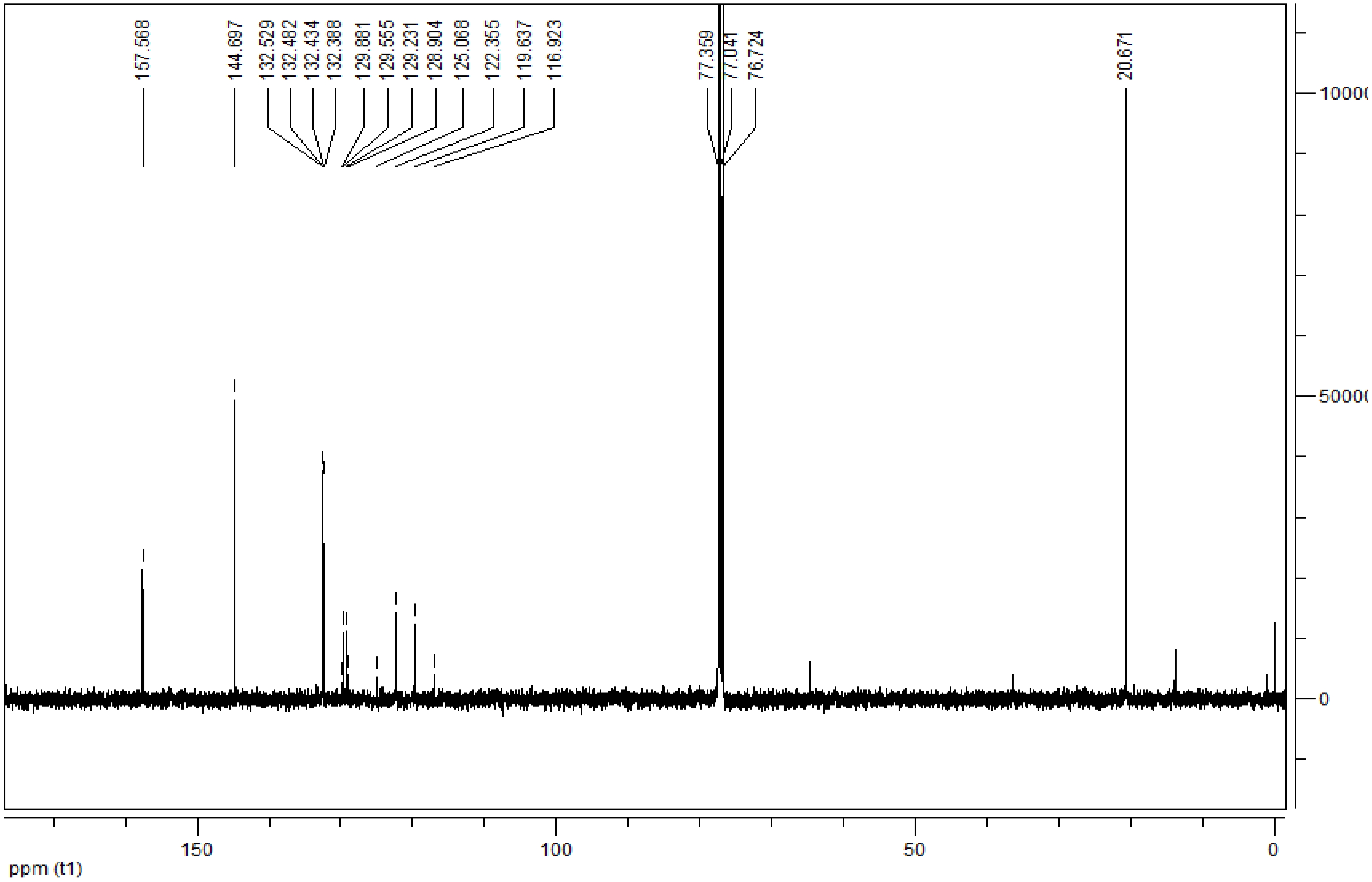

157.568
144.697
132.529
132.482
132.434
132.388
129.881
129.555
129.231
128.904
125.068
122.355
119.637
116.923
77.359
77.041
76.724
20.671
ppm (t1)

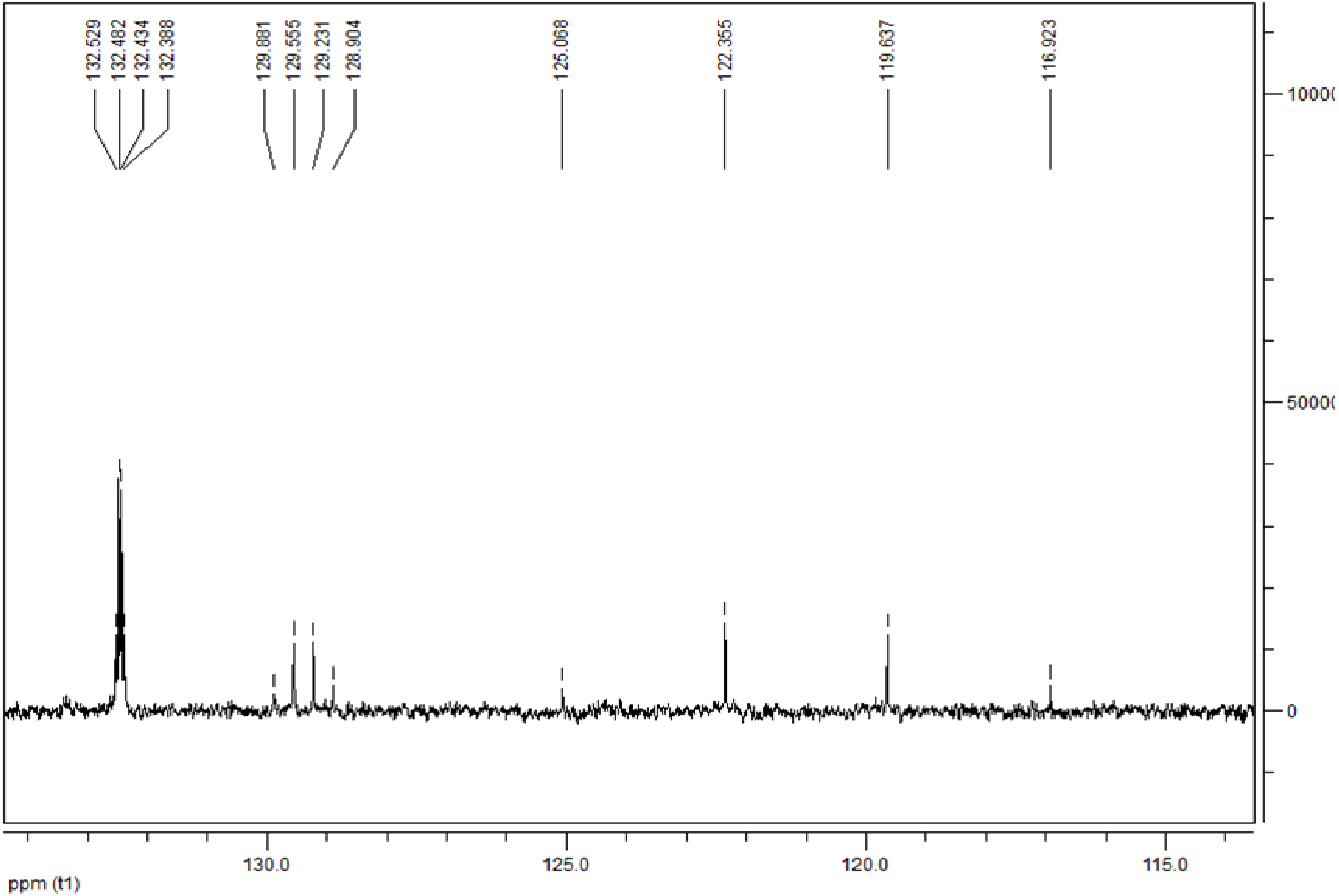

132.529
132.482
132.434
132.388
129.881
129.555
129.231
128.904
125.068
122.355
119.637
116.923
10000
50000
0
130.0
125.0
120.0
115.0
ppm (t1)

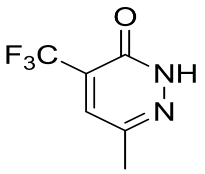

3

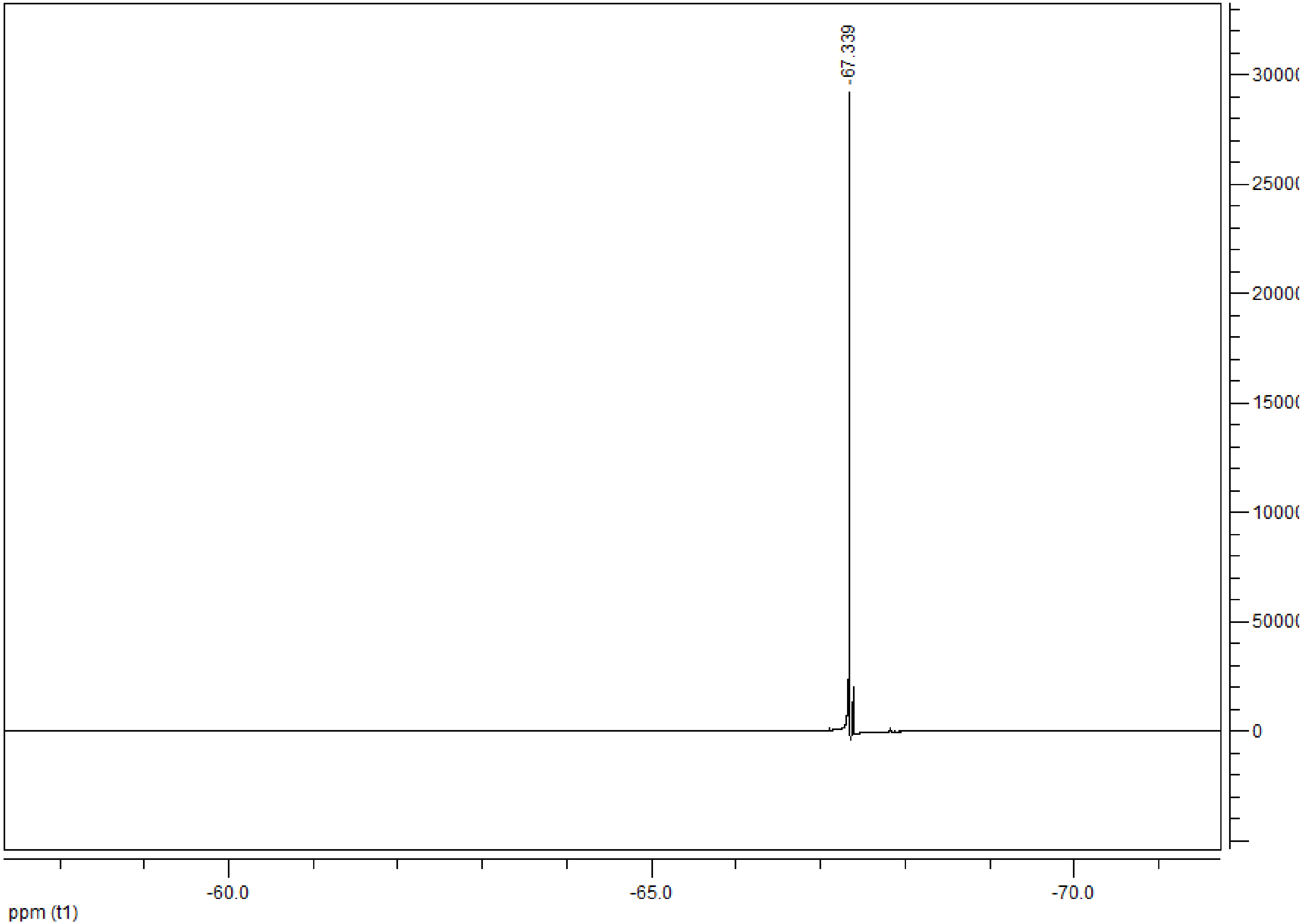

-67.339
30000
25000
20000
15000
10000
50000
0
-60.0
-65.0
-70.0
ppm (t1)

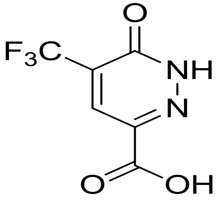
O
F3C
NH
N
O
OH

4

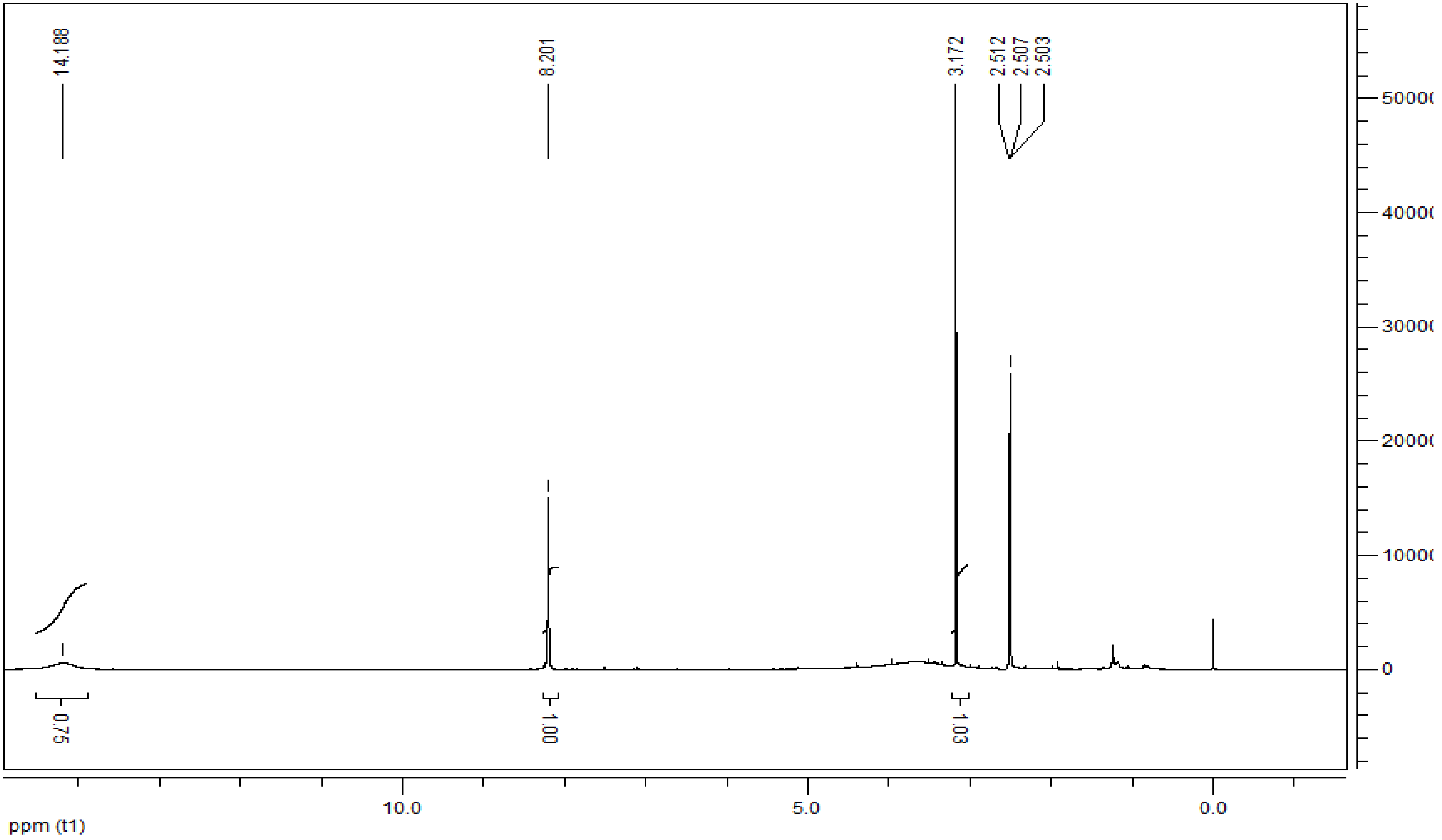
14.188
8.201
3.172
2.512
2.507
2.503
50000
40000
30000
20000
10000
0
0.75
1.00
1.03
10.0
5.0
0.0
ppm (t1)

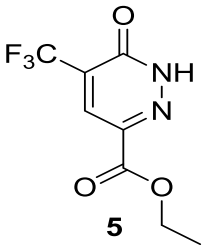

O
F3C
NH
N
O
O

5

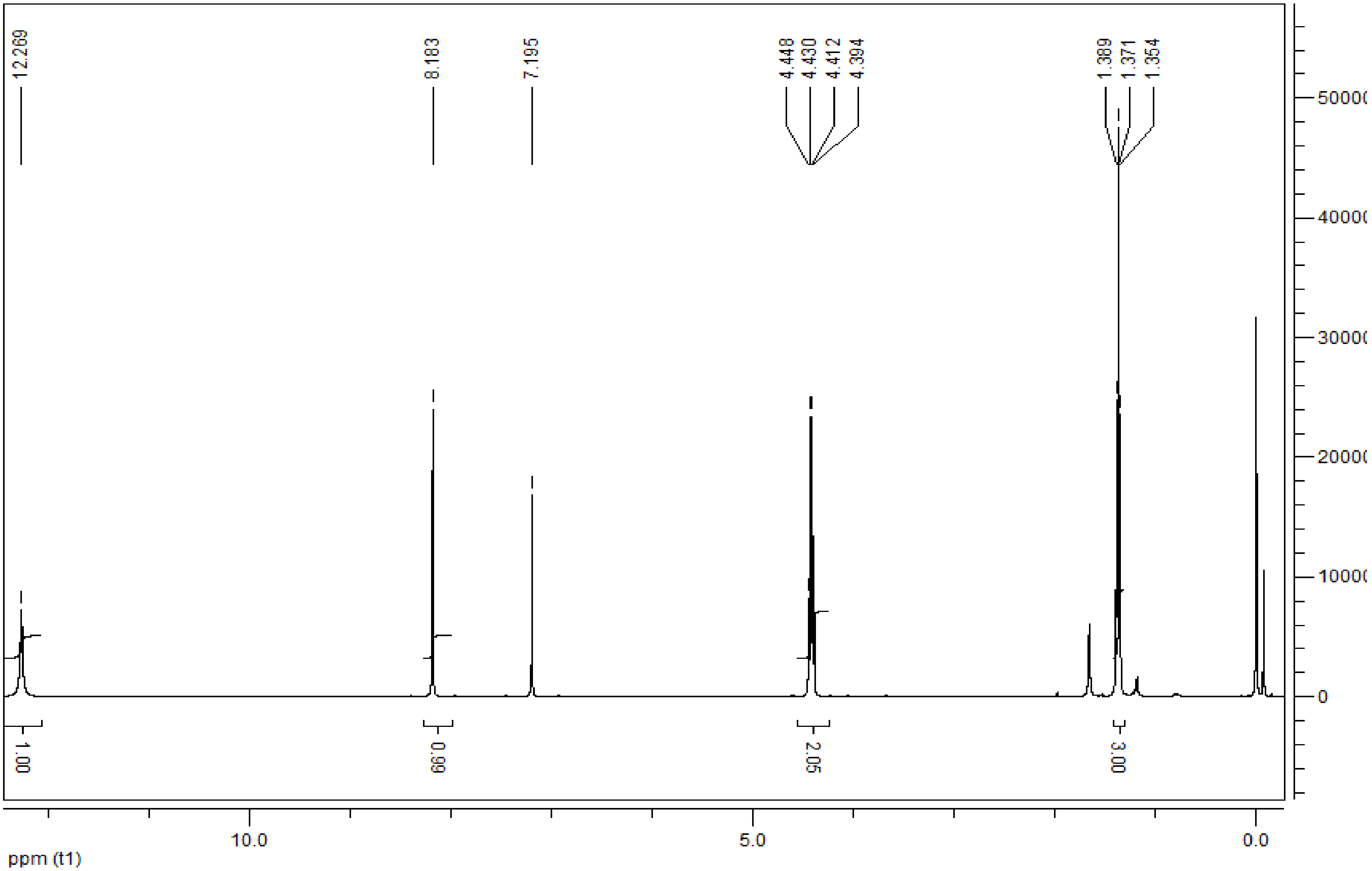

12.269
8.183
7.195
4.448
4.430
4.412
4.394
1.389
1.371
1.354
50000
40000
30000
20000
10000
0
1.00
0.99
2.05
3.00
10.0
5.0
0.0
ppm (t1)

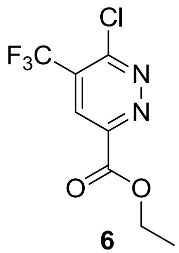
Cl
F3C
N
N
O
O

6

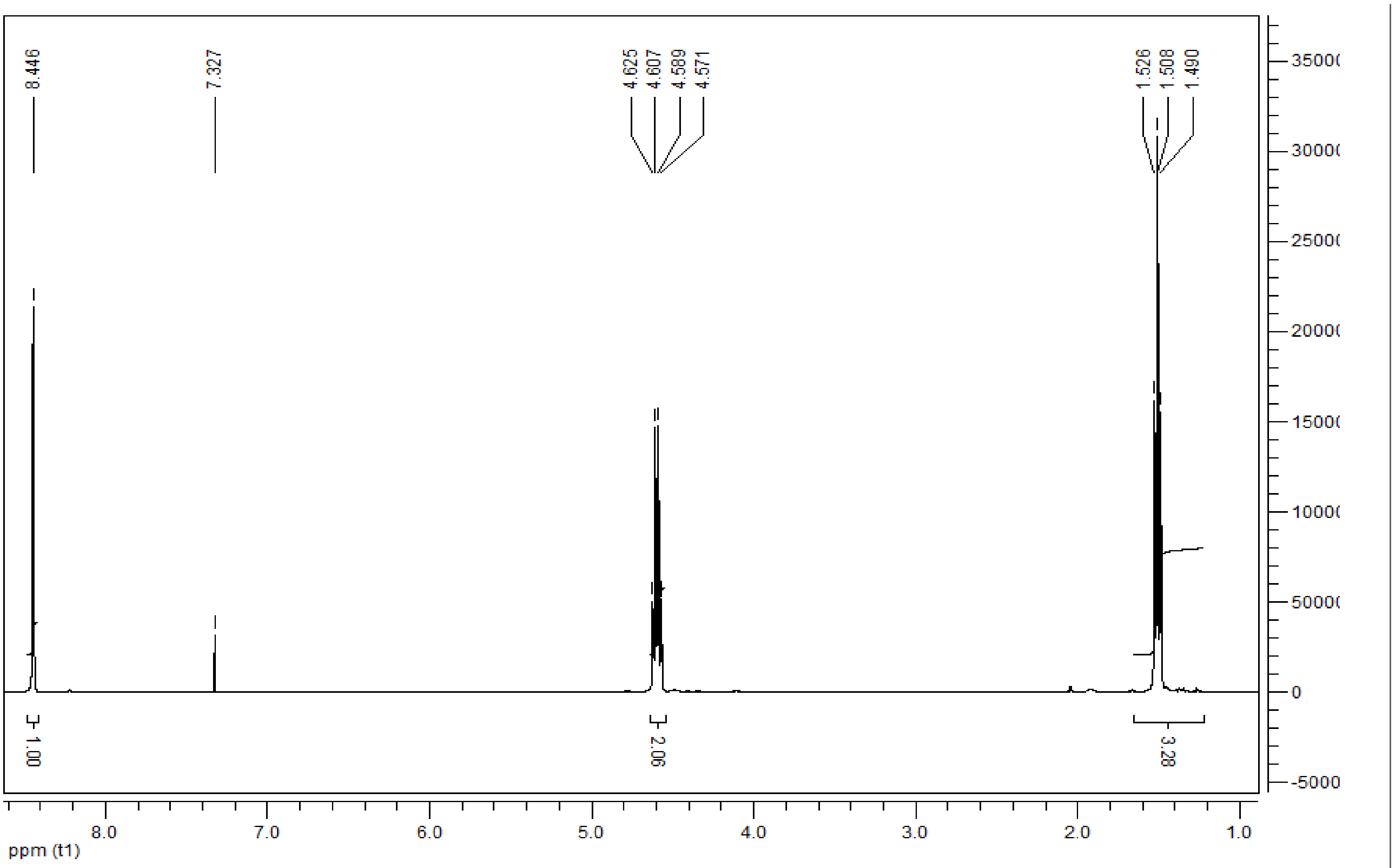
8.446
7.327
4.625
4.607
4.589
4.571
1.526
1.508
1.490
1.00
2.06
3.28
8.0
7.0
6.0
5.0
4.0
3.0
2.0
1.0
ppm (t1)
35000
30000
25000
20000
15000
10000
50000
0
-5000

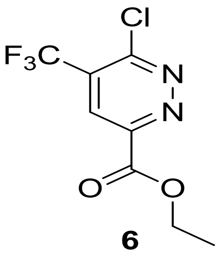

6

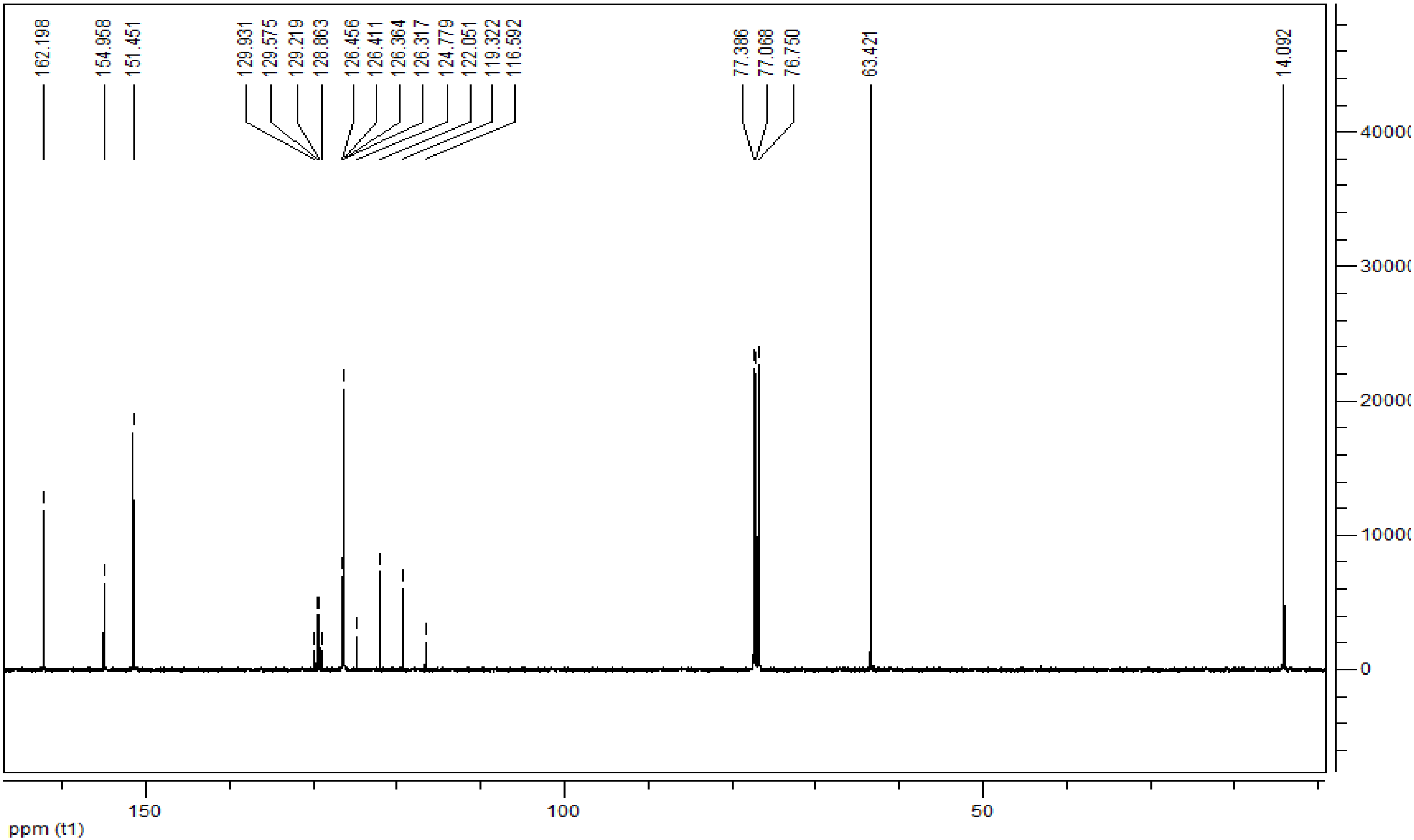
162.198
154.958
151.451
129.931
129.575
129.219
128.863
126.456
126.411
126.364
126.317
124.779
122.051
119.322
116.592
77.386
77.068
76.750
63.421
14.092
40000
30000
20000
10000
0
150
100
50
ppm (t1)

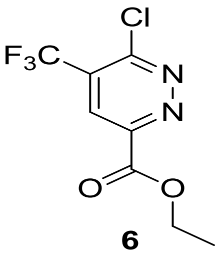
Cl
F3C
N
N
O
O

6

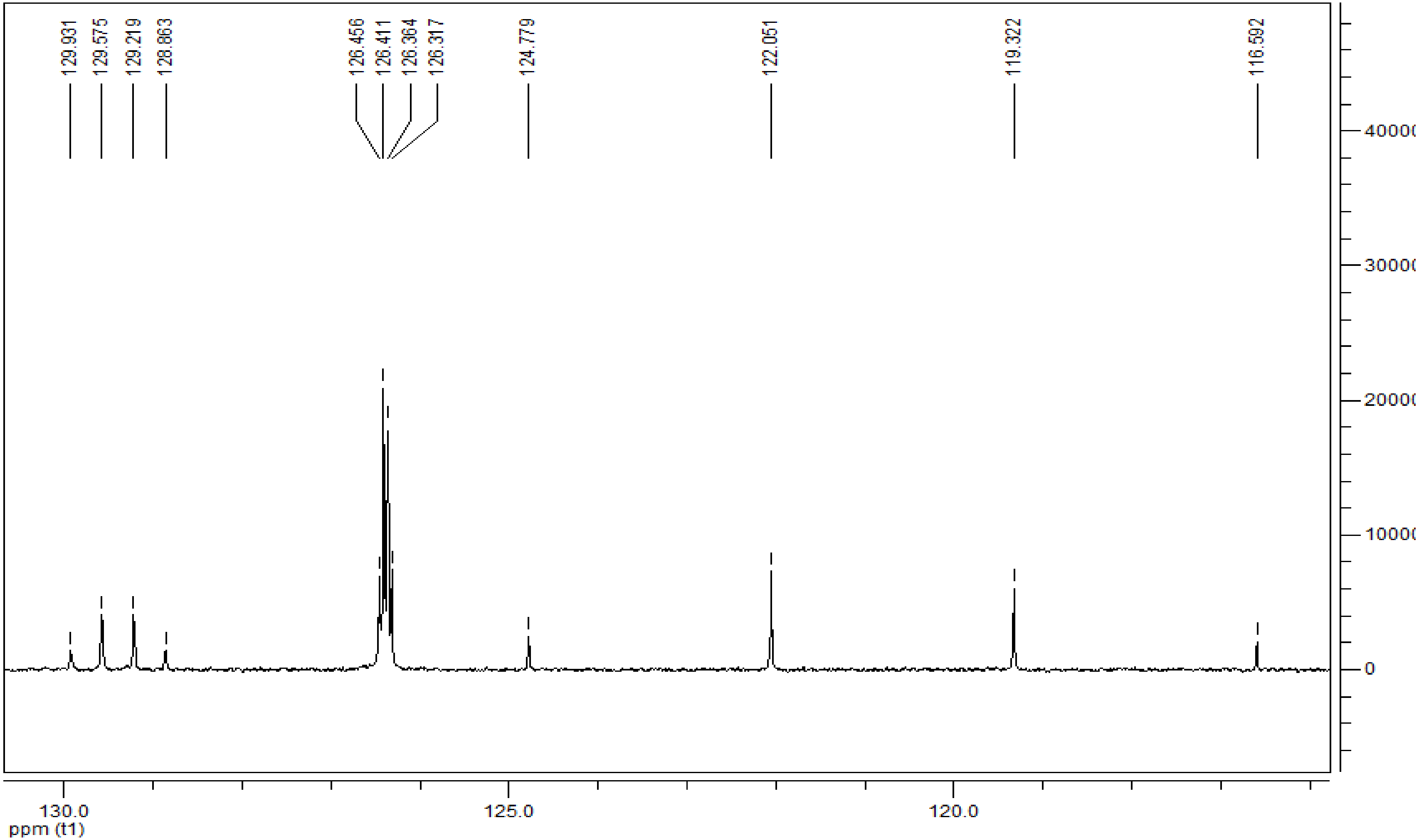
129.931
129.575
129.219
128.863
126.456
126.411
126.364
126.317
124.779
122.051
119.322
116.592
40000
30000
20000
10000
0
130.0
125.0
120.0
ppm (t1)

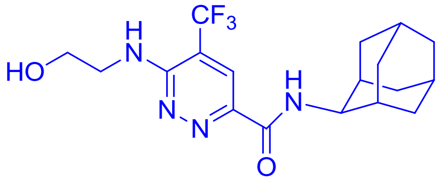

CF3
HO
H
N
N
N
H
N
O

11a

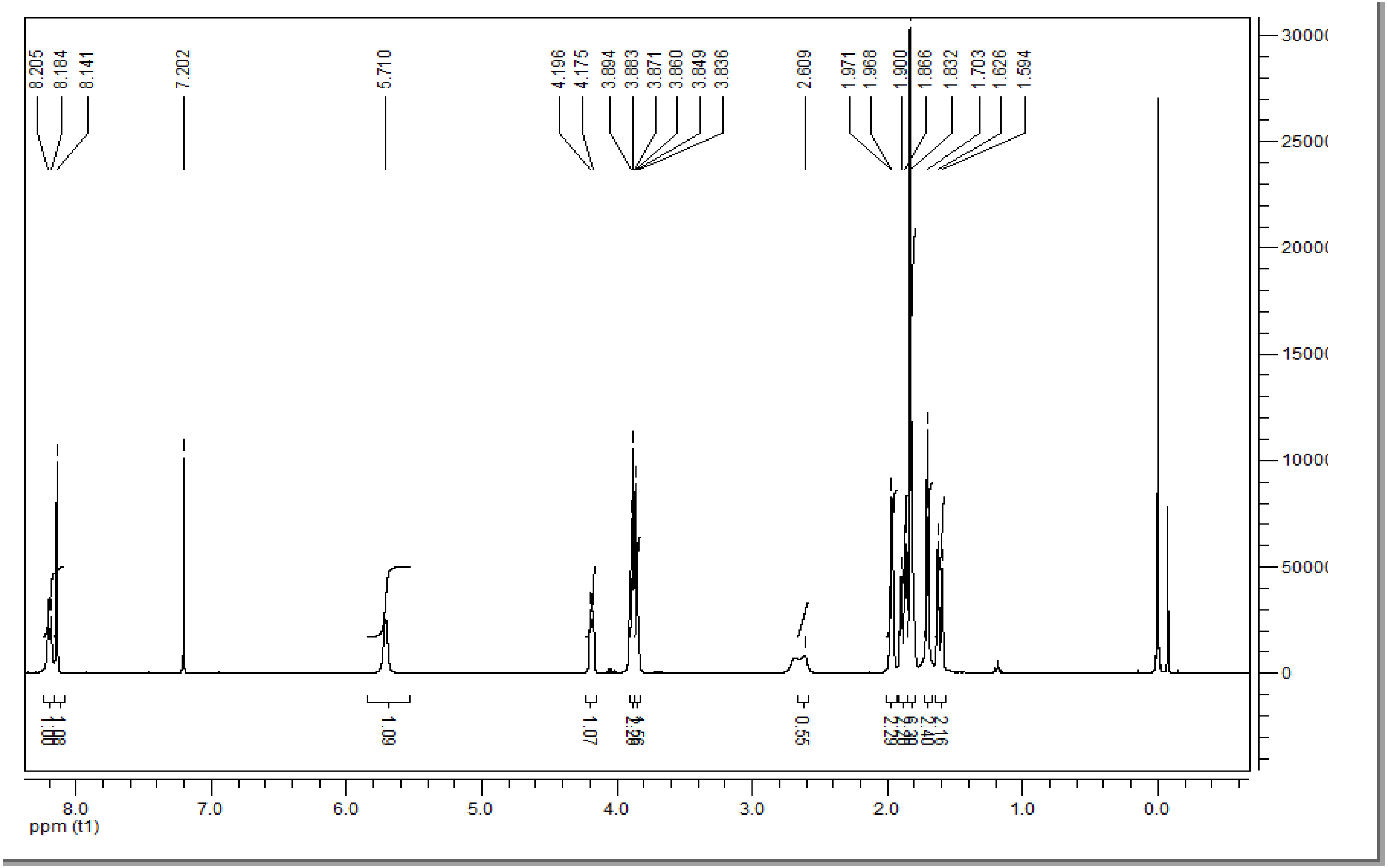

8.205
8.184
8.141
7.202
5.710
4.196
4.175
3.894
3.883
3.871
3.860
3.849
3.836
2.609
1.971
1.968
1.900
1.866
1.832
1.703
1.626
1.594
1.07
1.09
0.55
8.0
7.0
6.0
5.0
4.0
3.0
2.0
1.0
0.0
ppm (t1)
0

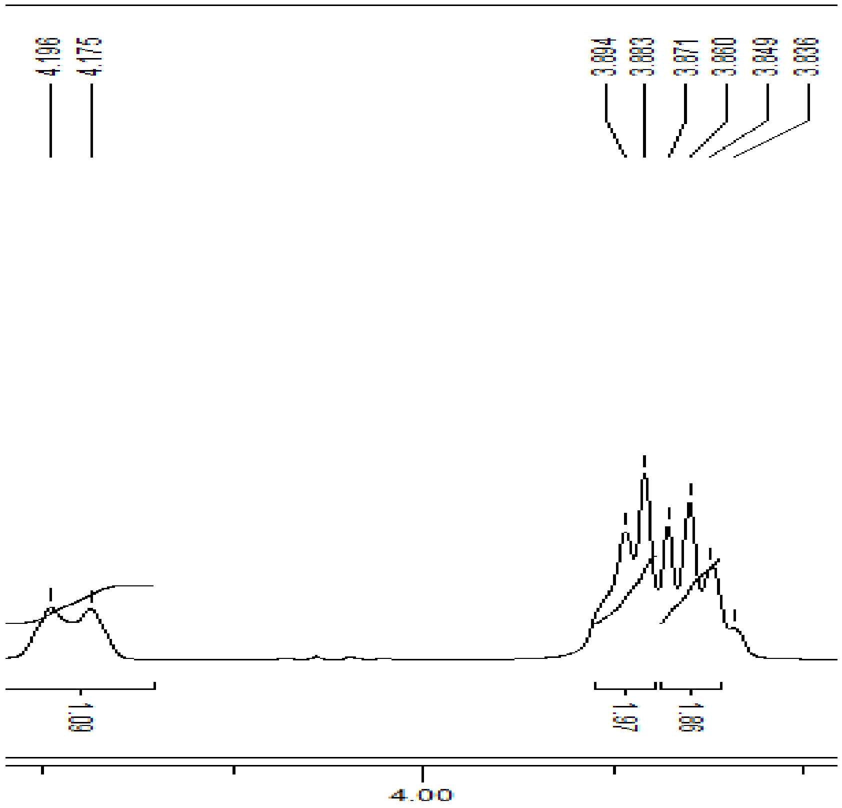
4.196
4.175
3.894
3.883
3.871
3.860
3.849
3.836
1.09
1.97
1.86
4.00

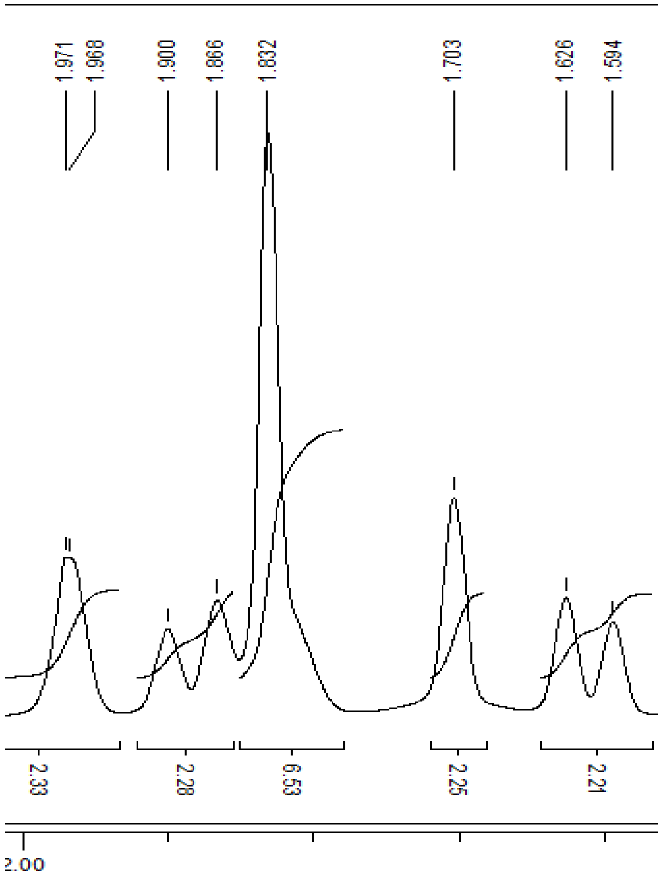
1.971
1.968
1.900
1.866
1.832
1.703
1.626
1.594
2.33
2.28
6.53
2.25
2.21
2.00

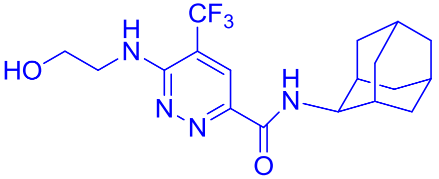
CF3
HO
H
N
N
N
H
N
O

11a

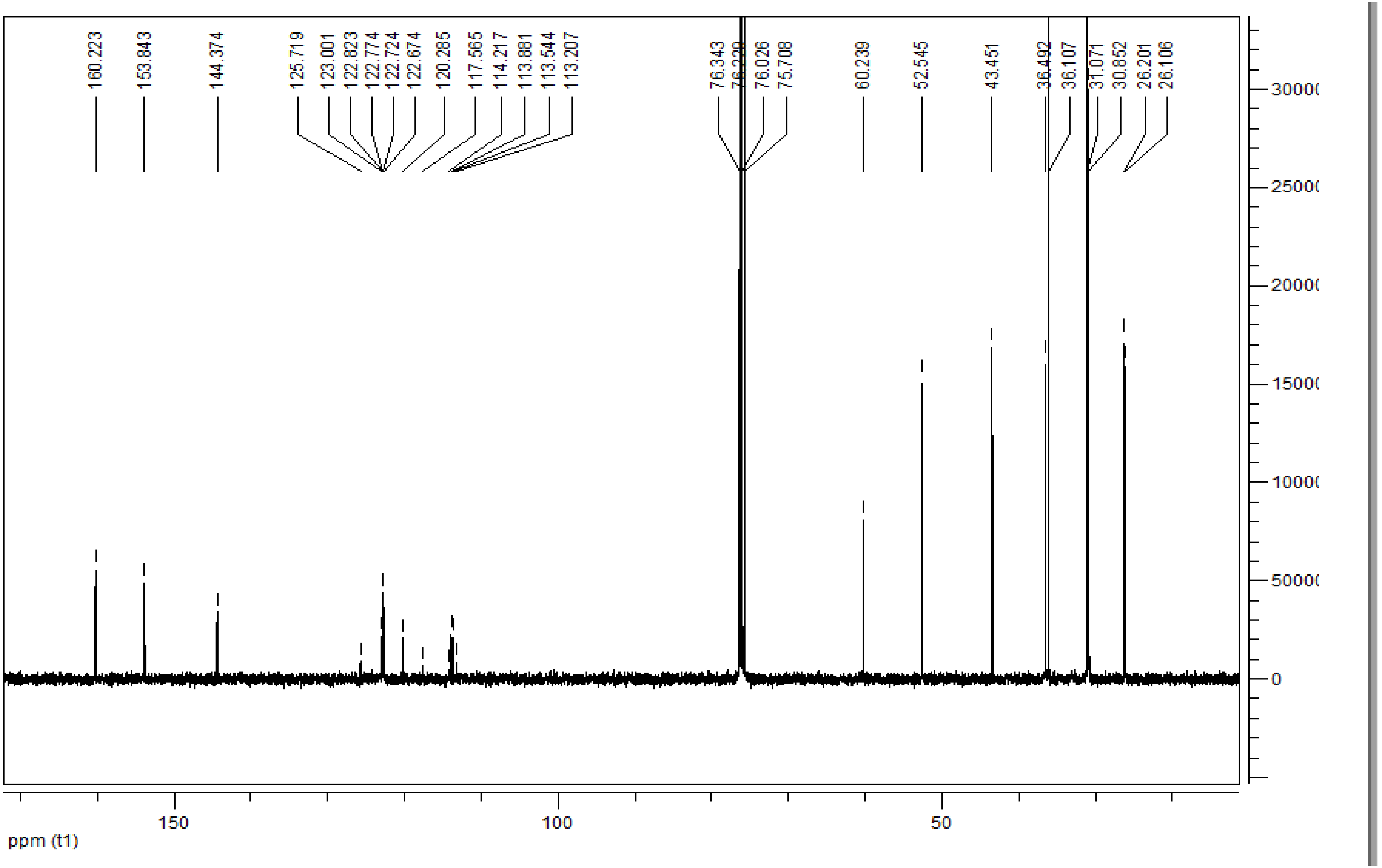
160.223
153.843
144.374
125.719
123.001
122.823
122.774
122.724
122.674
120.285
117.565
114.217
113.881
113.544
113.207
76.343
76.026
75.708
60.239
52.545
43.451
36.492
36.107
31.071
30.852
26.201
26.106
150
100
50
ppm (t1)
0

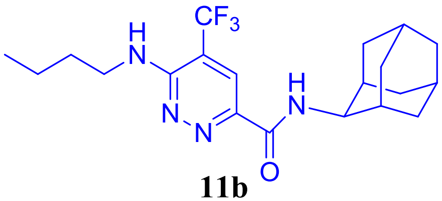

CF3
H
N
N
N
H
N
O

11b

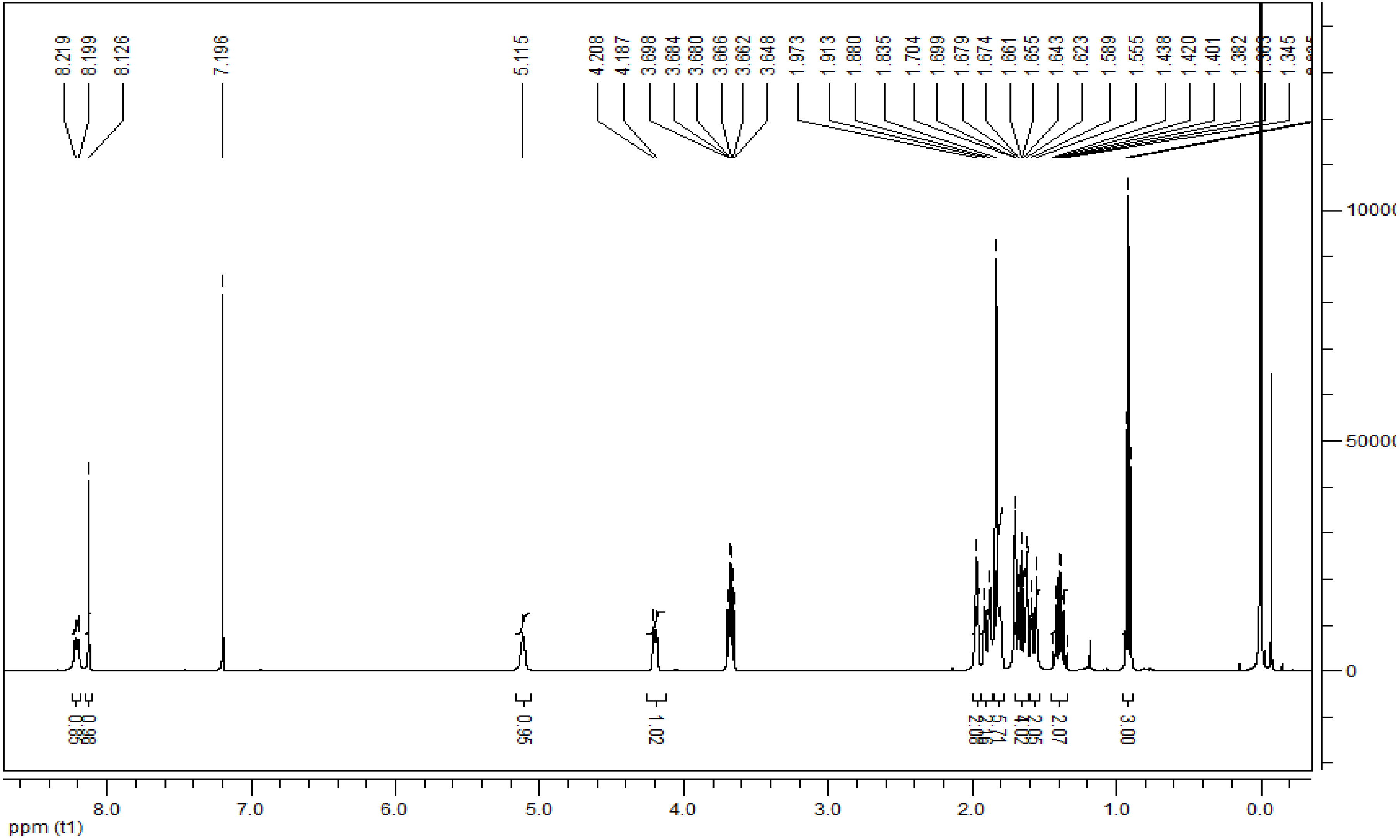

8.219
8.199
8.126
7.196
5.115
4.208
4.187
3.698
3.684
3.680
3.666
3.662
3.648
1.973
1.913
1.880
1.835
1.704
1.699
1.679
1.674
1.661
1.655
1.643
1.623
1.589
1.555
1.438
1.420
1.401
1.382
1.345
0.95
1.02
2.07
3.00
10000
50000
0
8.0
7.0
6.0
5.0
4.0
3.0
2.0
1.0
0.0
ppm (t1)

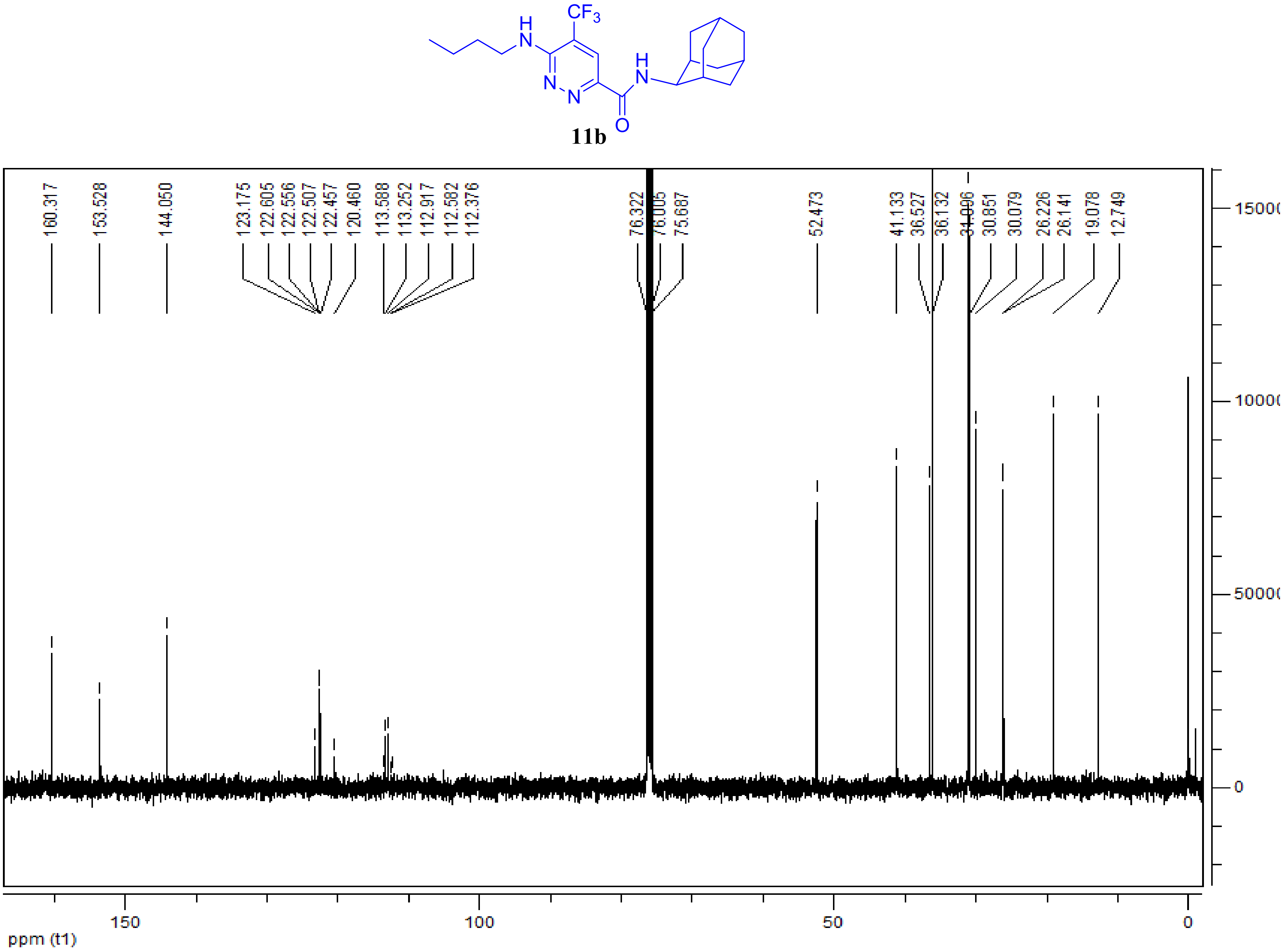
11b
160.317
153.528
144.050
123.175
122.605
122.556
122.507
122.457
120.460
113.588
113.252
112.917
112.582
112.376
76.322
76.005
75.687
52.473
41.133
36.527
36.132
34.996
30.851
30.079
26.226
26.141
19.078
12.749
150
100
50
0
ppm (t1)
0
50000
100000
150000

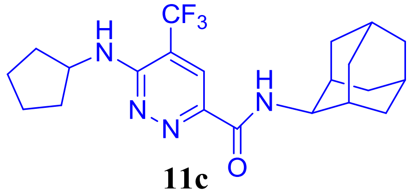

11c

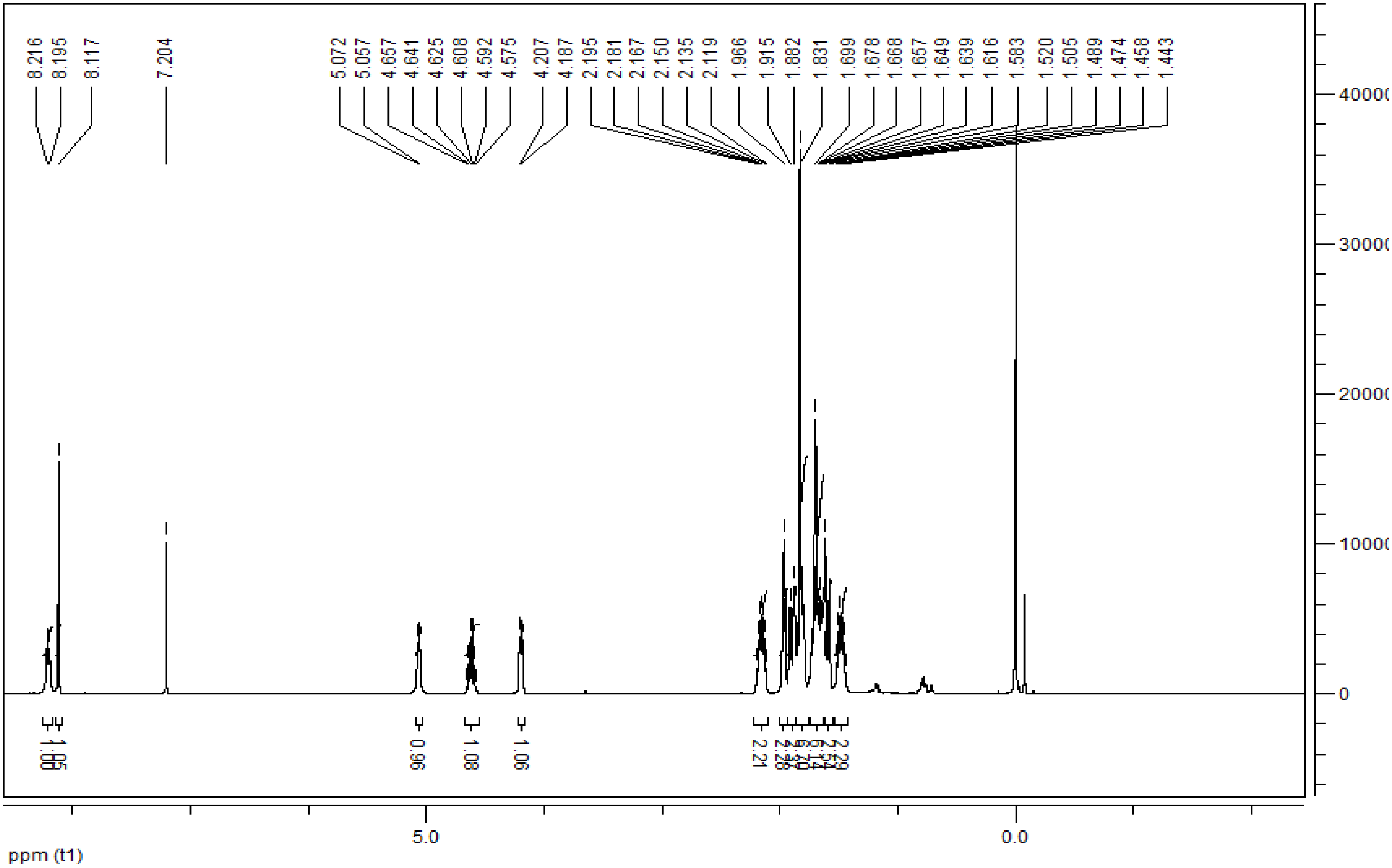

8.216
8.195
8.117
7.204
5.072
5.057
4.657
4.641
4.625
4.608
4.592
4.575
4.207
4.187
2.195
2.181
2.167
2.150
2.135
2.119
1.966
1.915
1.882
1.831
1.699
1.678
1.668
1.657
1.649
1.639
1.616
1.583
1.520
1.505
1.489
1.474
1.458
1.443
40000
30000
20000
10000
0
0.96
1.08
1.06
2.21
5.0
0.0
ppm (t1)

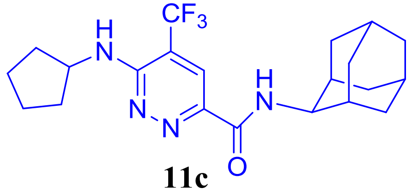
CF3
H
N
N
N
H
N
O

11c

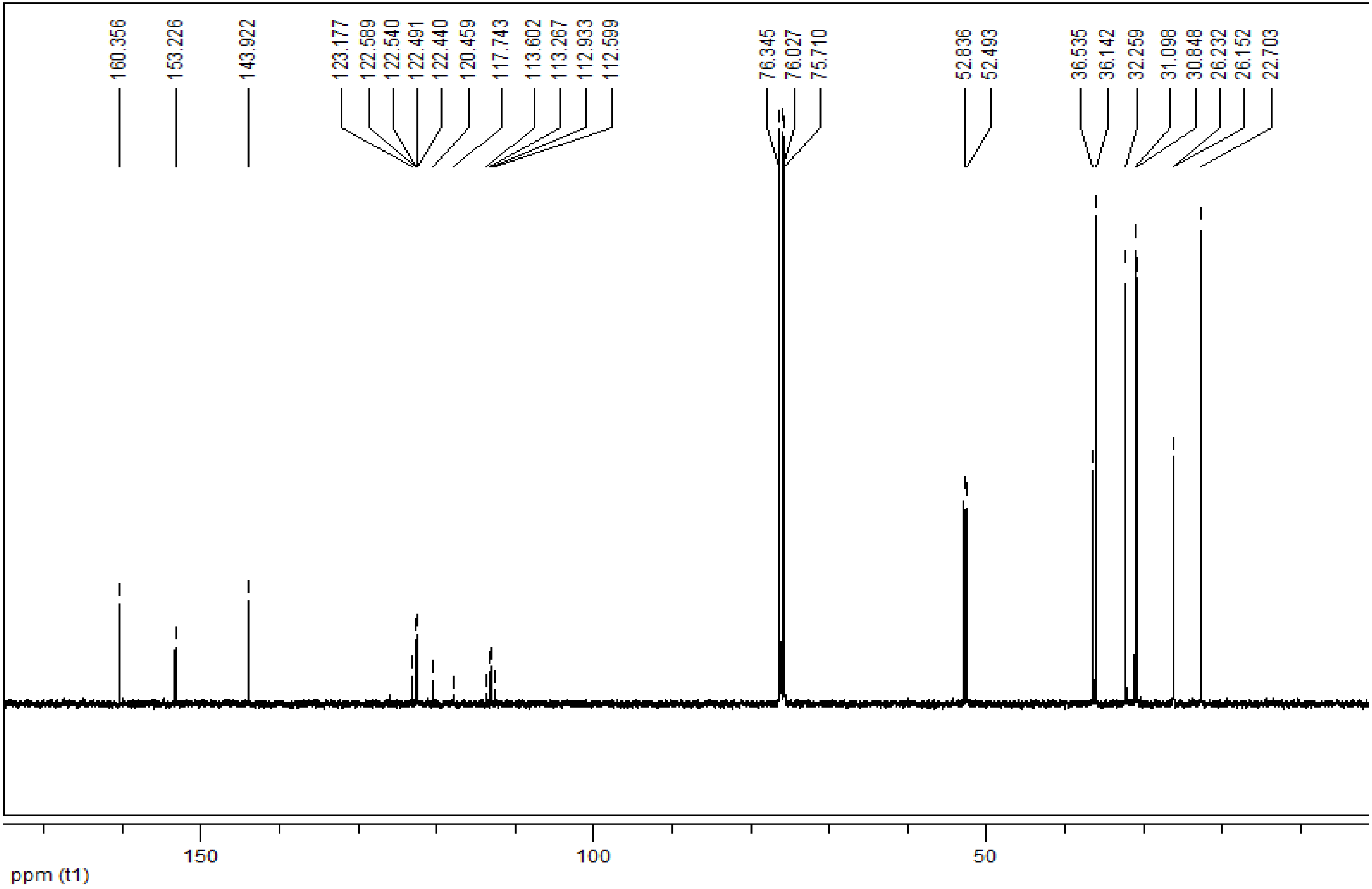
160.356
153.226
143.922
123.177
122.589
122.540
122.491
122.440
120.459
117.743
113.602
113.267
112.933
112.599
76.345
76.027
75.710
52.836
52.493
36.535
36.142
32.259
31.098
30.848
26.232
26.152
22.703
150
100
50
ppm (t1)

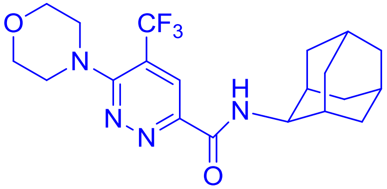

11d

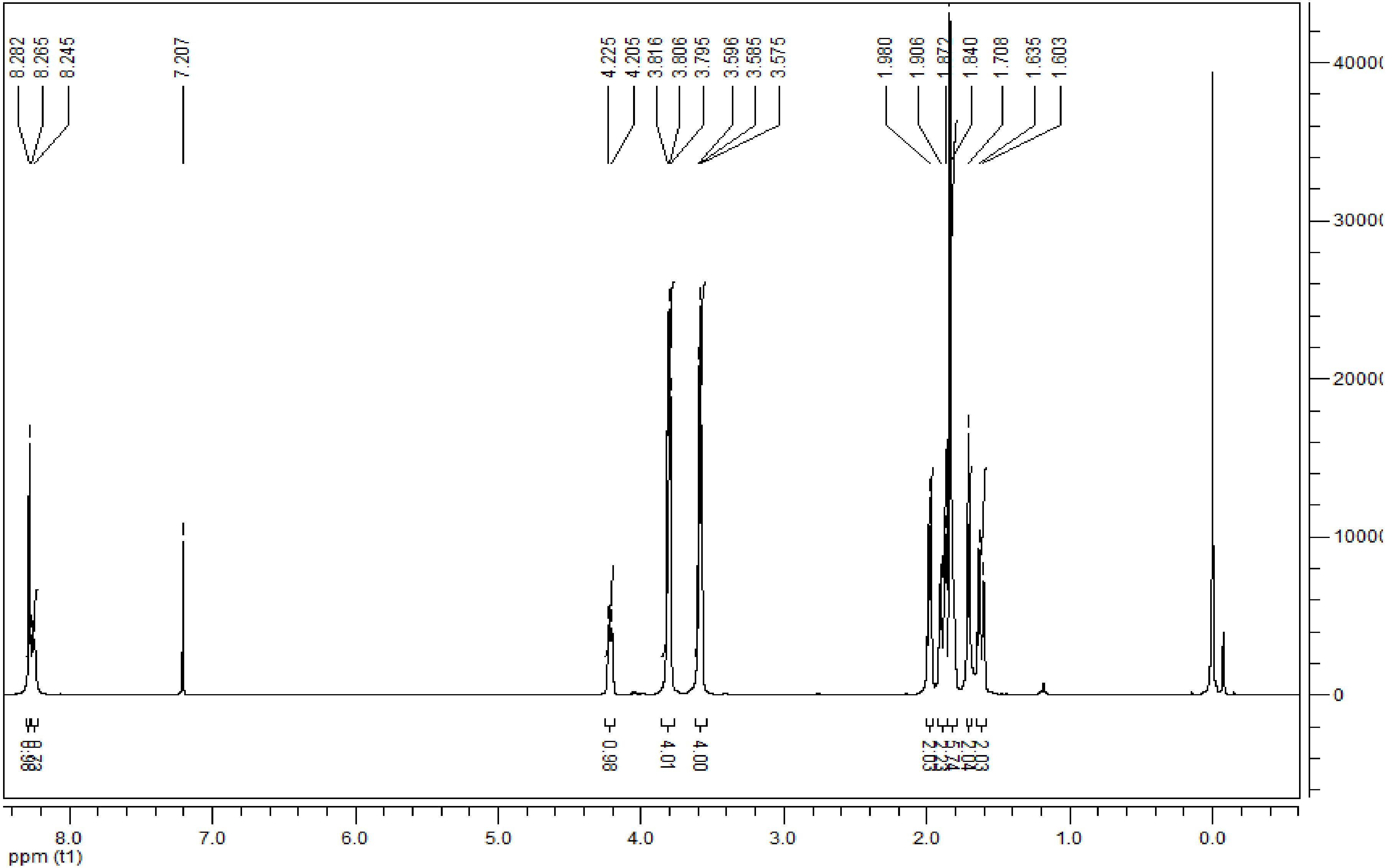
8.282
8.265
8.245
7.207
4.225
4.205
3.816
3.806
3.795
3.596
3.585
3.575
1.980
1.906
1.872
1.840
1.708
1.635
1.603
0.98
4.01
4.00
2.03
2.04
2.03
8.0
7.0
6.0
5.0
4.0
3.0
2.0
1.0
0.0
ppm (t1)

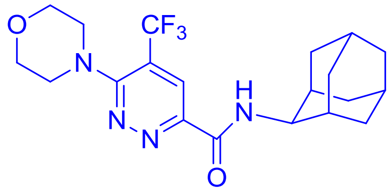

11d

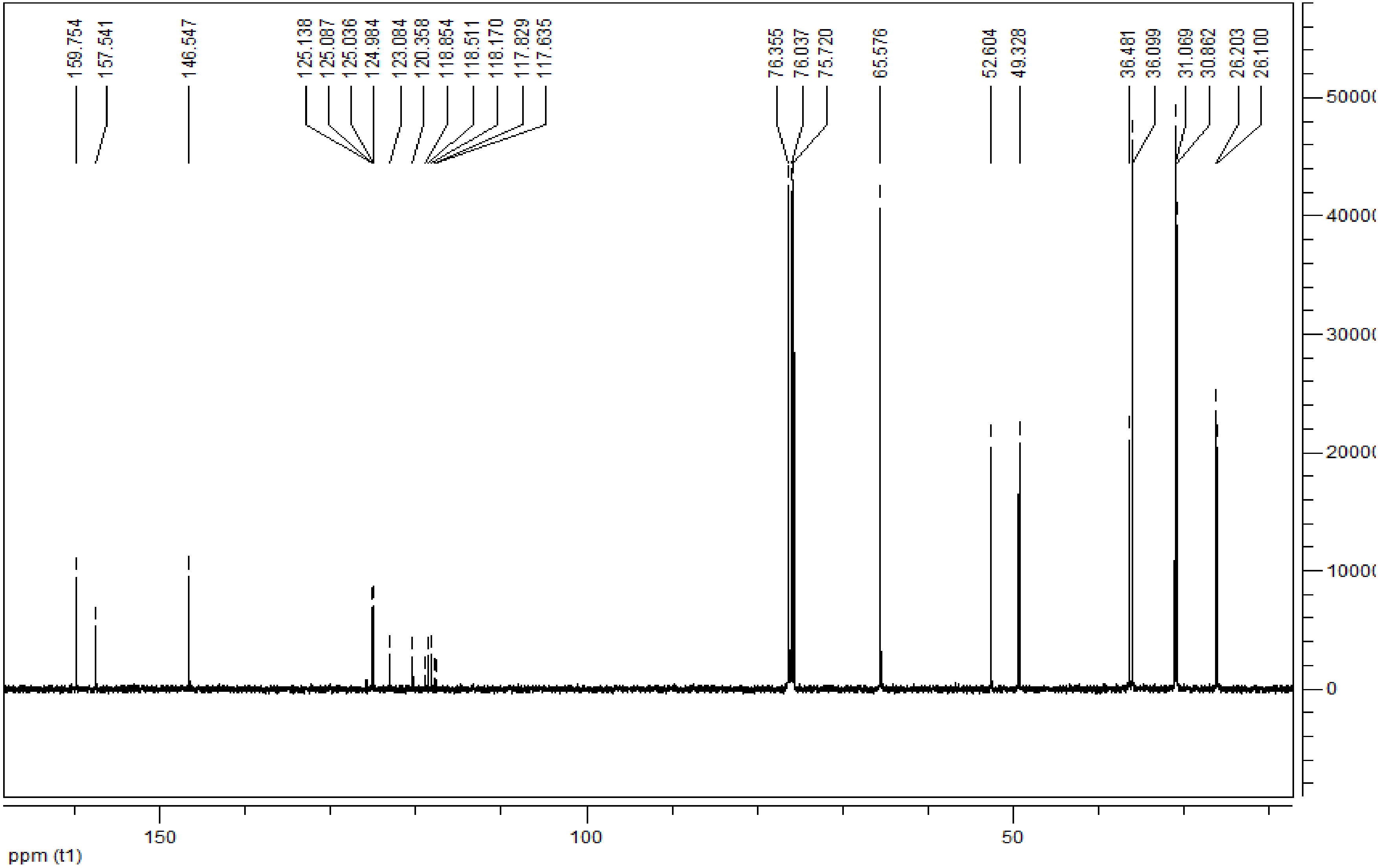
159.754
157.541
146.547
125.138
125.087
125.036
124.984
123.084
120.358
118.854
118.511
118.170
117.829
117.635
76.355
76.037
75.720
65.576
52.604
49.328
36.481
36.099
31.069
30.862
26.203
26.100
150
100
50
ppm (t1)

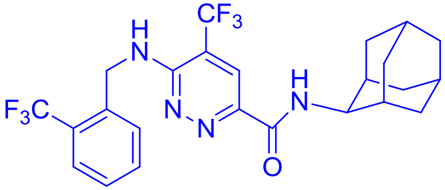
CF3
H
N
F3C
N
N
H
N
O

11e

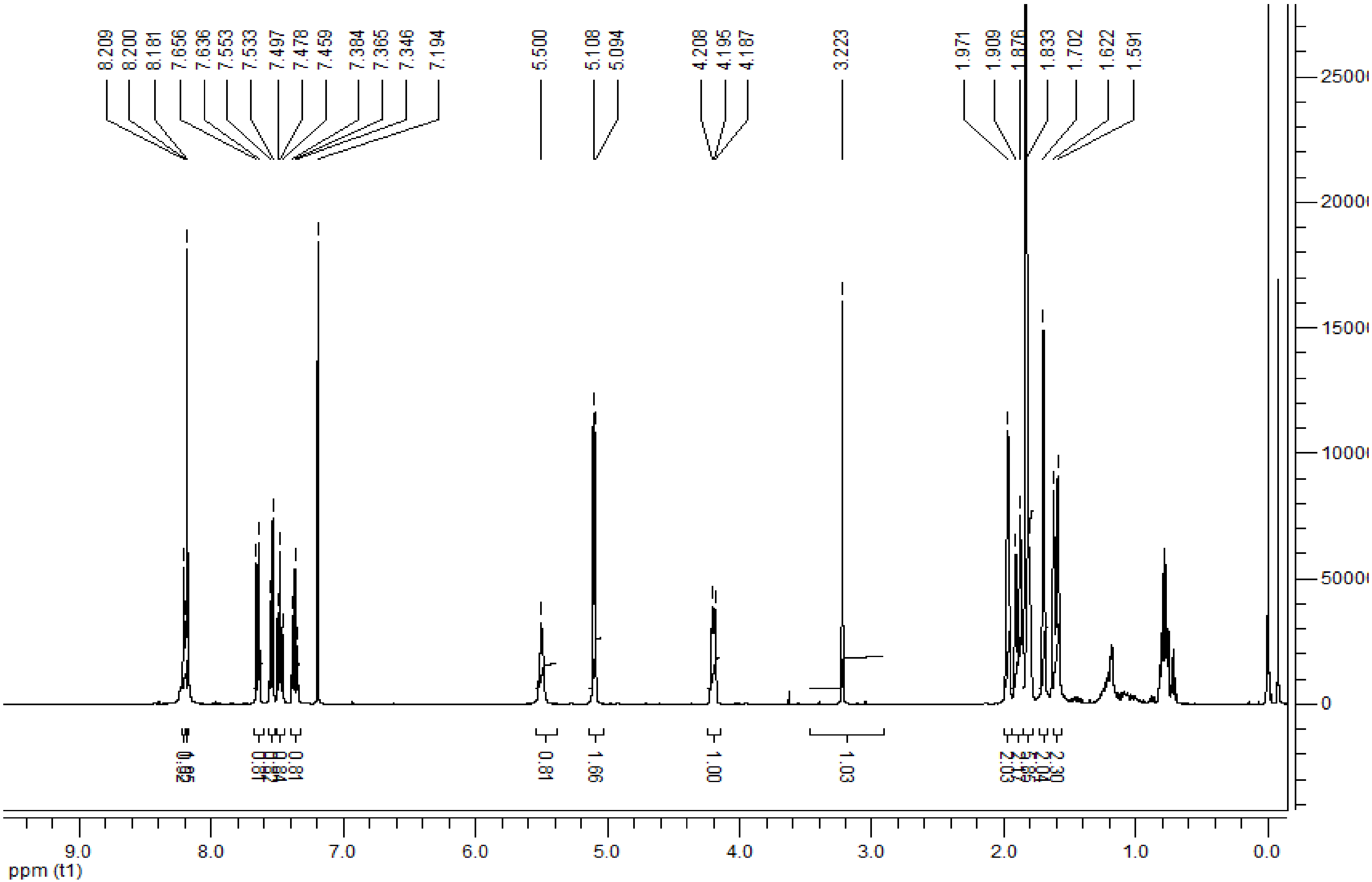
8.209
8.200
8.181
7.656
7.636
7.553
7.533
7.497
7.478
7.459
7.384
7.365
7.346
7.194
5.500
5.108
5.094
4.208
4.195
4.187
3.223
1.971
1.909
1.876
1.833
1.702
1.622
1.591
0.81
0.81
1.66
1.00
1.03
2.30
9.0
8.0
7.0
6.0
5.0
4.0
3.0
2.0
1.0
0.0
ppm (t1)
0

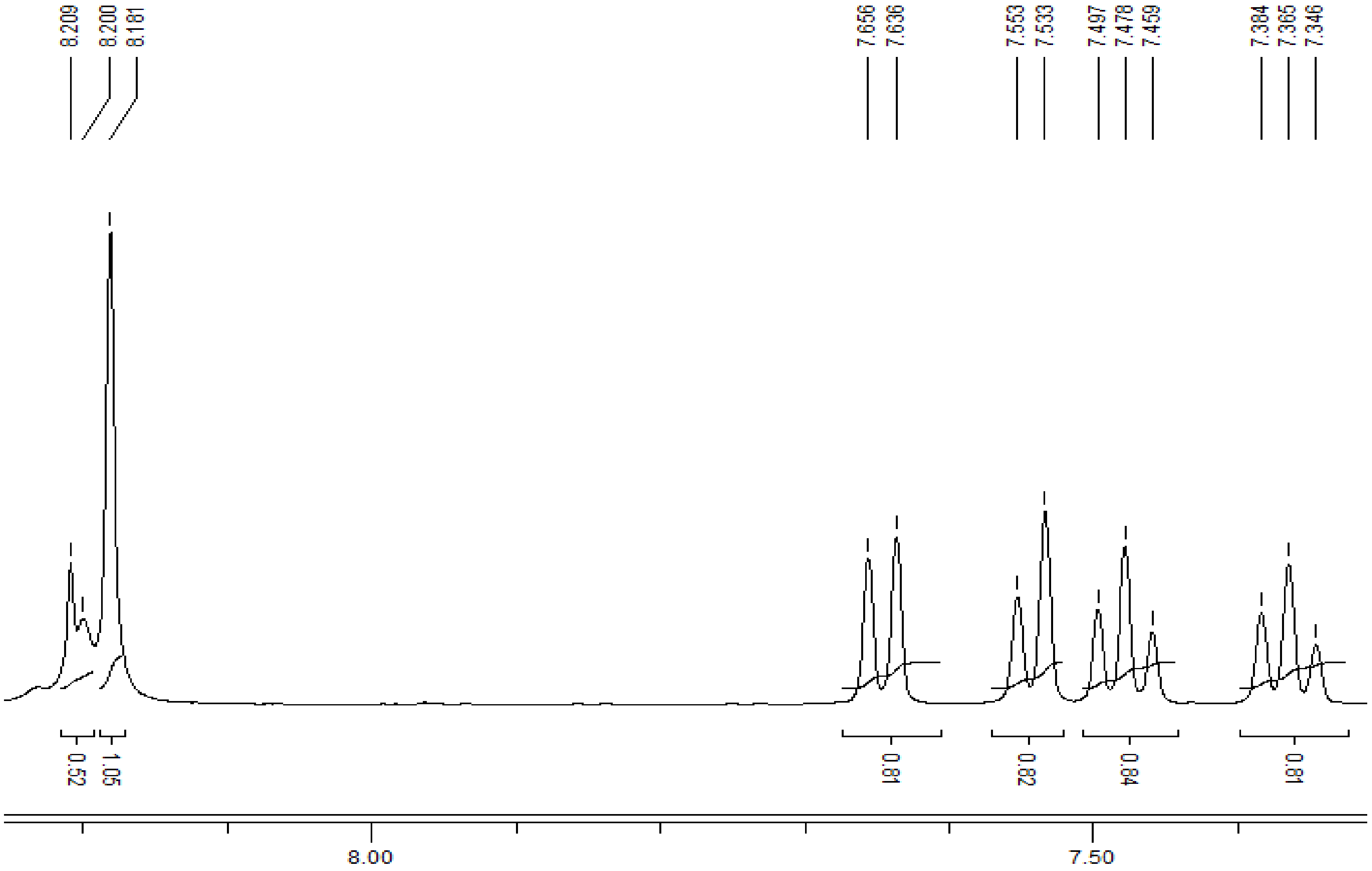

8.209
8.200
8.181
7.656
7.636
7.553
7.533
7.497
7.478
7.459
7.384
7.365
7.346
0.52
1.05
0.81
0.82
0.84
0.81
8.00
7.50

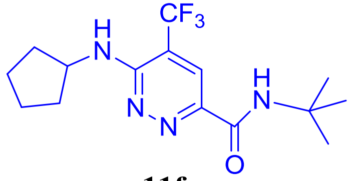

11f

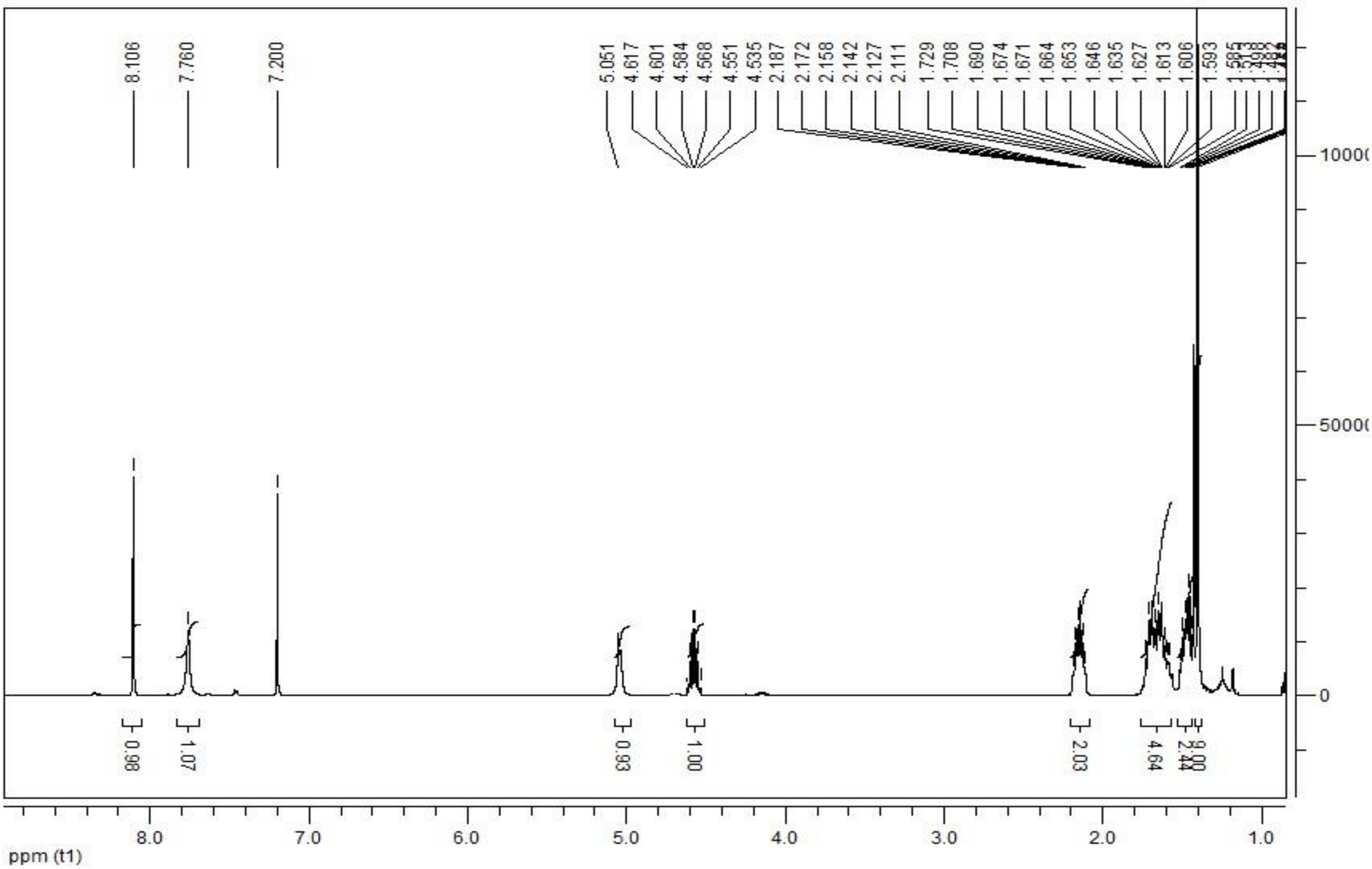

8.106
7.760
7.200
5.051
4.617
4.601
4.584
4.568
4.551
4.535
2.187
2.172
2.158
2.142
2.127
2.111
1.729
1.708
1.690
1.674
1.671
1.664
1.653
1.646
1.635
1.627
1.613
1.606
1.593
0.98
1.07
0.93
1.00
2.03
4.64
2.44
9.00
10000
5000
0
8.0
7.0
6.0
5.0
4.0
3.0
2.0
1.0
ppm (t1)

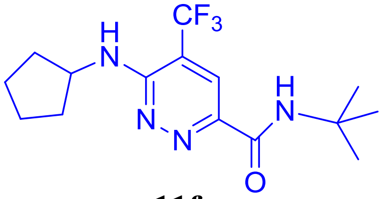

11f

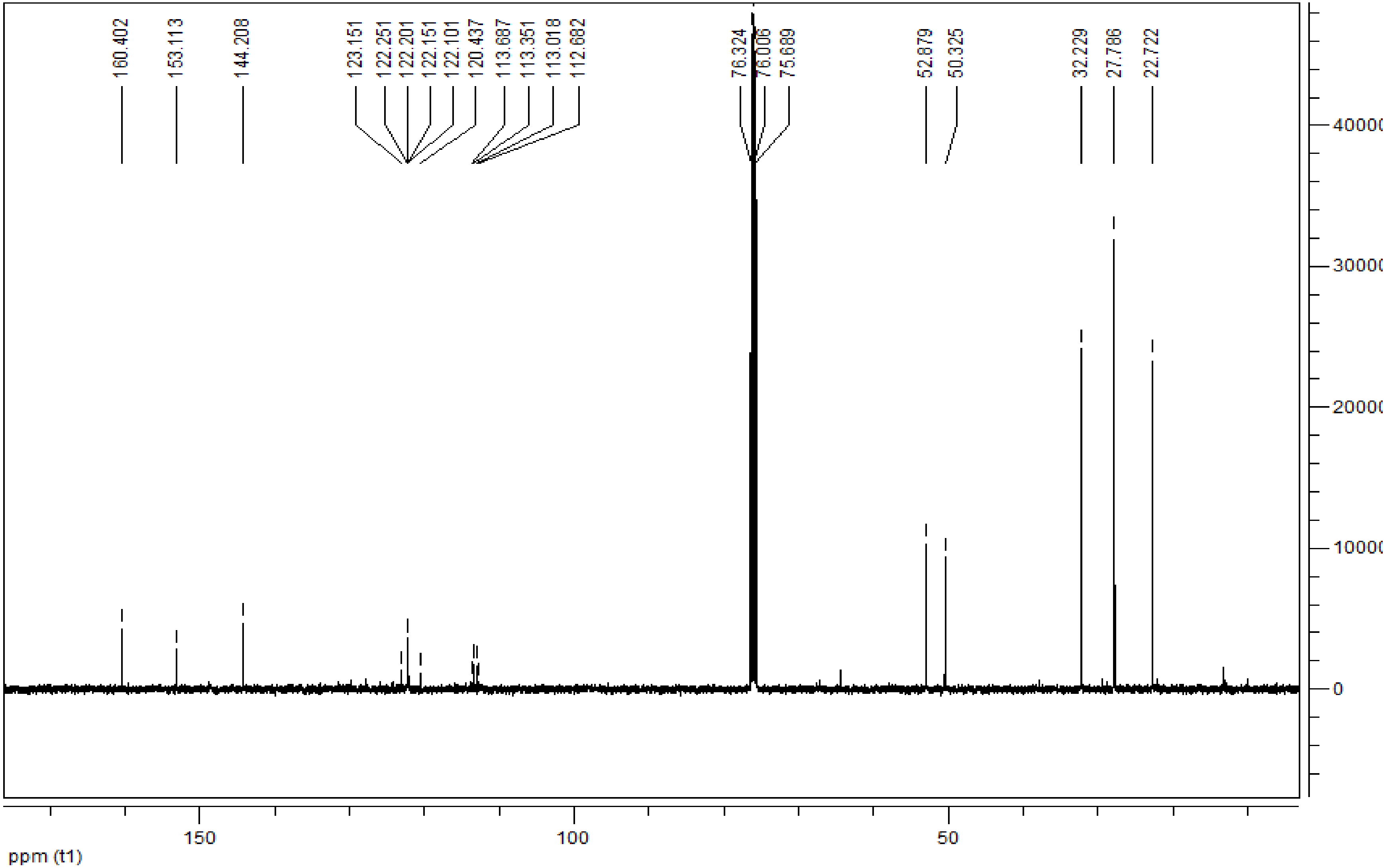

160.402
153.113
144.208
123.151
122.251
122.201
122.151
122.101
120.437
113.687
113.351
113.018
112.682
76.324
76.006
75.689
52.879
50.325
32.229
27.786
22.722
40000
30000
20000
10000
0
150
100
50
ppm (t1)

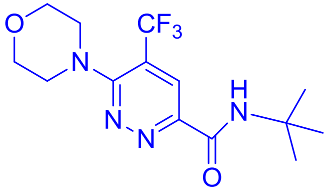
O
N
CF3
N
N
H
N
O

11g

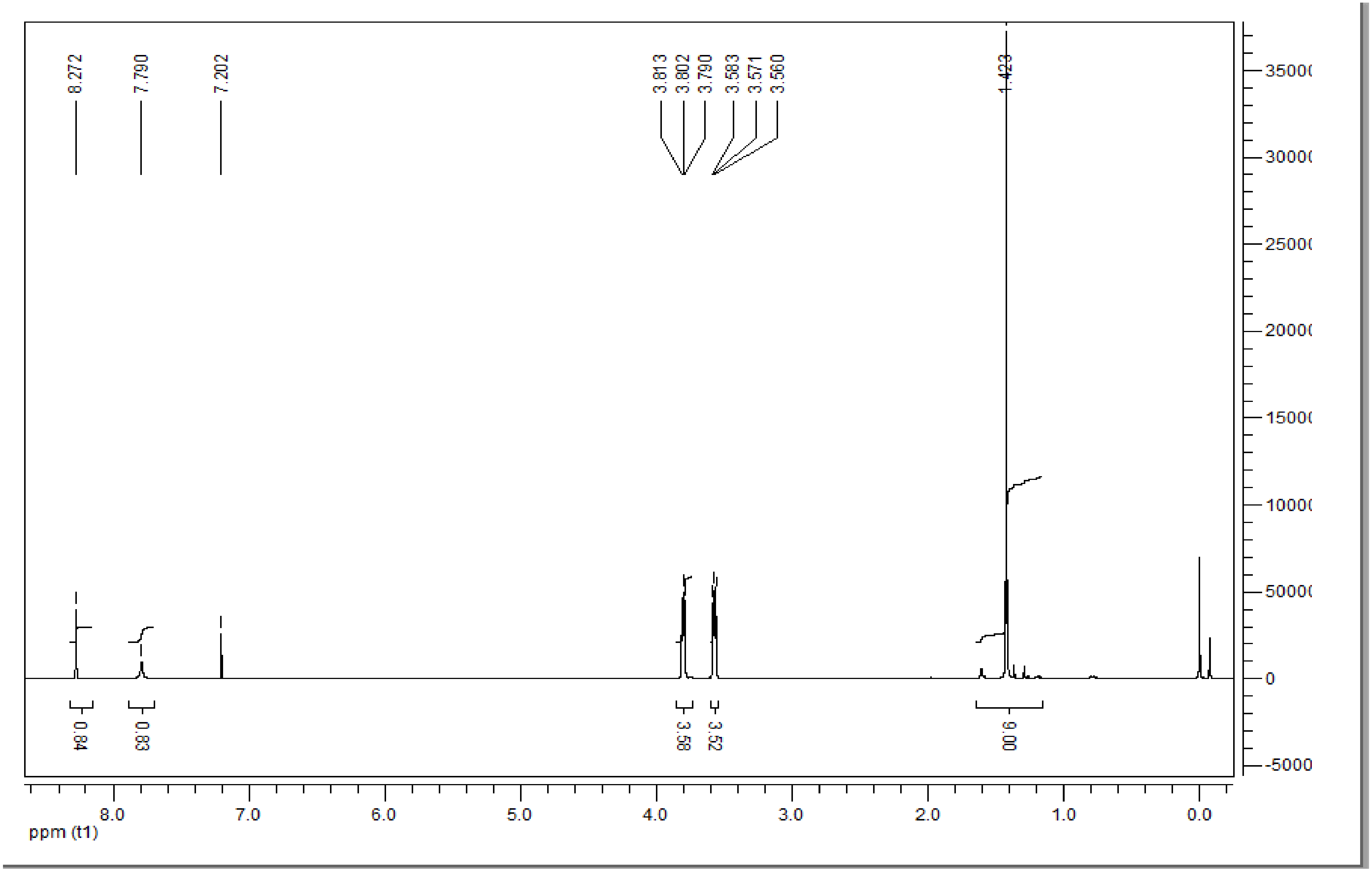
8.272
7.790
7.202
3.813
3.802
3.790
3.583
3.571
3.560
1.423
0.84
0.83
3.58
3.52
9.00
8.0
7.0
6.0
5.0
4.0
3.0
2.0
1.0
0.0
ppm (t1)
0
-5000

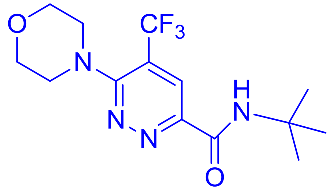

11g

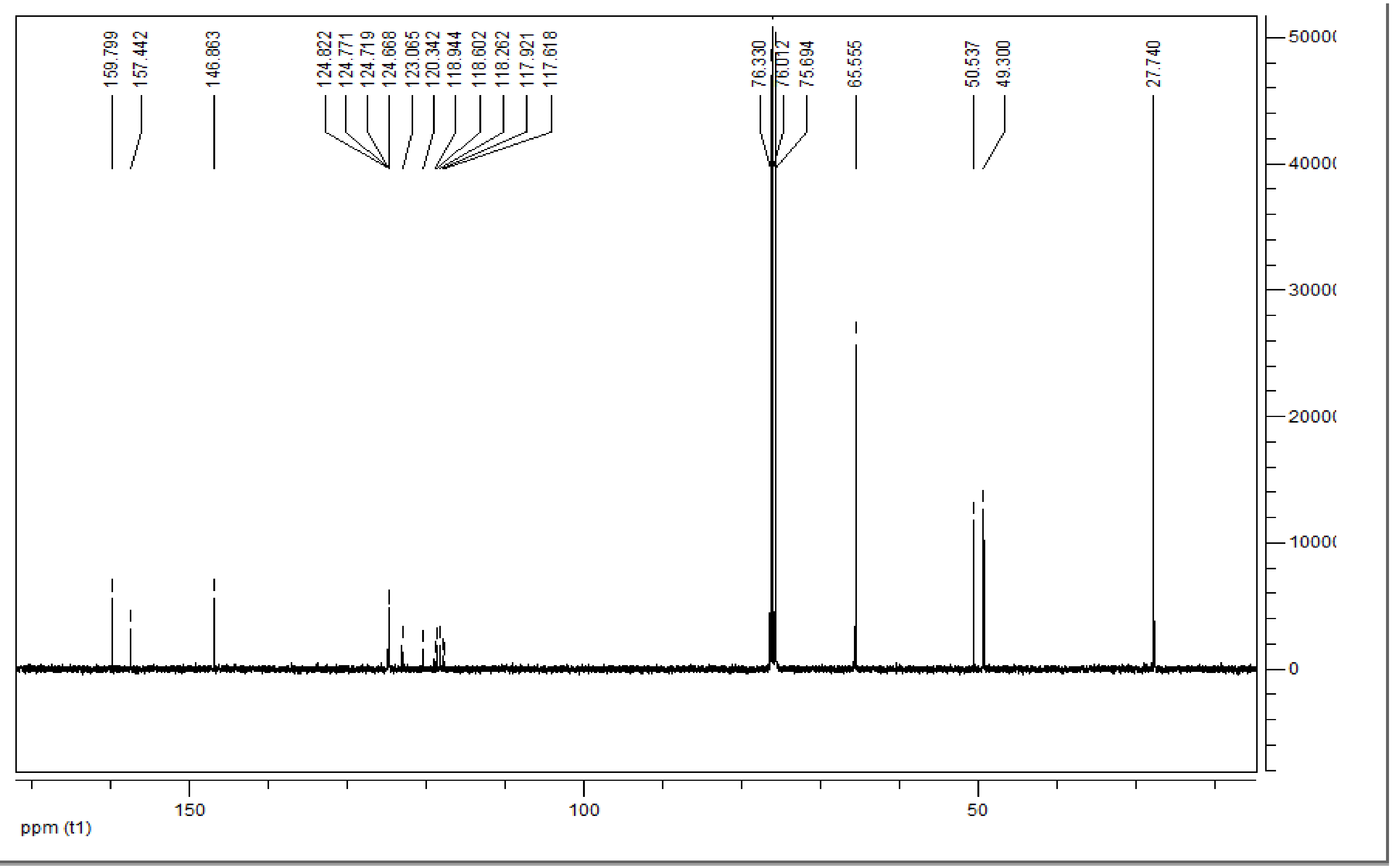
159.799
157.442
146.863
124.822
124.771
124.719
124.668
123.065
120.342
118.944
118.602
118.262
117.921
117.618
76.330
76.012
75.694
65.555
50.537
49.300
27.740
50000
40000
30000
20000
10000
0
150
100
50
ppm (t1)

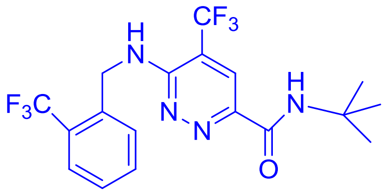
CF3
H
N
F3C
N
N
H
N
O

11h

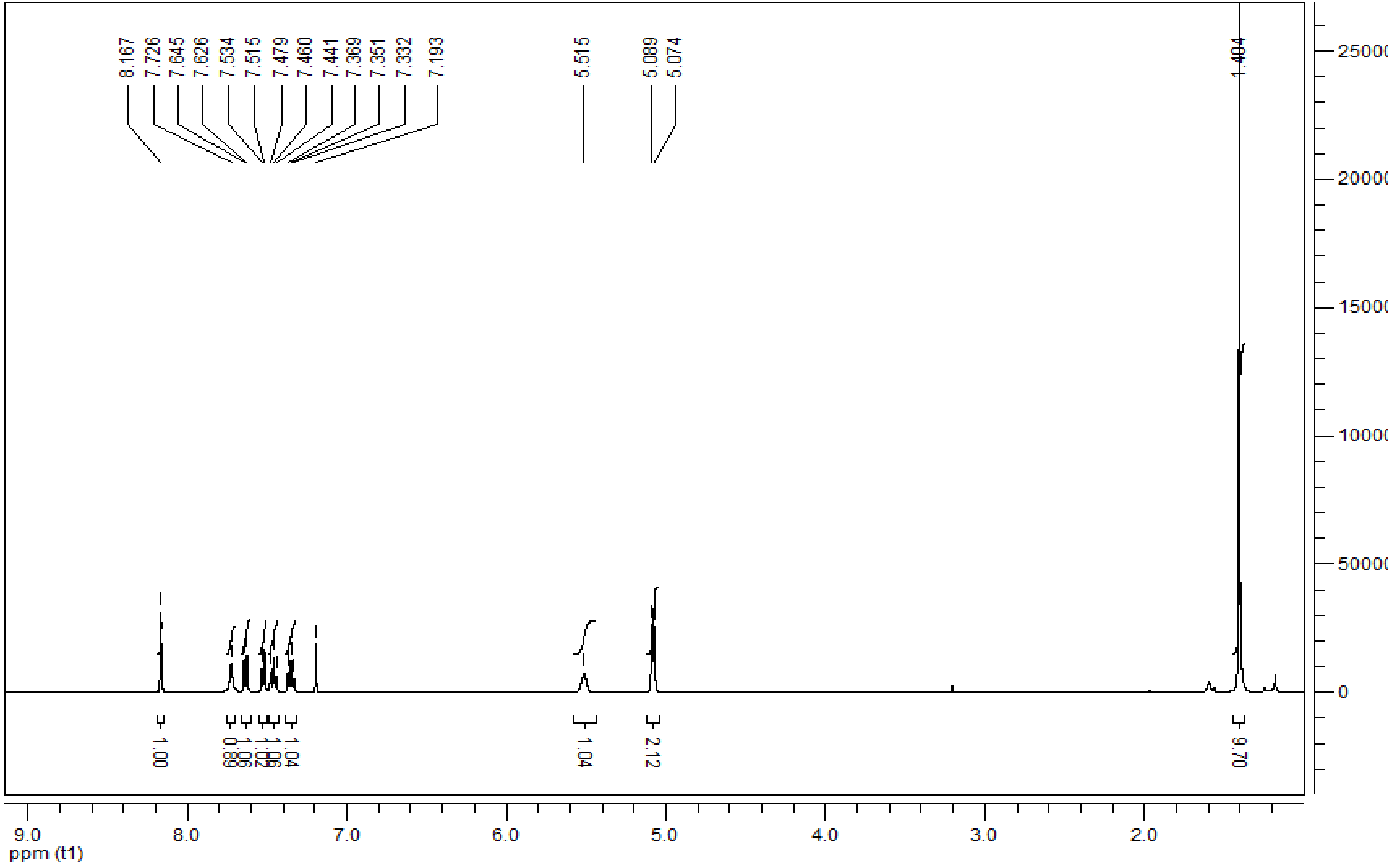
8.167
7.726
7.645
7.626
7.534
7.515
7.479
7.460
7.441
7.369
7.351
7.332
7.193
5.515
5.089
5.074
1.404
1.00
0.89
1.06
1.02
1.06
1.04
1.04
2.12
9.70
9.0
8.0
7.0
6.0
5.0
4.0
3.0
2.0
ppm (t1)
0

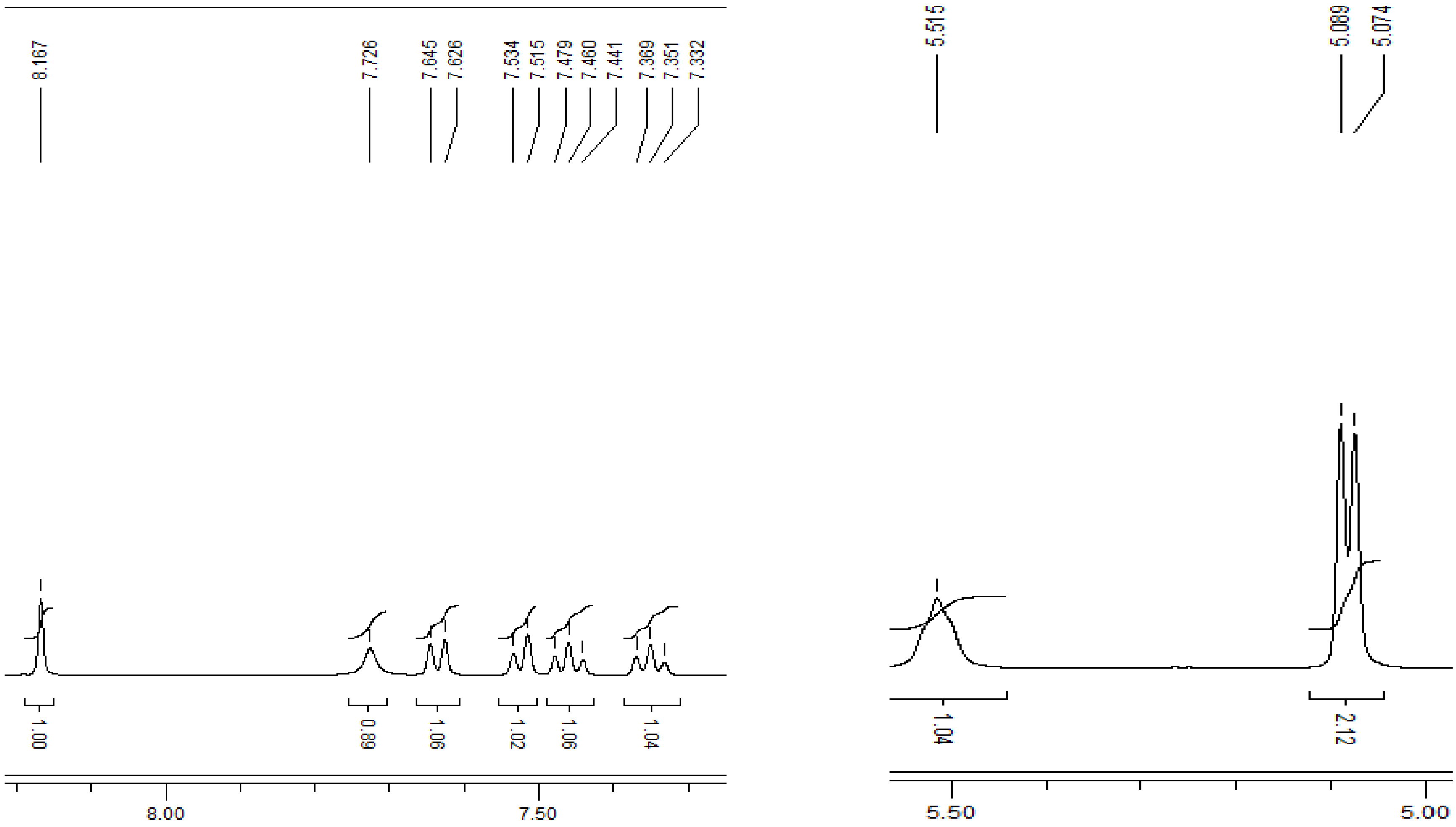

8.167
7.726
7.645
7.626
7.534
7.515
7.479
7.460
7.441
7.369
7.351
7.332
1.00
0.89
1.06
1.02
1.06
1.04
8.00
7.50
5.515
5.089
5.074
1.04
2.12
5.50
5.00

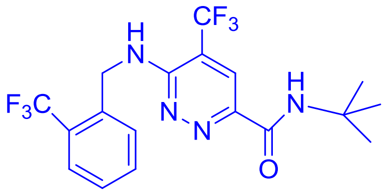

11h

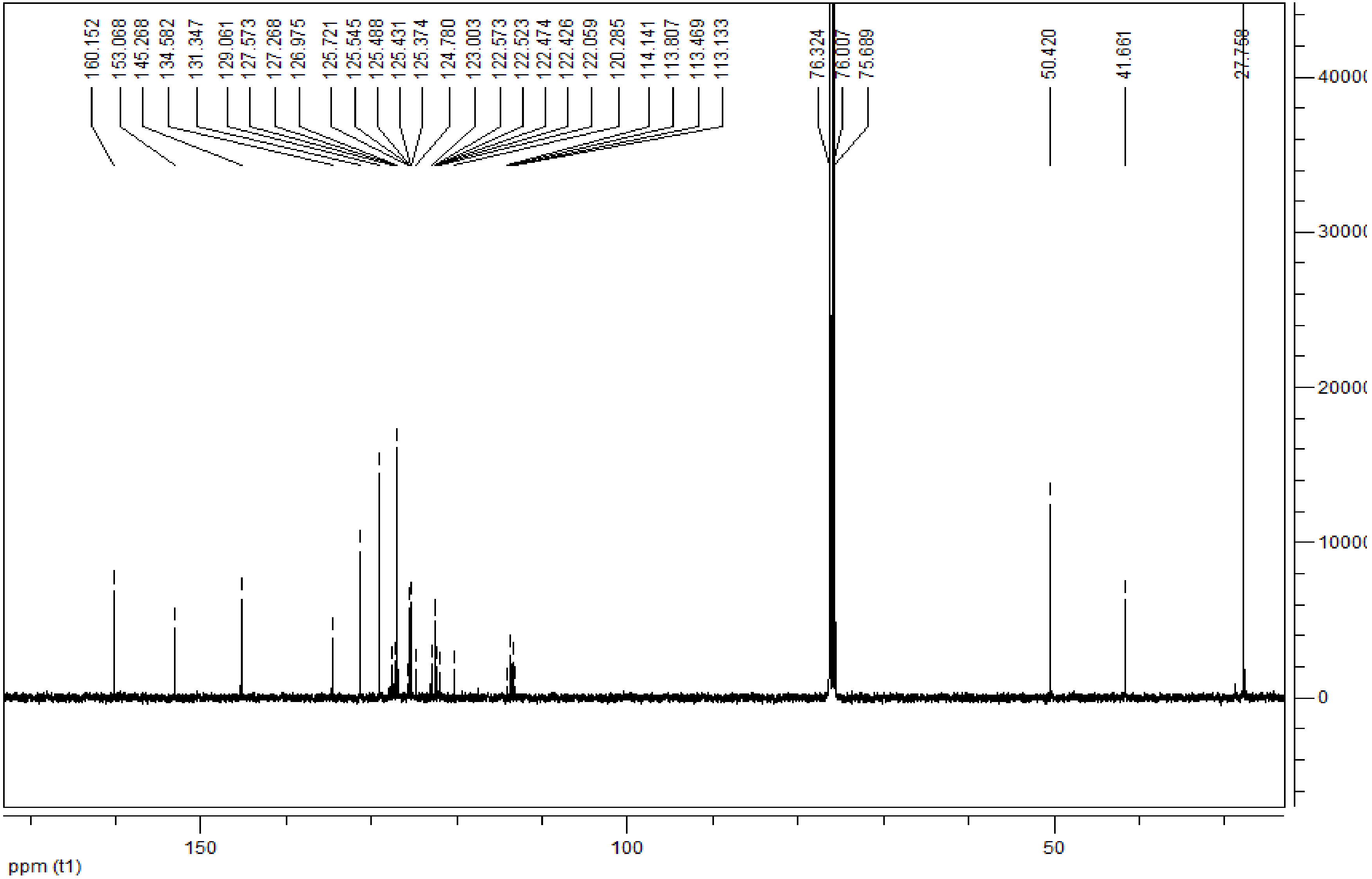
160.152
153.068
145.268
134.582
131.347
129.061
127.573
127.268
126.975
125.721
125.545
125.488
125.431
125.374
124.780
123.003
122.573
122.523
122.474
122.426
122.059
120.285
114.141
113.807
113.469
113.133
76.324
76.007
75.689
50.420
41.661
27.758
150
100
50
ppm (t1)
40000
30000
20000
10000
0

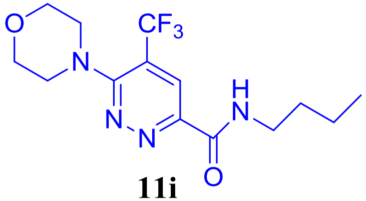
CF3
O
N
N
N
H
N
O

11i

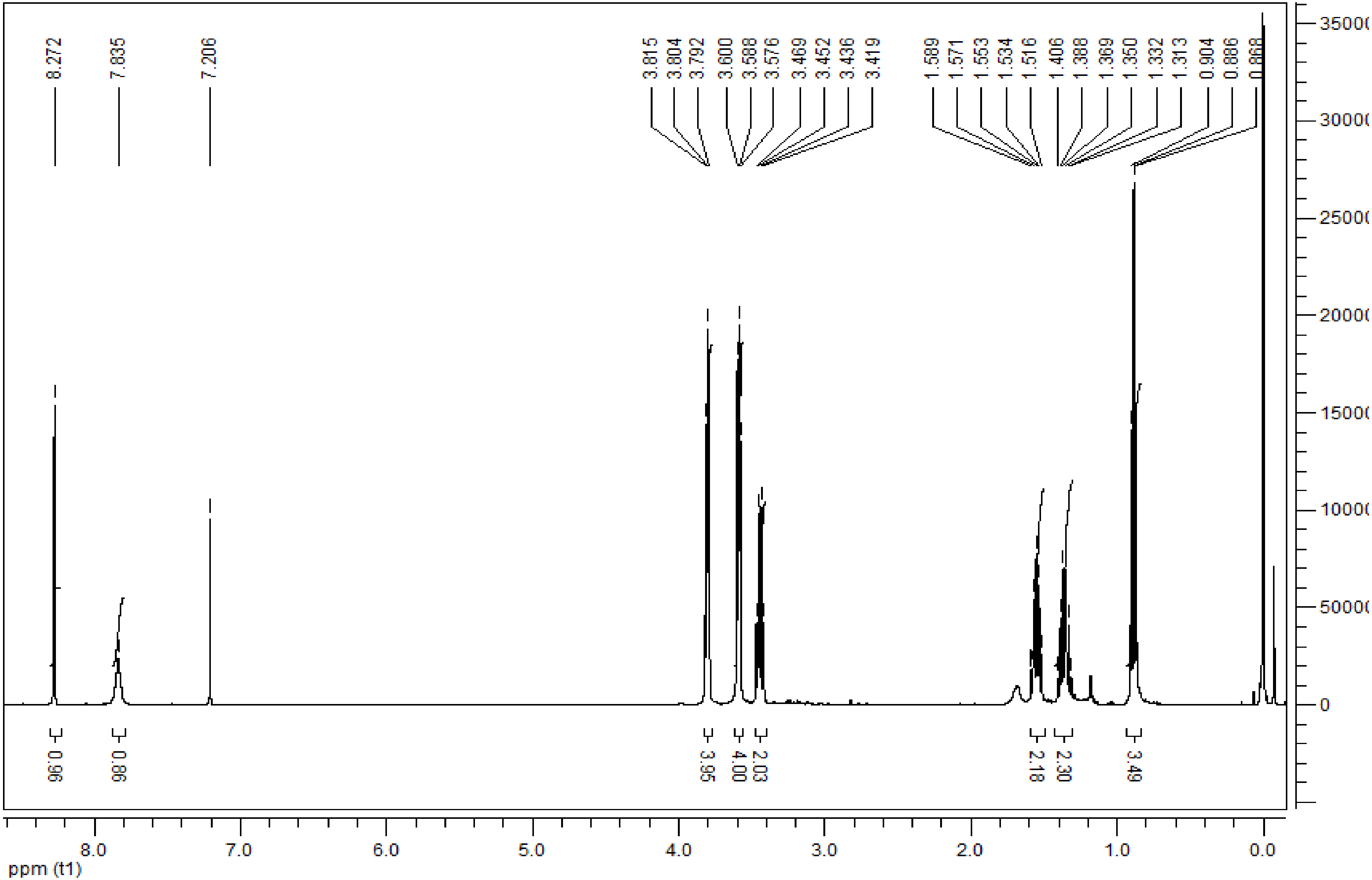
8.272
7.835
7.206
3.815
3.804
3.792
3.600
3.588
3.576
3.469
3.452
3.436
3.419
1.589
1.571
1.553
1.534
1.516
1.406
1.388
1.369
1.350
1.332
1.313
0.904
0.886
0.868
0.96
0.86
3.95
4.00
2.03
2.18
2.30
3.49
8.0
7.0
6.0
5.0
4.0
3.0
2.0
1.0
0.0
ppm (t1)

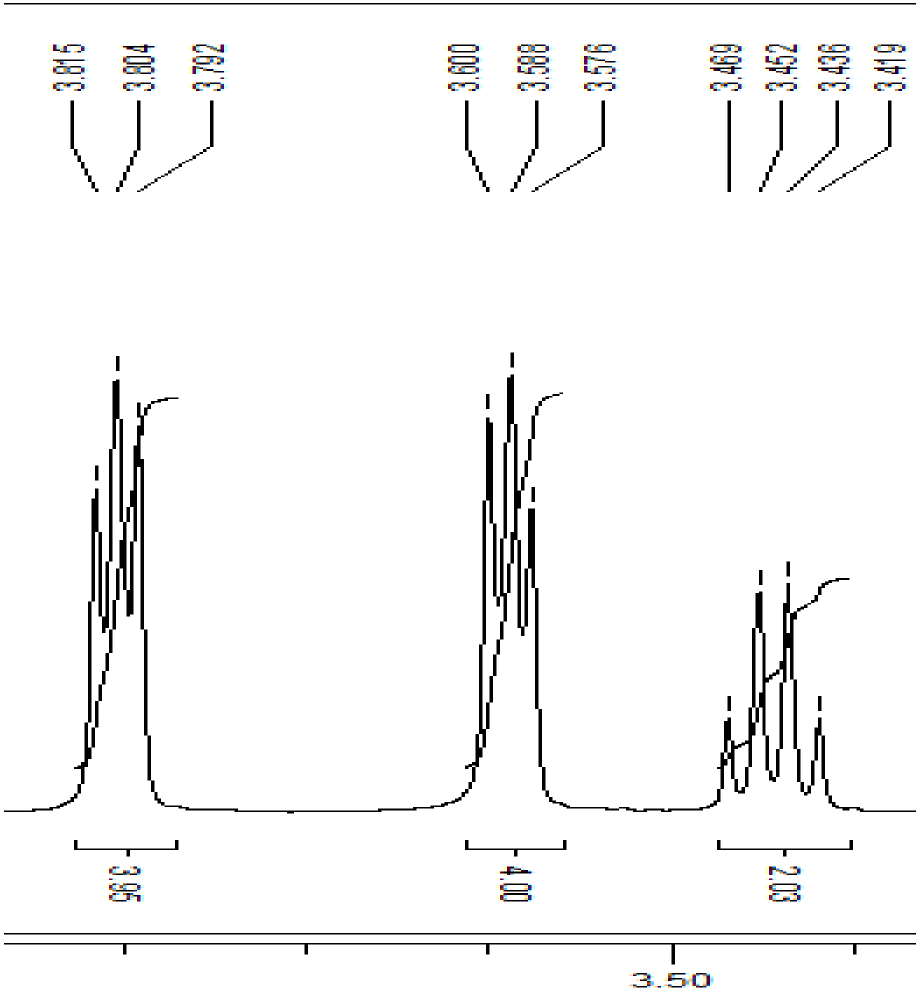

3.815
3.804
3.792
3.600
3.588
3.576
3.469
3.452
3.436
3.419
3.95
4.00
2.03
3.50

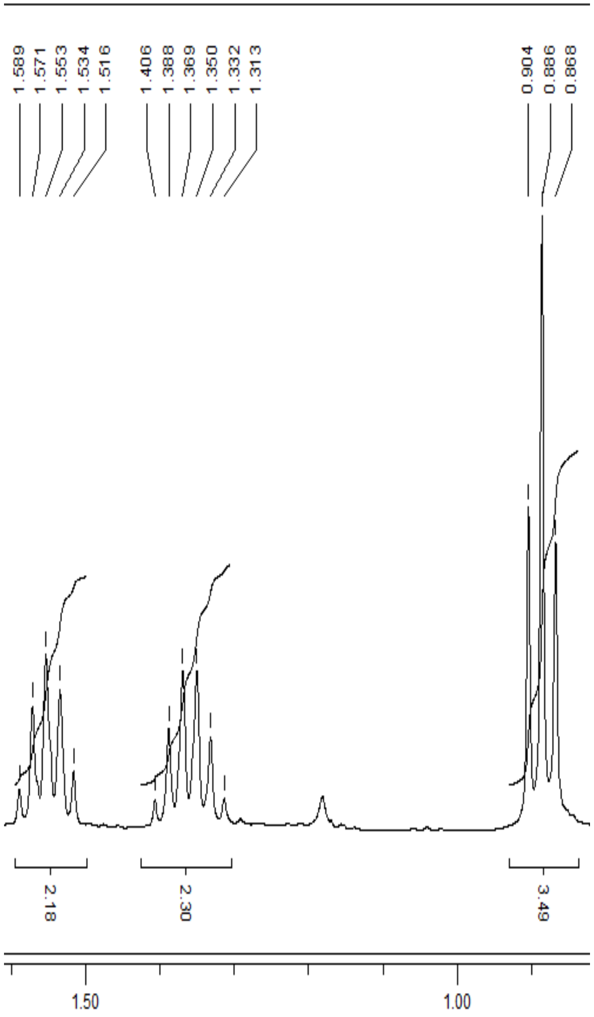

1.589
1.571
1.553
1.534
1.516
1.406
1.388
1.369
1.350
1.332
1.313
0.904
0.886
0.868
2.18
2.30
3.49
1.50
1.00

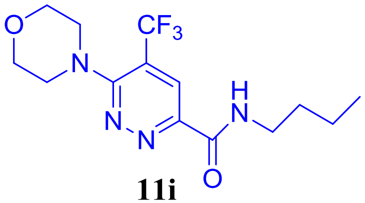
O
N
CF3
H
N
N
N
O

11i

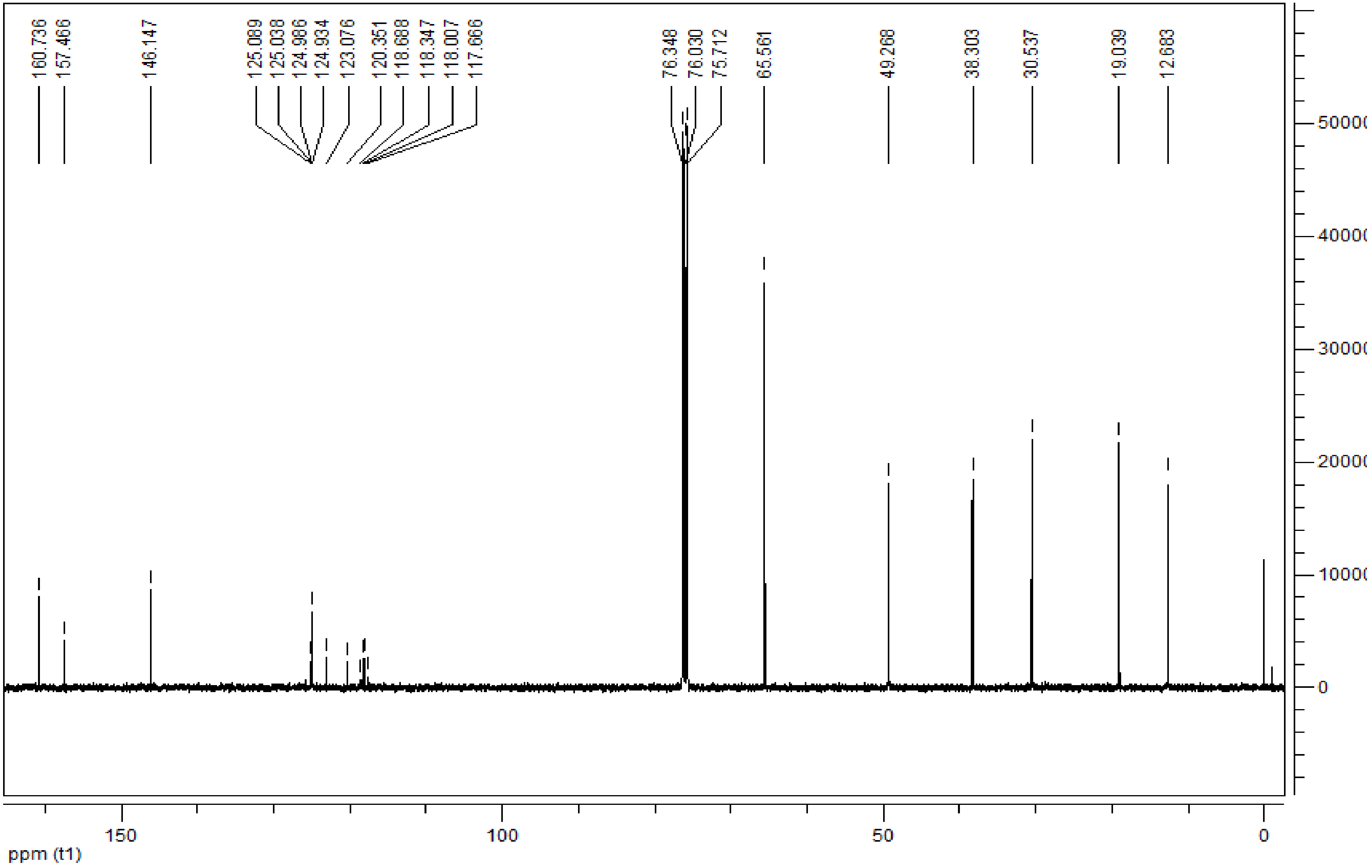
160.736
157.466
146.147
125.089
125.038
124.986
124.934
123.076
120.351
118.688
118.347
118.007
117.666
76.348
76.030
75.712
65.561
49.268
38.303
30.537
19.039
12.683
50000
40000
30000
20000
10000
0
150
100
50
0
ppm (t1)

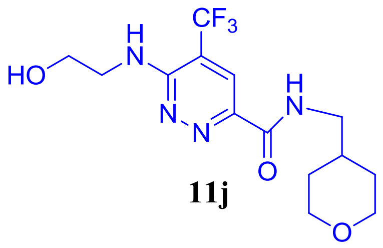
CF3
H
N
HO
N
N
H
N
O
O

11j

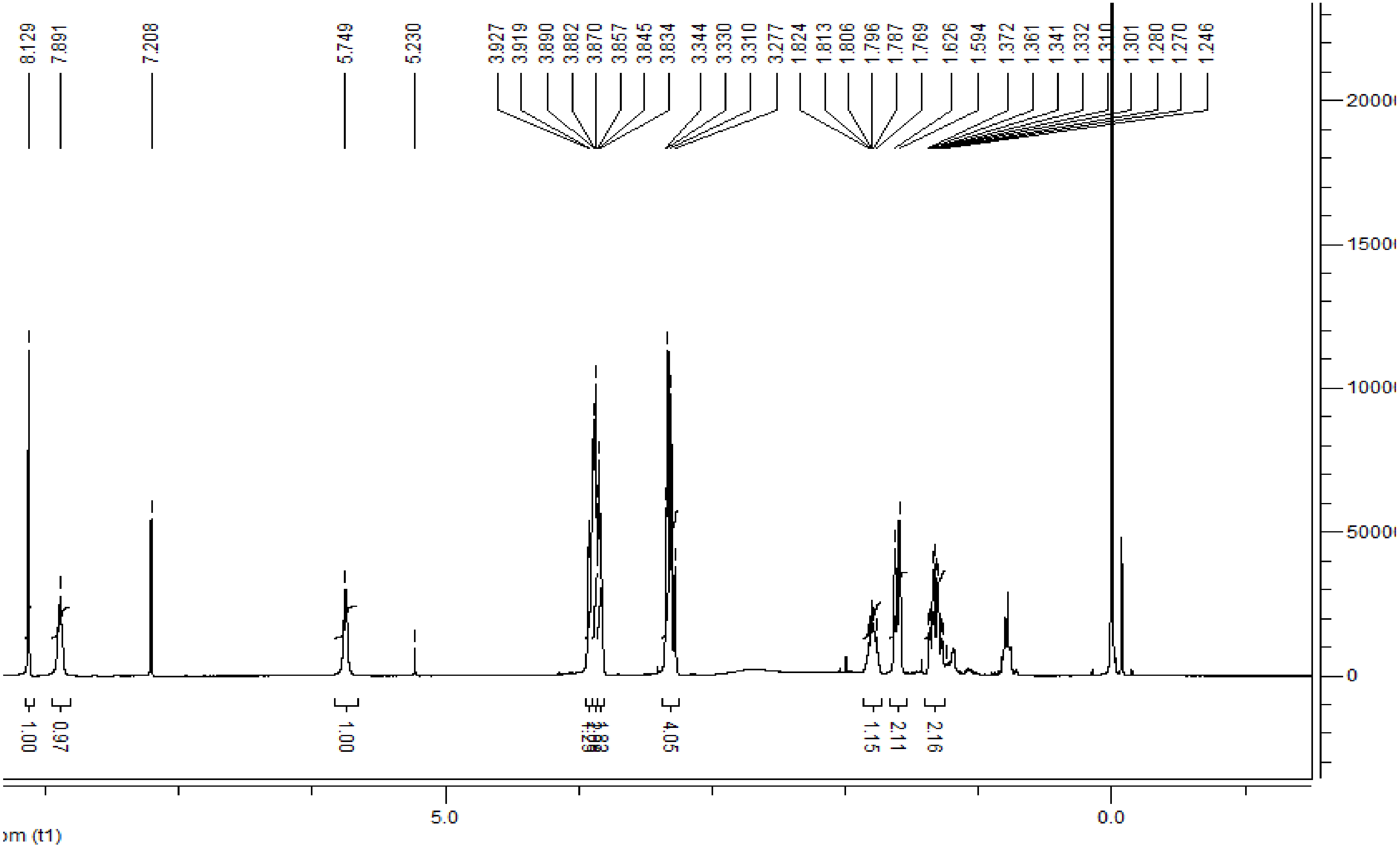
8.129
7.891
7.208
5.749
5.230
3.927
3.919
3.890
3.882
3.870
3.857
3.845
3.834
3.344
3.330
3.310
3.277
1.824
1.813
1.806
1.796
1.787
1.769
1.626
1.594
1.372
1.361
1.341
1.332
1.310
1.301
1.280
1.270
1.246
20000
15000
10000
5000
0
1.00
0.97
1.00
4.05
1.15
2.11
2.16
5.0
0.0
ppm (t1)

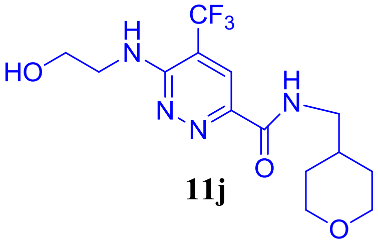

CF3
HO
H
N
N
N
H
N
O
O

11j

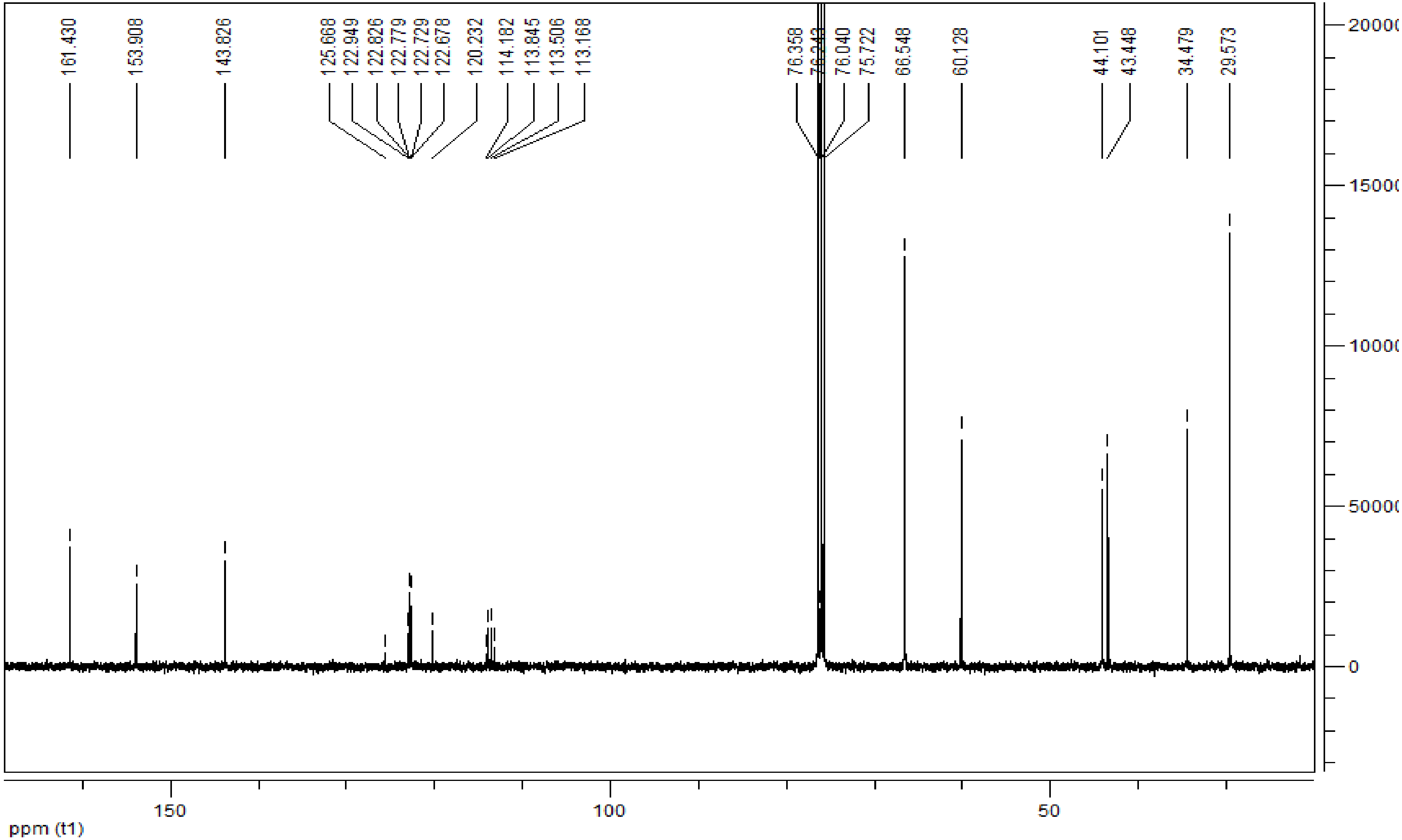

161.430
153.908
143.826
125.668
122.949
122.826
122.779
122.729
122.678
120.232
114.182
113.845
113.506
113.168
76.358
76.040
75.722
66.548
60.128
44.101
43.448
34.479
29.573
200000
150000
100000
50000
0
150
100
50
ppm (t1)

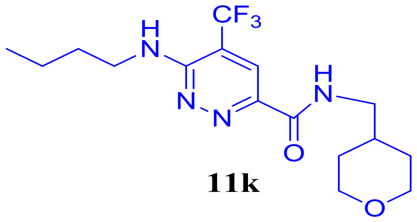

11k

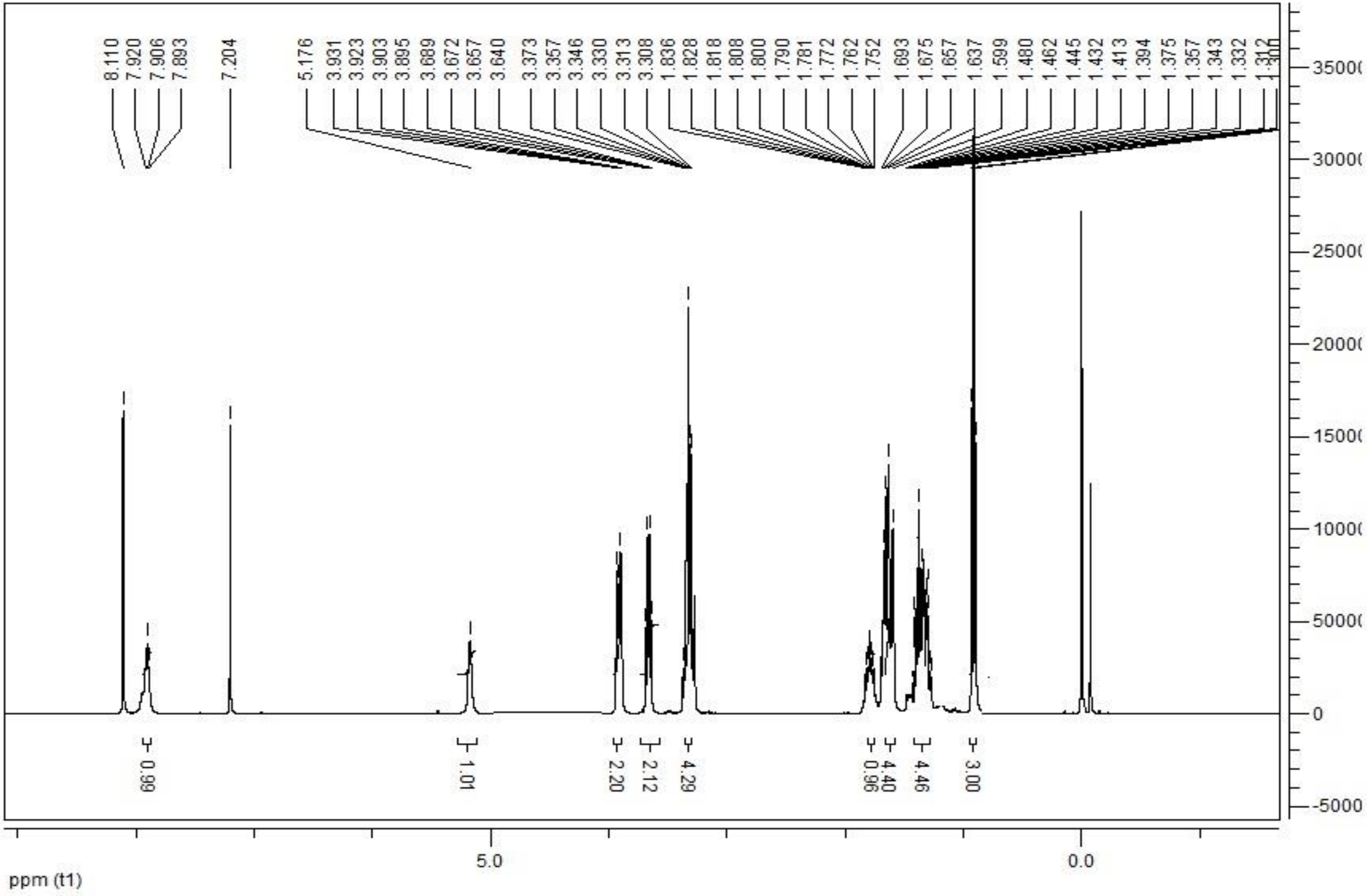

8.110
7.920
7.906
7.893
7.204
5.176
3.931
3.923
3.903
3.895
3.689
3.672
3.657
3.640
3.373
3.357
3.346
3.330
3.313
3.308
1.836
1.828
1.818
1.808
1.800
1.790
1.781
1.772
1.762
1.752
1.693
1.675
1.657
1.637
1.599
1.480
1.462
1.445
1.432
1.413
1.394
1.375
1.357
1.343
1.332
1.312
0.99
1.01
2.20
2.12
4.29
0.96
4.40
4.46
3.00
5.0
0.0
ppm (t1)

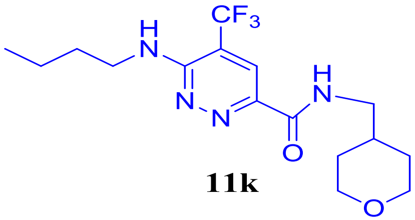
CF3
H
N
N
N
H
N
O
O

11k

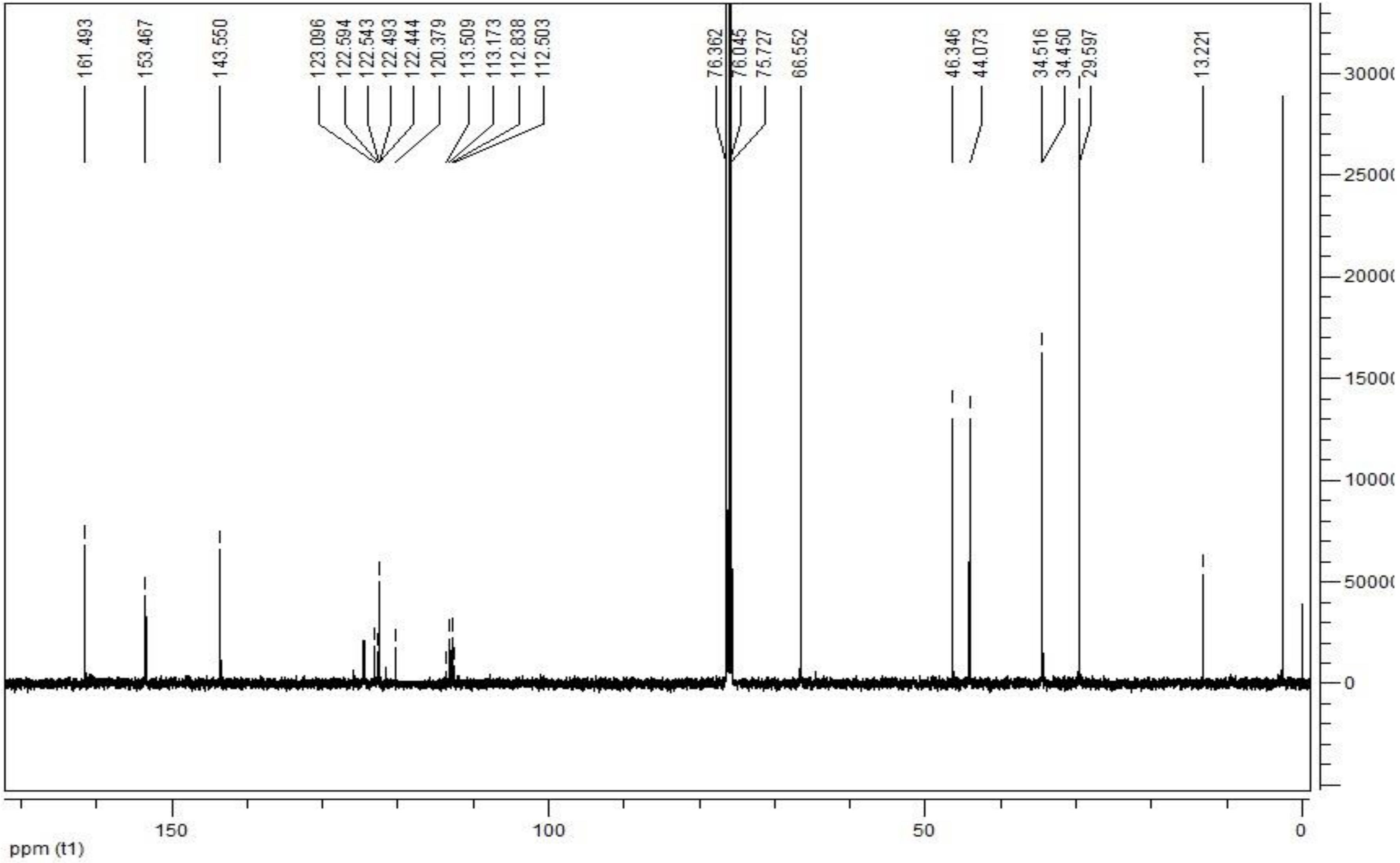
161.493
153.467
143.550
123.096
122.594
122.543
122.493
122.444
120.379
113.509
113.173
112.838
112.503
76.362
76.045
75.727
66.552
46.346
44.073
34.516
34.450
29.597
13.221
150
100
50
0
ppm (t1)

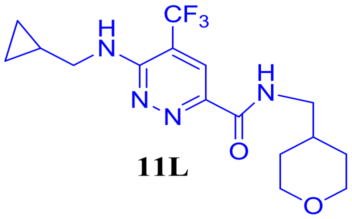

11L

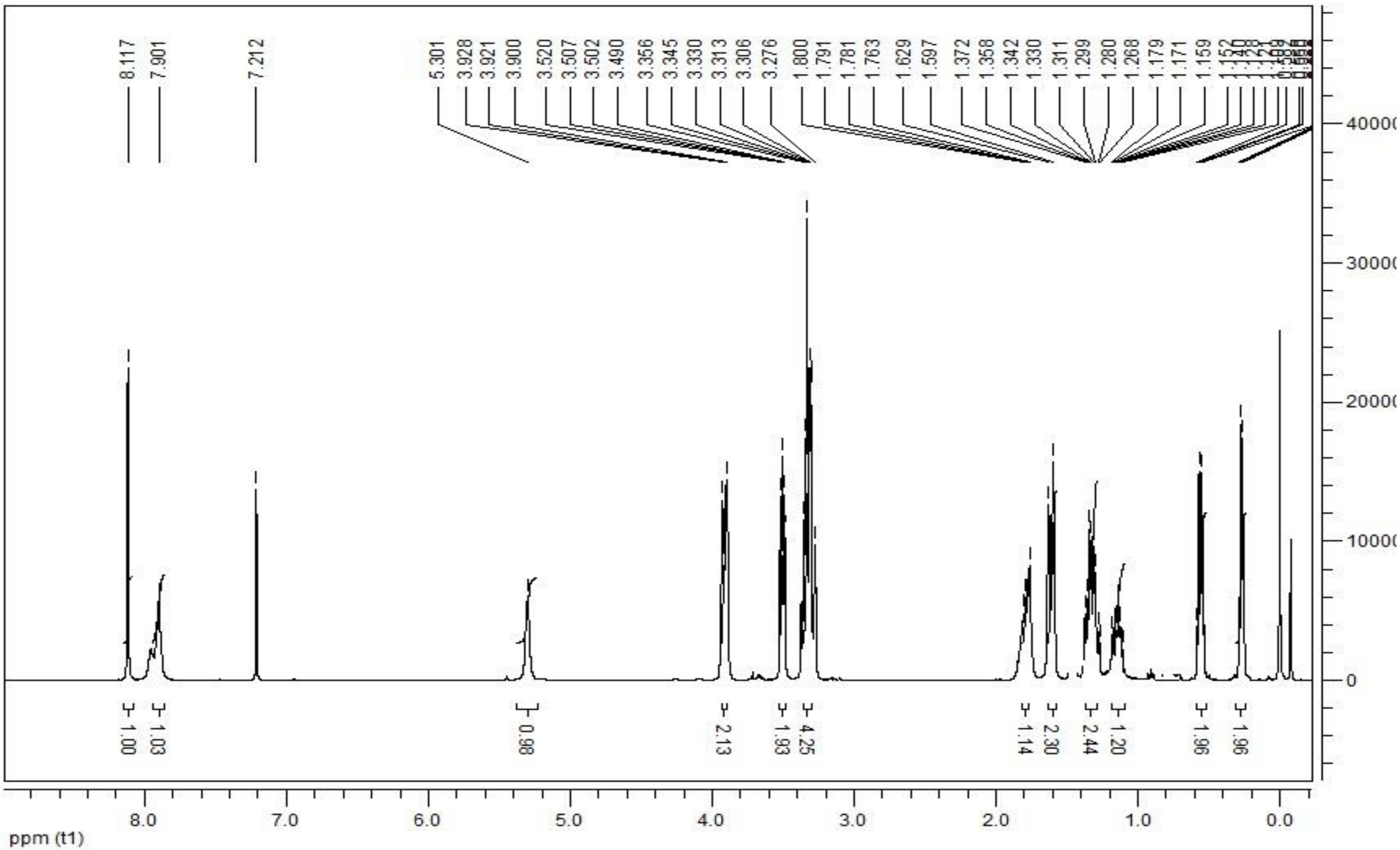

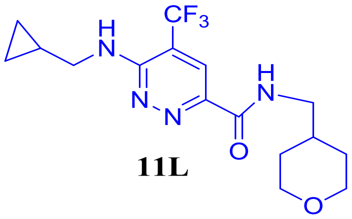
CF3
H
N
N
N
H
N
O
O

11L

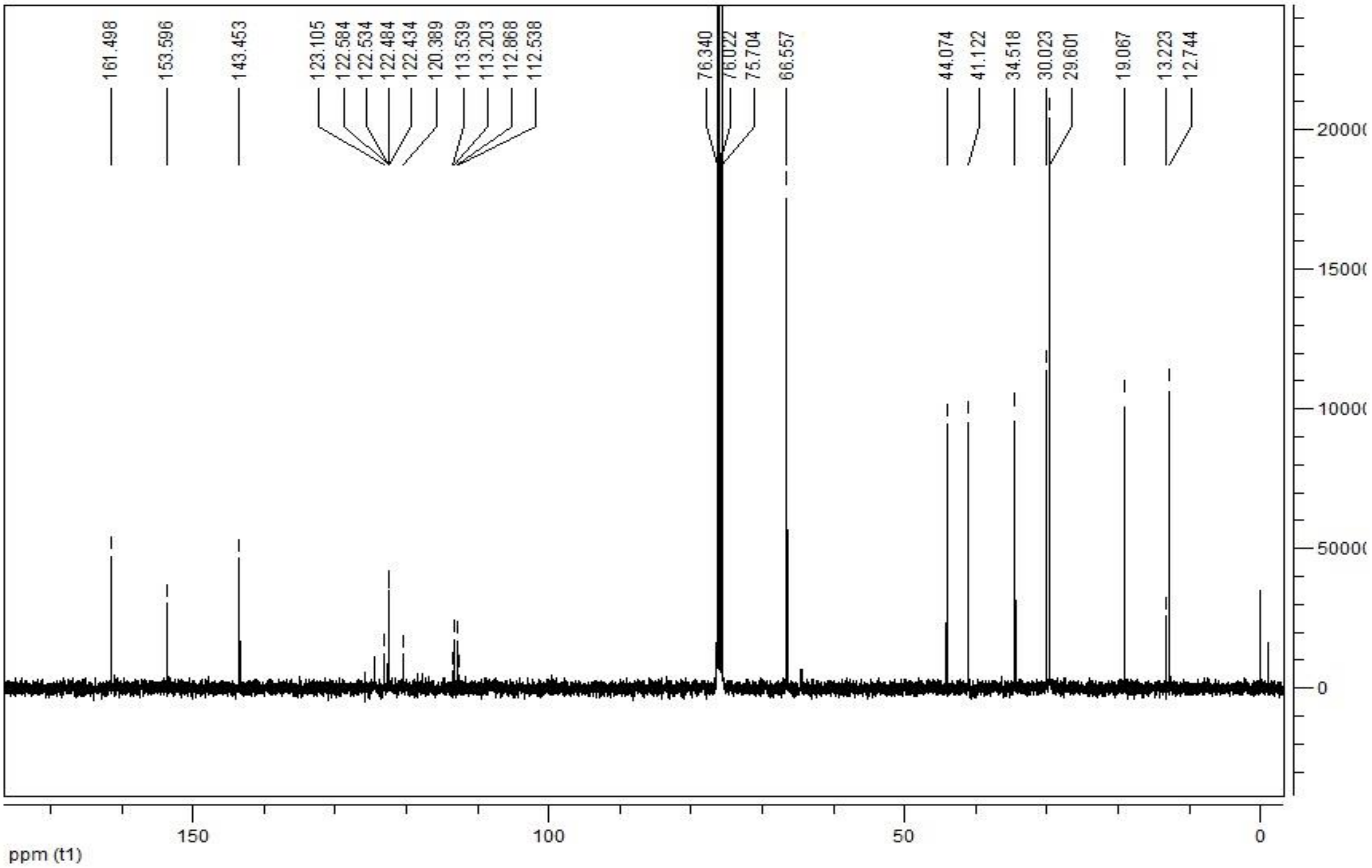
161.498
153.596
143.453
123.105
122.584
122.534
122.484
122.434
120.389
113.539
113.203
112.868
112.538
76.340
76.022
75.704
66.557
44.074
41.122
34.518
30.023
29.601
19.067
13.223
12.744
20000
15000
10000
50000
0
150
100
50
0
ppm (t1)

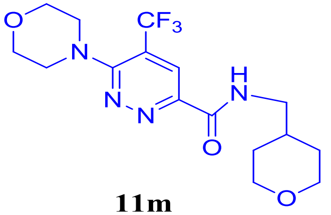

11m

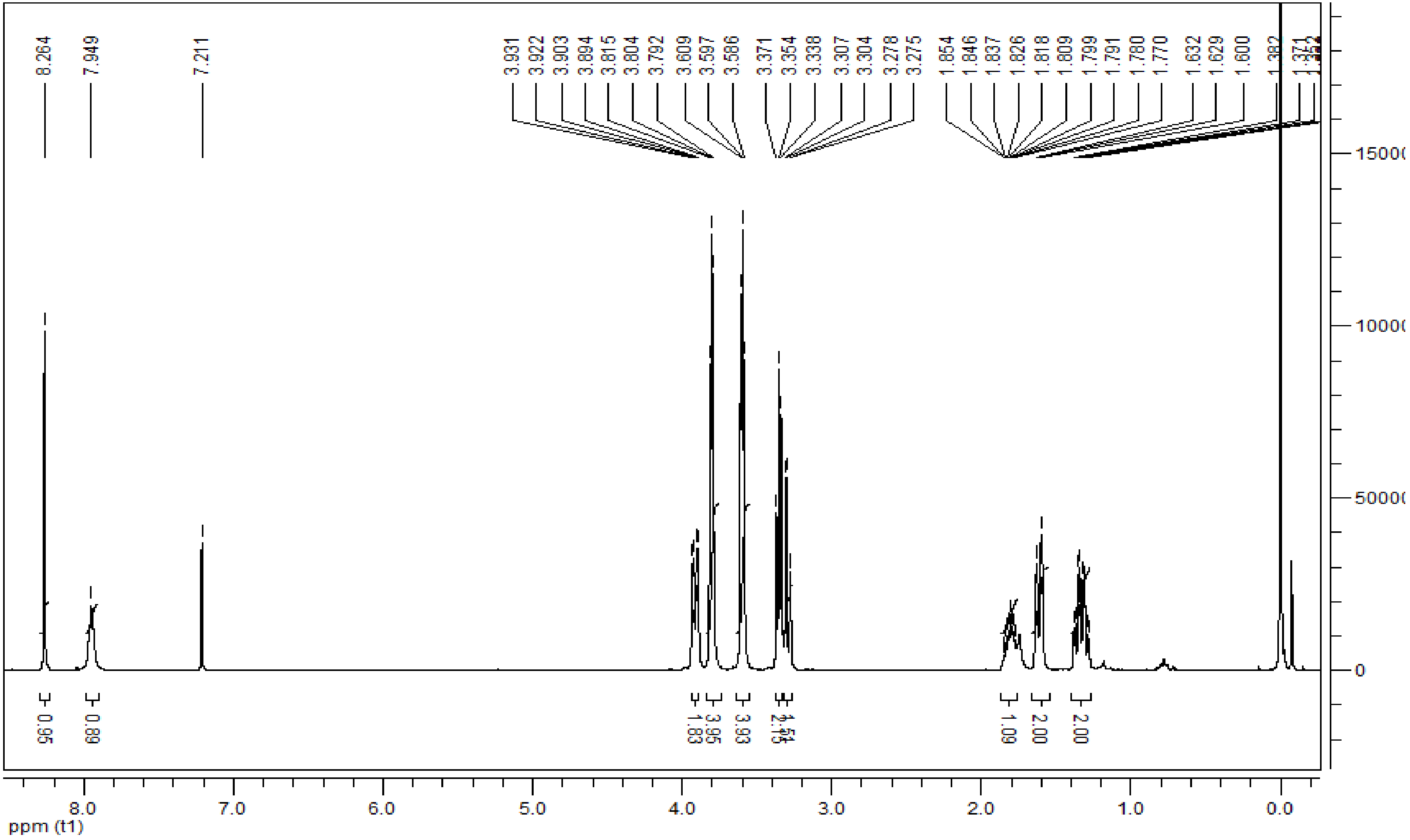

8.264
7.949
7.211
3.931
3.922
3.903
3.894
3.815
3.804
3.792
3.609
3.597
3.586
3.371
3.354
3.338
3.307
3.304
3.278
3.275
1.854
1.846
1.837
1.826
1.818
1.809
1.799
1.791
1.780
1.770
1.632
1.629
1.600
1.382
1.371
150000
100000
50000
0
0.95
0.89
1.83
3.95
3.93
2.15
1.09
2.00
2.00
8.0
7.0
6.0
5.0
4.0
3.0
2.0
1.0
0.0
ppm (t1)

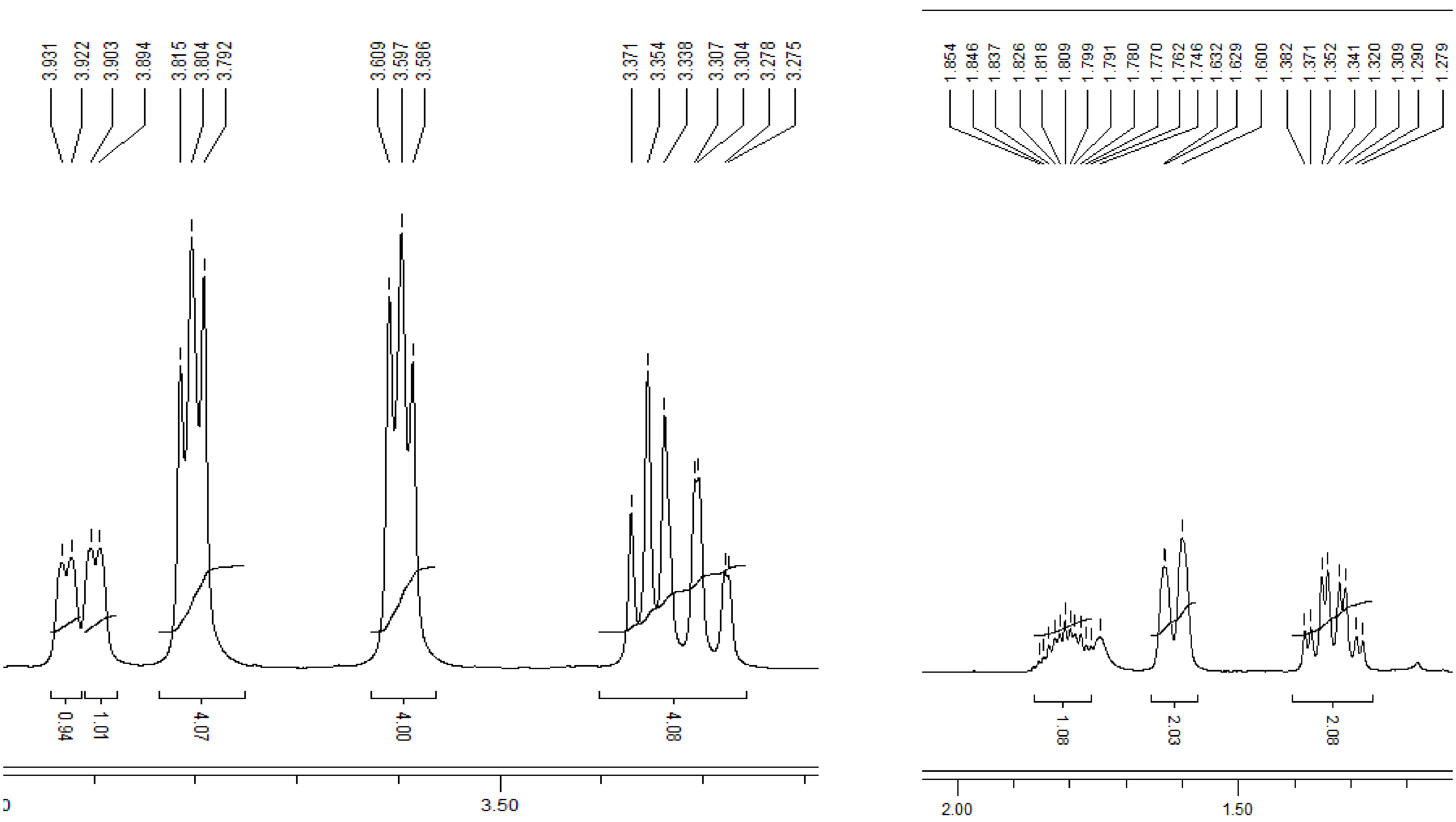

3.931 3.922 3.903 3.894 3.815 3.804 3.792
3.609 3.597 3.586
3.371 3.354 3.338 3.307 3.304 3.278 3.275
1.854 1.846 1.837 1.826 1.818 1.809 1.799 1.791 1.780 1.770 1.762 1.746 1.632 1.629 1.600 1.382 1.371 1.352 1.341 1.320 1.309 1.290 1.279
0.94
1.01
4.07
4.00
4.08
1.08
2.03
2.08
3.50
2.00
1.50

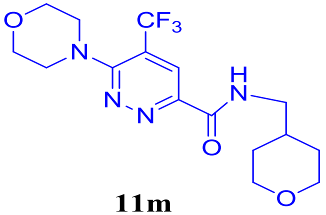

11m

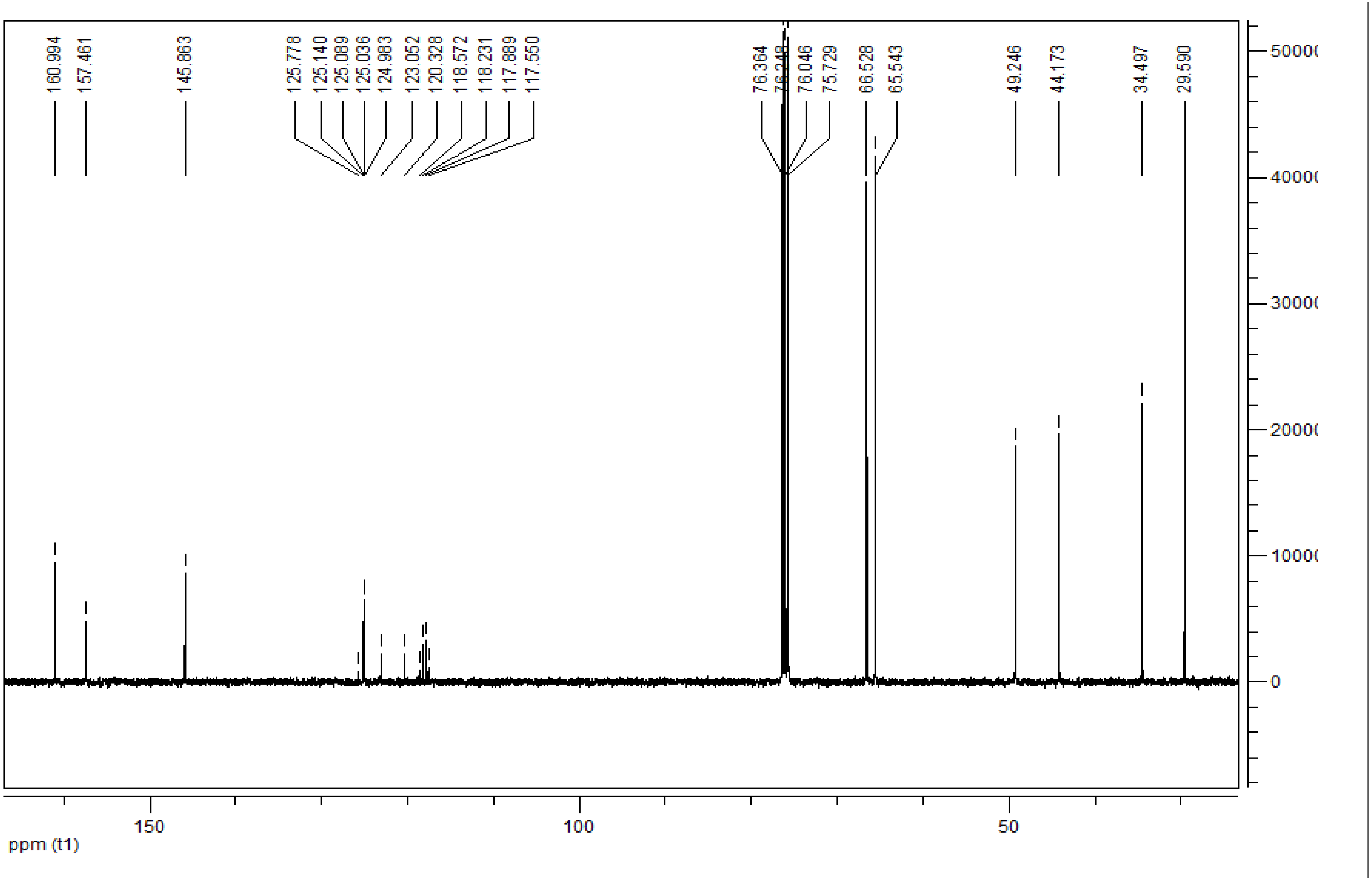
160.994
157.461
145.863
125.778
125.140
125.089
125.036
124.983
123.052
120.328
118.572
118.231
117.889
117.550
76.364
76.046
75.729
66.528
65.543
49.246
44.173
34.497
29.590
150
100
50
ppm (t1)

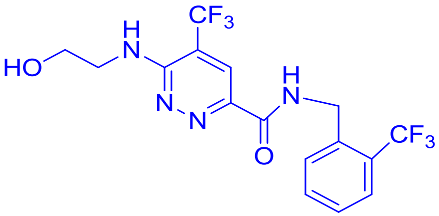

CF3
H
N
HO
N
N
H
N
O
CF3

11n

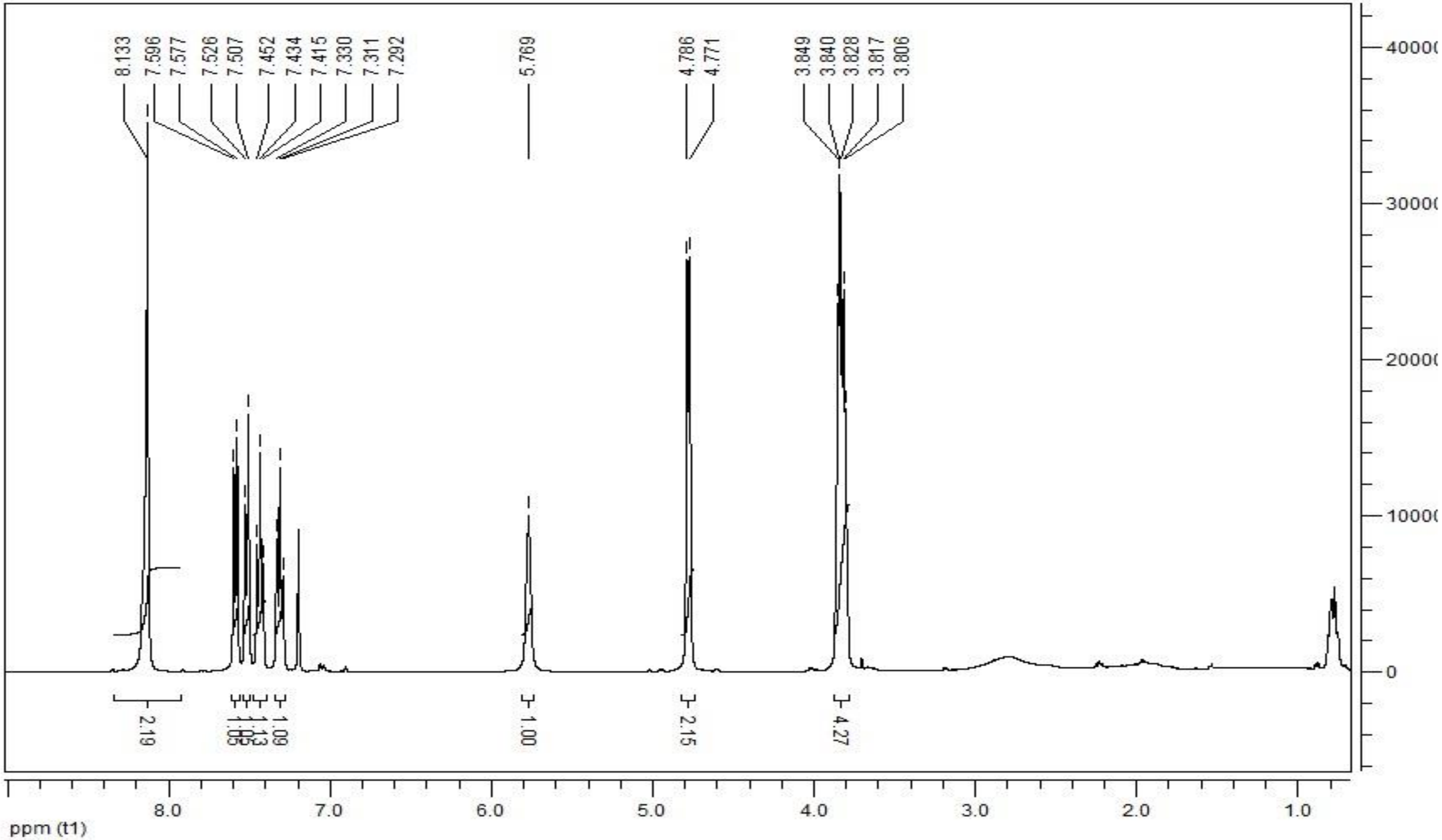

8.133
7.596
7.577
7.526
7.507
7.452
7.434
7.415
7.330
7.311
7.292
5.769
4.786
4.771
3.849
3.840
3.828
3.817
3.806
2.19
1.09
1.00
2.15
4.27
40000
30000
20000
10000
0
8.0
7.0
6.0
5.0
4.0
3.0
2.0
1.0
ppm (t1)

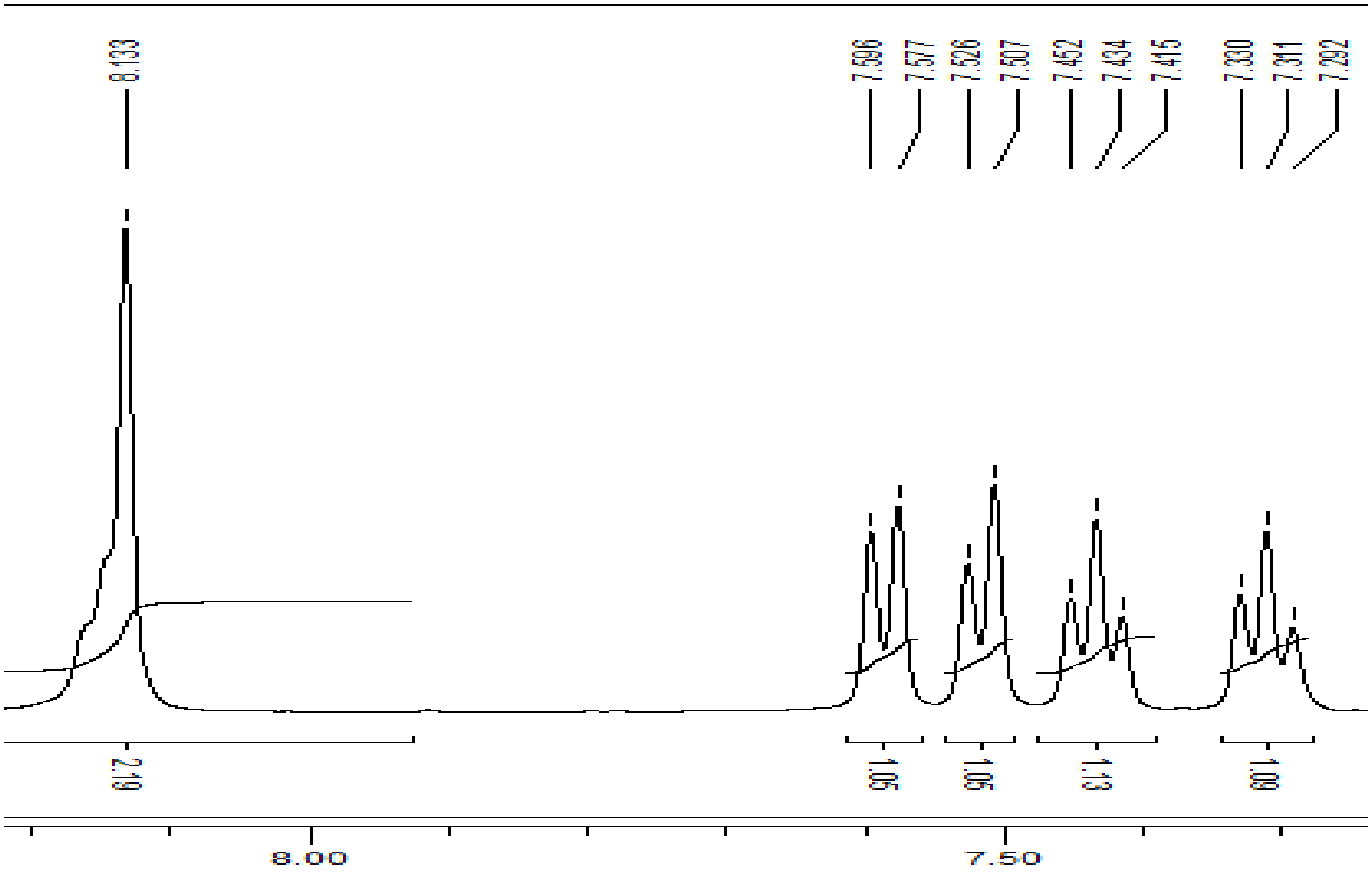

8.133
7.596
7.577
7.526
7.507
7.452
7.434
7.415
7.330
7.311
7.292
2.19
1.05
1.05
1.13
1.09
8.00
7.50

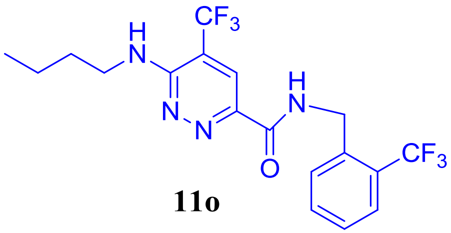

CF3
H
N
N
N
H
N
O
CF3

11o

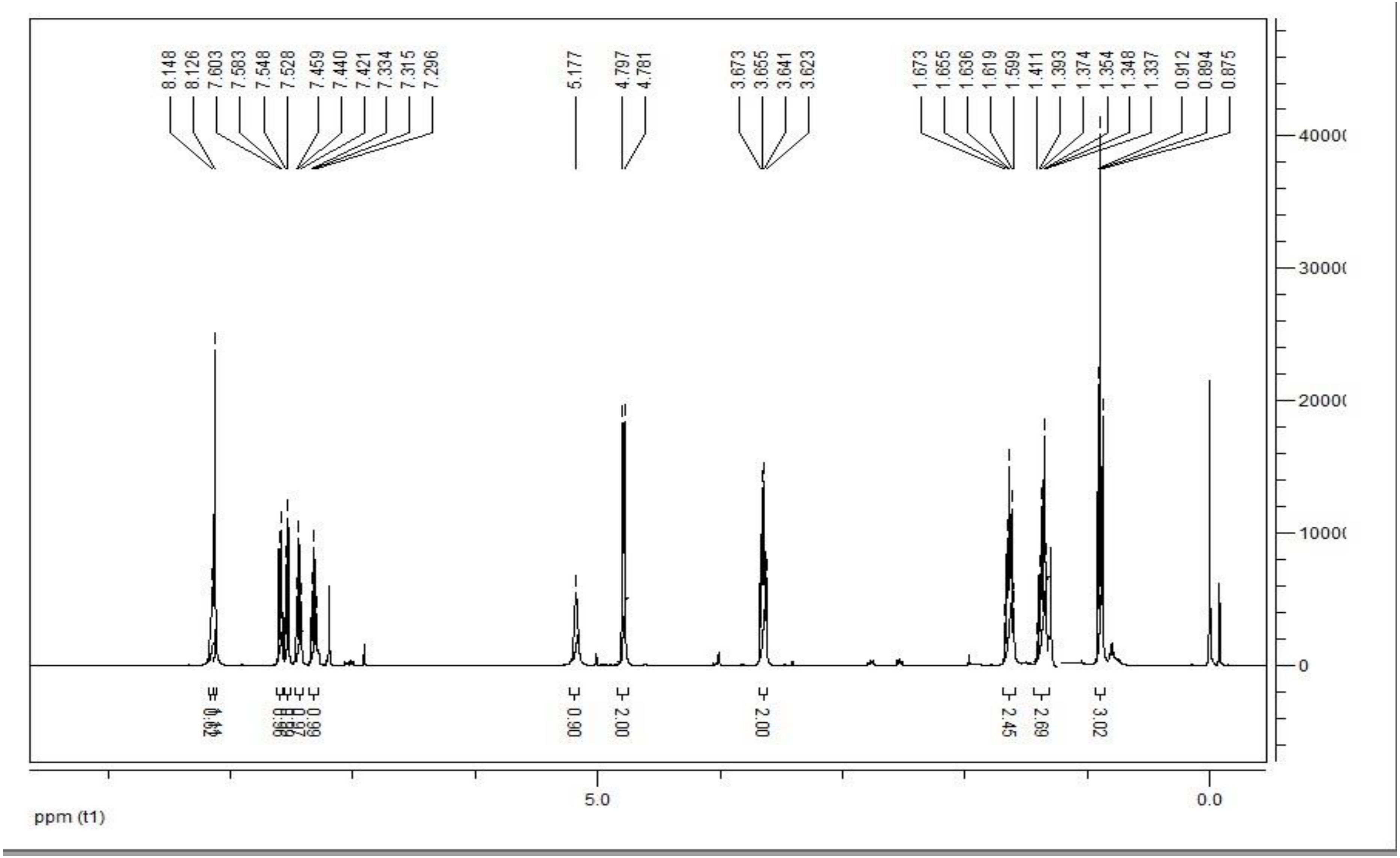

8.148
8.126
7.603
7.583
7.548
7.528
7.459
7.440
7.421
7.334
7.315
7.296
5.177
4.797
4.781
3.673
3.655
3.641
3.623
1.673
1.655
1.636
1.619
1.599
1.411
1.393
1.374
1.354
1.348
1.337
0.912
0.894
0.875
0.99
0.90
2.00
2.00
2.45
2.69
3.02
5.0
0.0
ppm (t1)
0

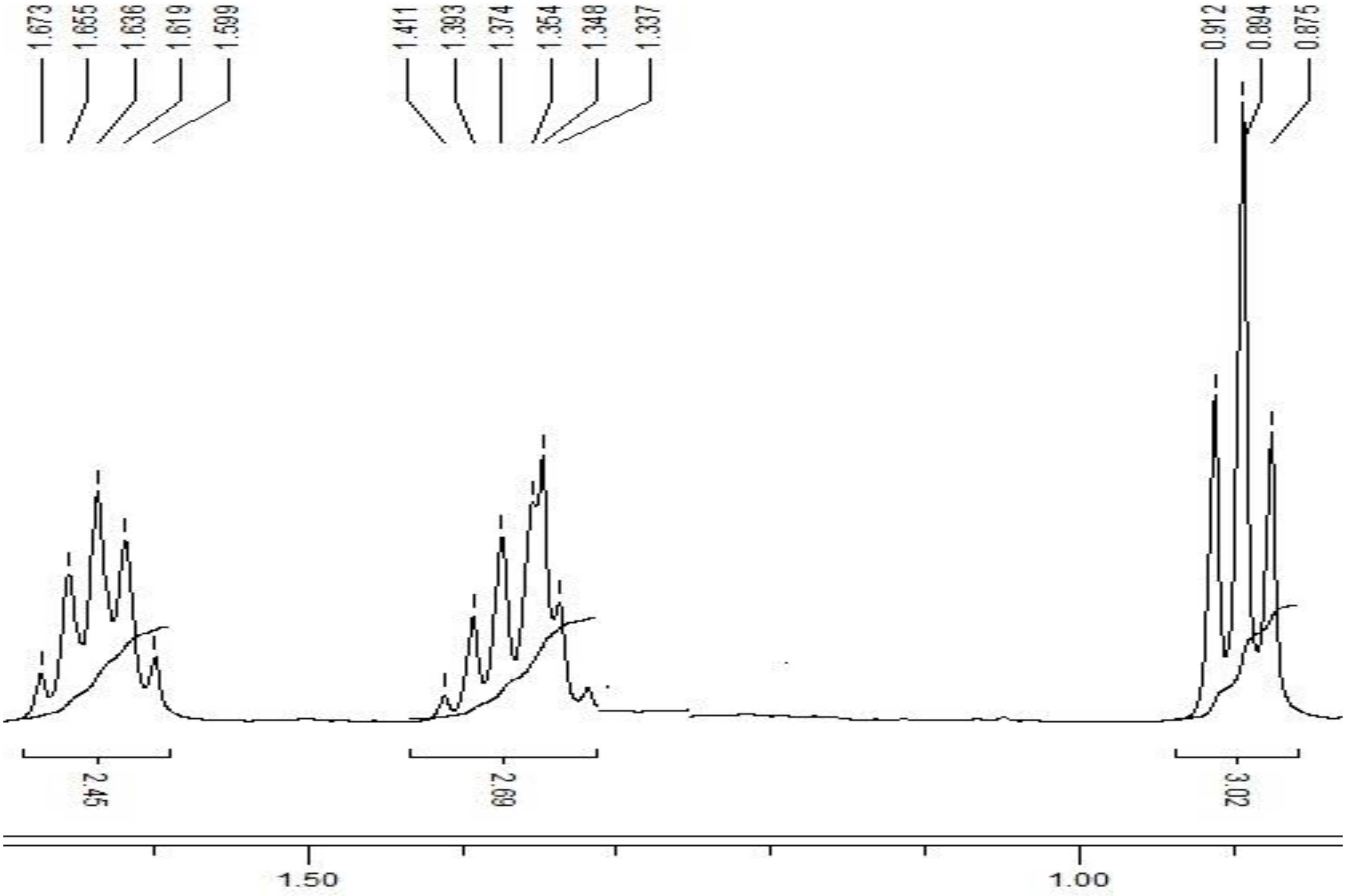
1.673
1.655
1.636
1.619
1.599
1.411
1.393
1.374
1.354
1.348
1.337
0.912
0.894
0.875
2.45
2.69
3.02
1.50
1.00

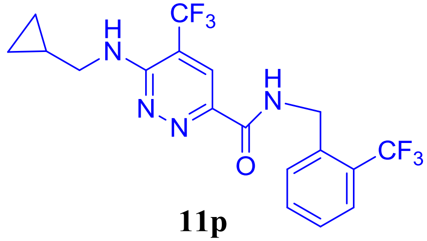

11p

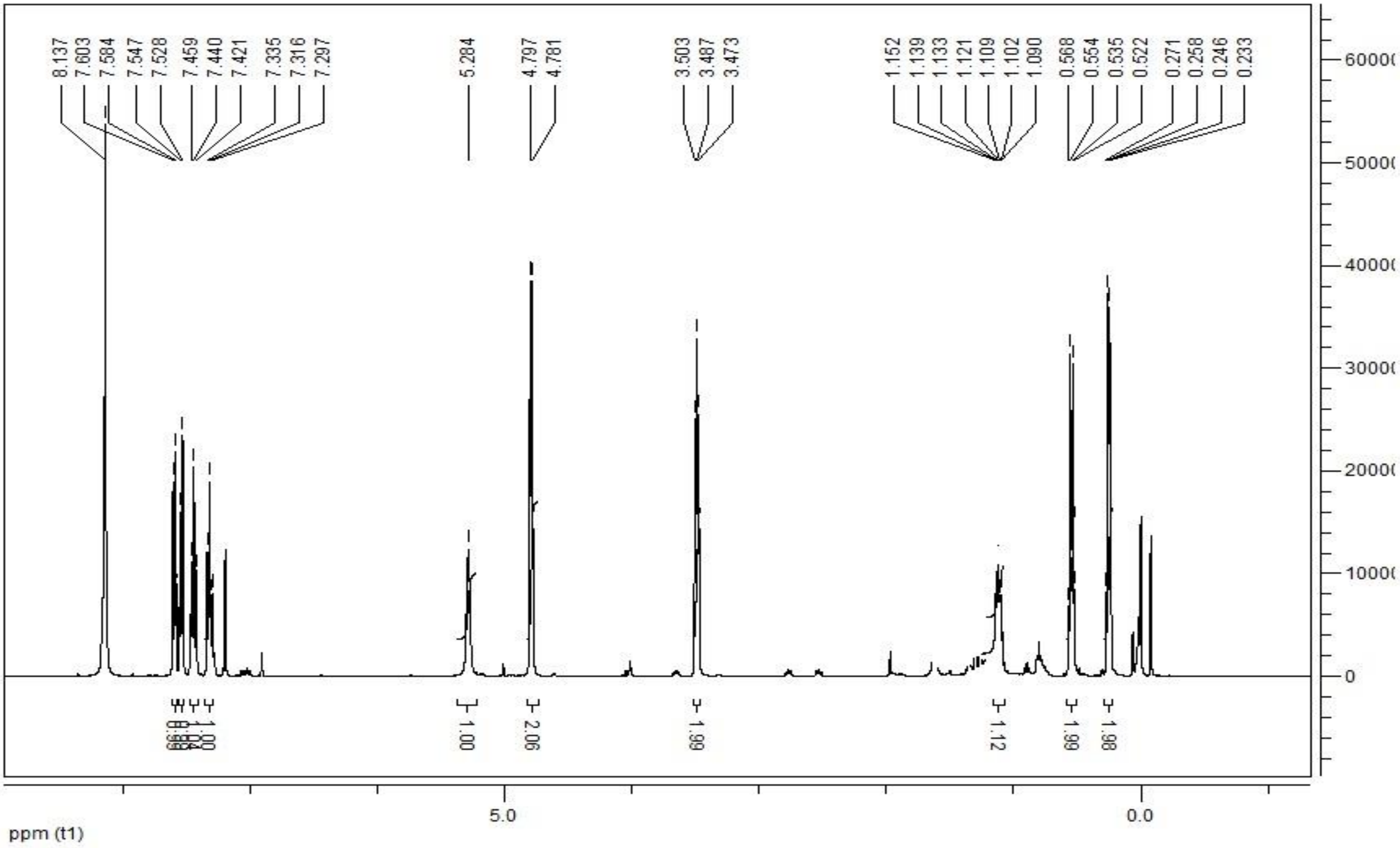

8.137
7.603
7.584
7.547
7.528
7.459
7.440
7.421
7.335
7.316
7.297
5.284
4.797
4.781
3.503
3.487
3.473
1.152
1.139
1.133
1.121
1.109
1.102
1.090
0.568
0.554
0.535
0.522
0.271
0.258
0.246
0.233
1.00
2.06
1.99
1.12
1.99
1.98
5.0
0.0
ppm (t1)

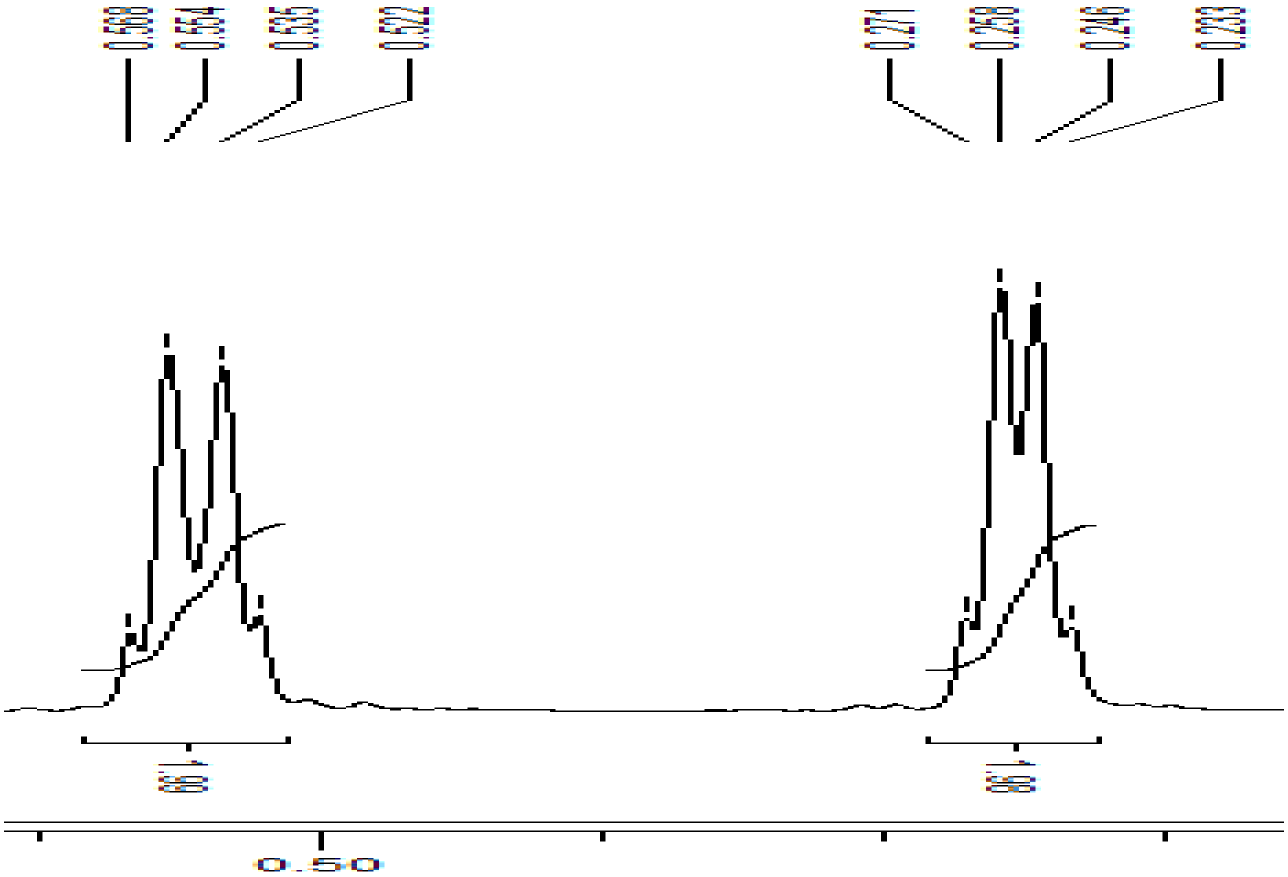

0.568
0.554
0.535
0.522
0.271
0.258
0.246
0.233
0.50

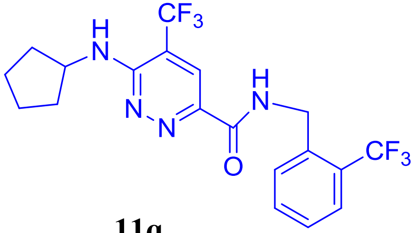

H
N
CF3
N
N
H
N
O
CF3

11q

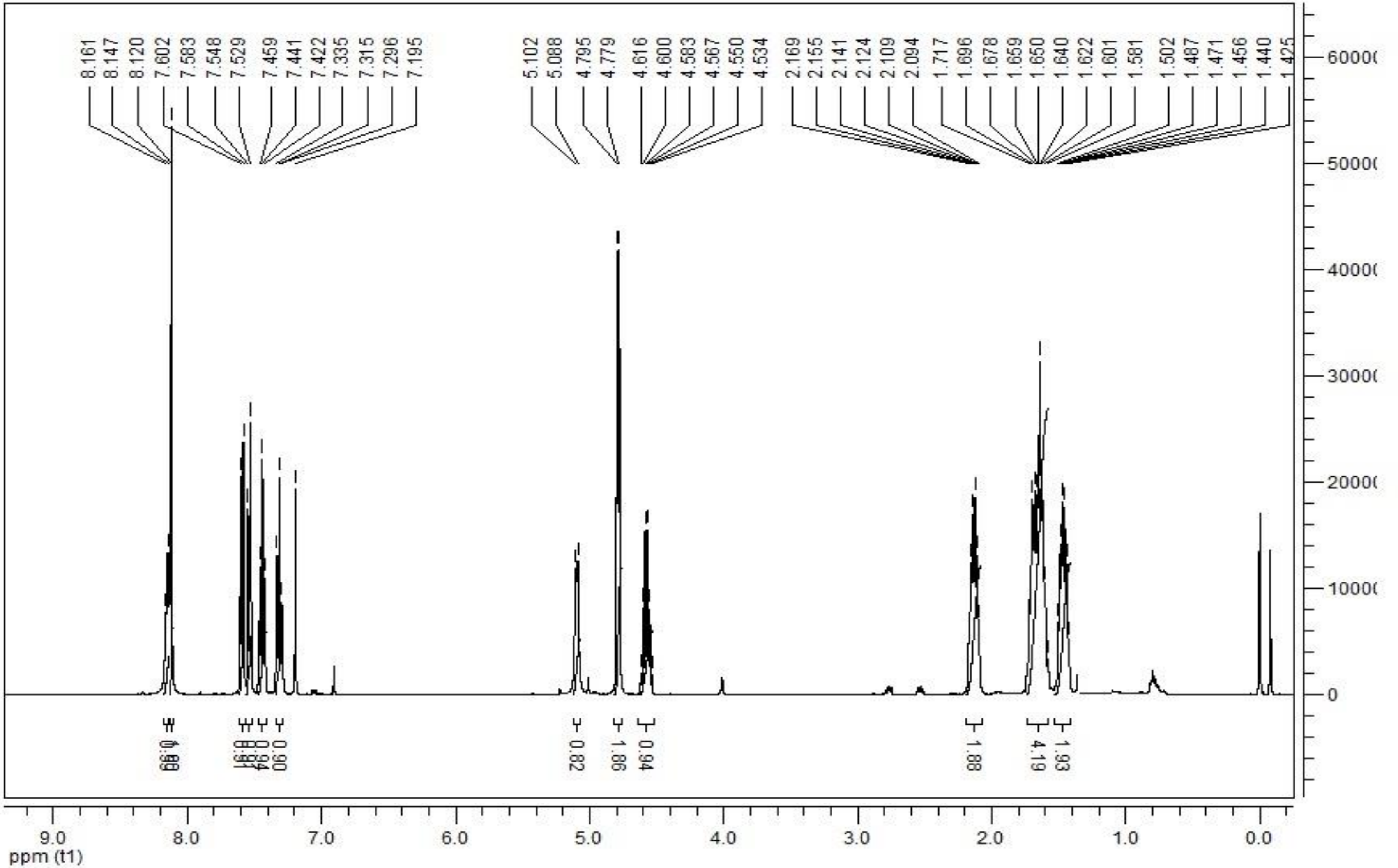

8.161
8.147
8.120
7.602
7.583
7.548
7.529
7.459
7.441
7.422
7.335
7.315
7.296
7.195
5.102
5.088
4.795
4.779
4.616
4.600
4.583
4.567
4.550
4.534
2.169
2.155
2.141
2.124
2.109
2.094
1.717
1.696
1.678
1.659
1.650
1.640
1.622
1.601
1.581
1.502
1.487
1.471
1.456
1.440
1.425
0.90
0.82
1.86
0.94
1.88
4.19
1.93
9.0
8.0
7.0
6.0
5.0
4.0
3.0
2.0
1.0
0.0
ppm (t1)
0

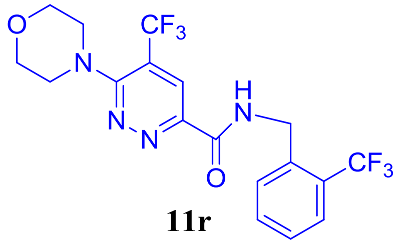

O
N
CF3
N
N
H
N
O
CF3

11r

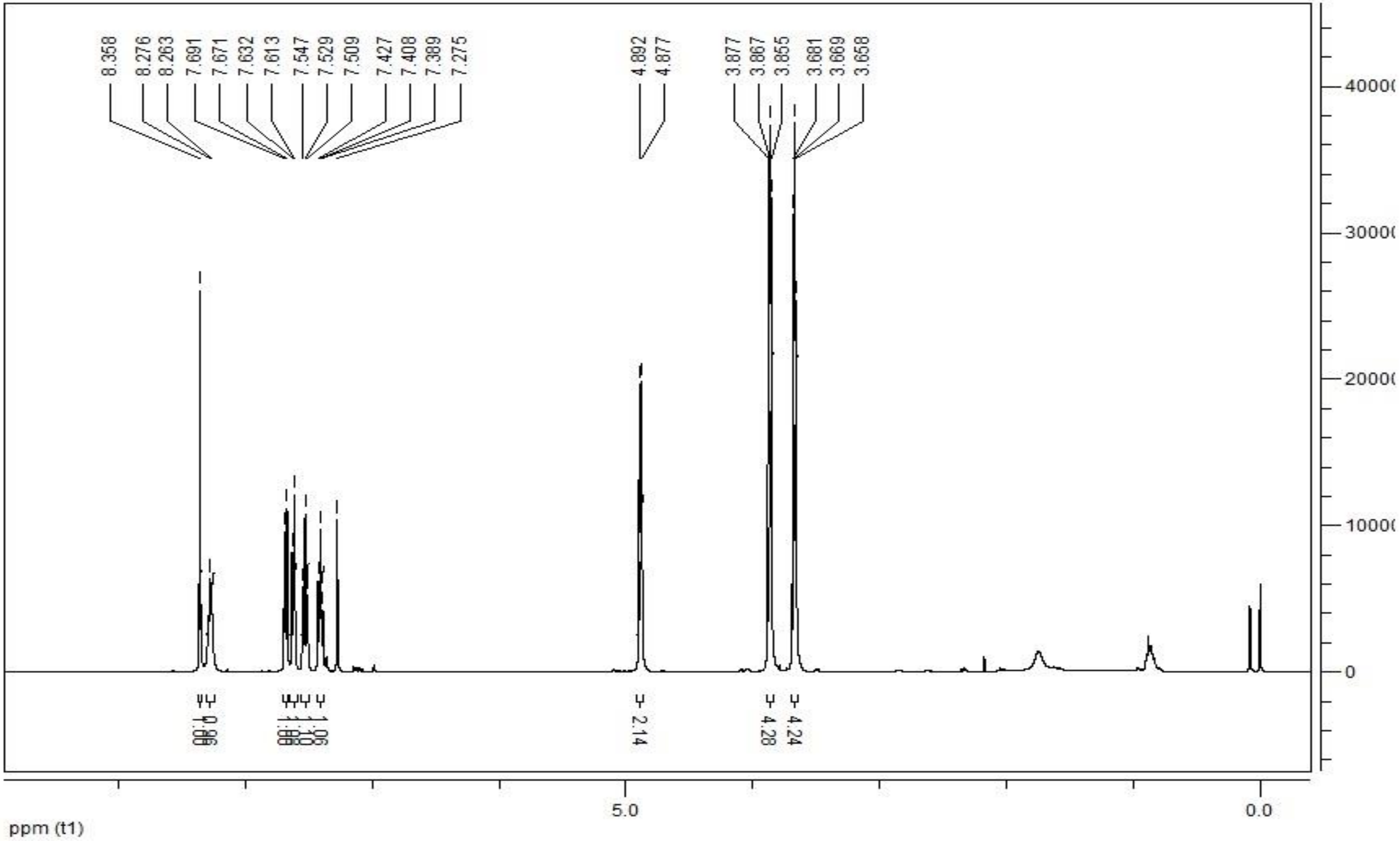

8.358
8.276
8.263
7.691
7.671
7.632
7.613
7.547
7.529
7.509
7.427
7.408
7.389
7.275
4.892
4.877
3.877
3.867
3.855
3.681
3.669
3.658
2.14
4.28
4.24
5.0
0.0
ppm (t1)
0

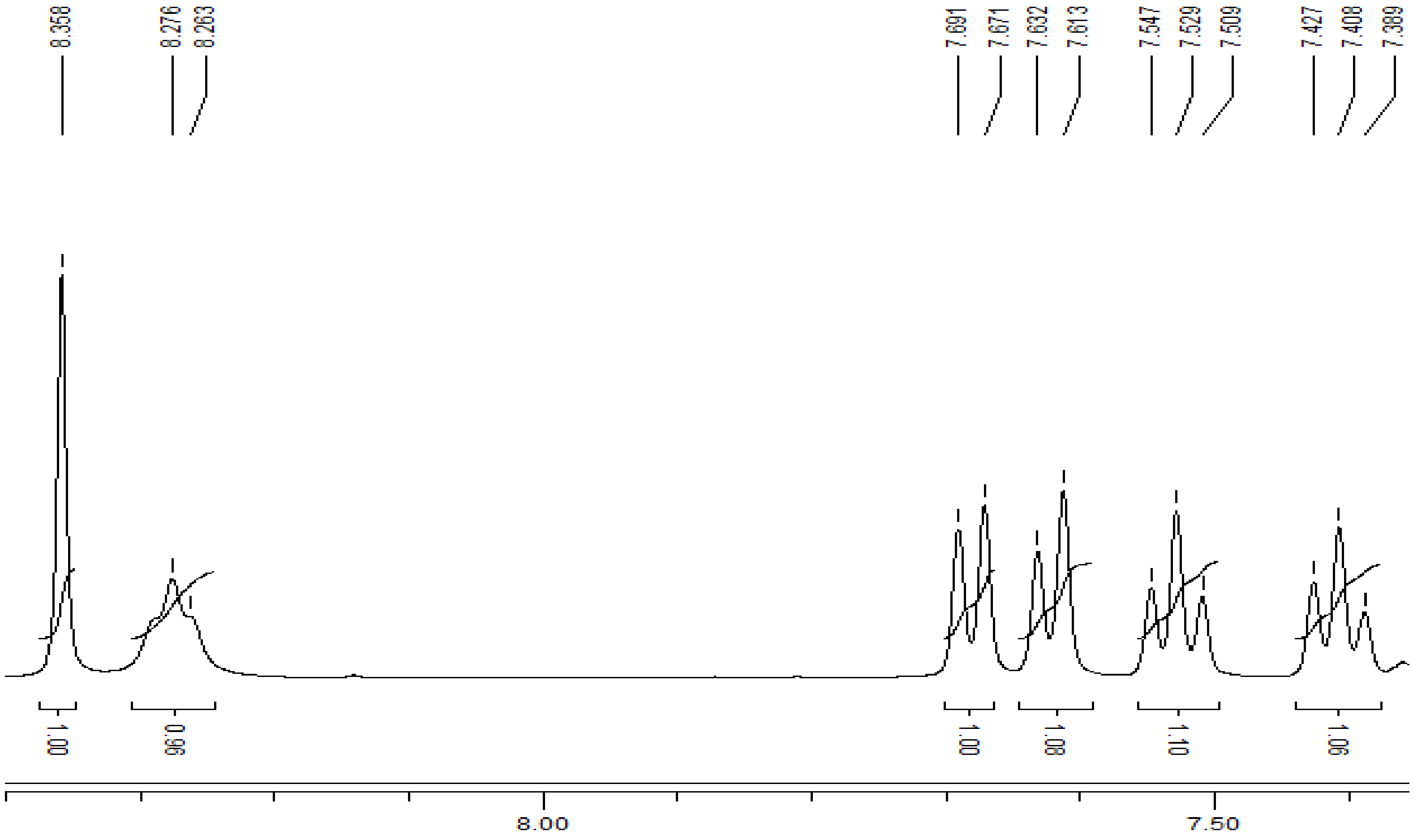

8.358
8.276
8.263
7.691
7.671
7.632
7.613
7.547
7.529
7.509
7.427
7.408
7.389
1.00
0.96
1.00
1.08
1.10
1.06
8.00
7.50

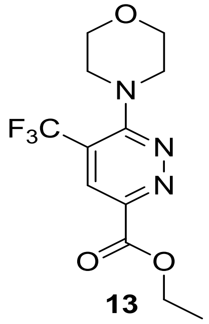

13

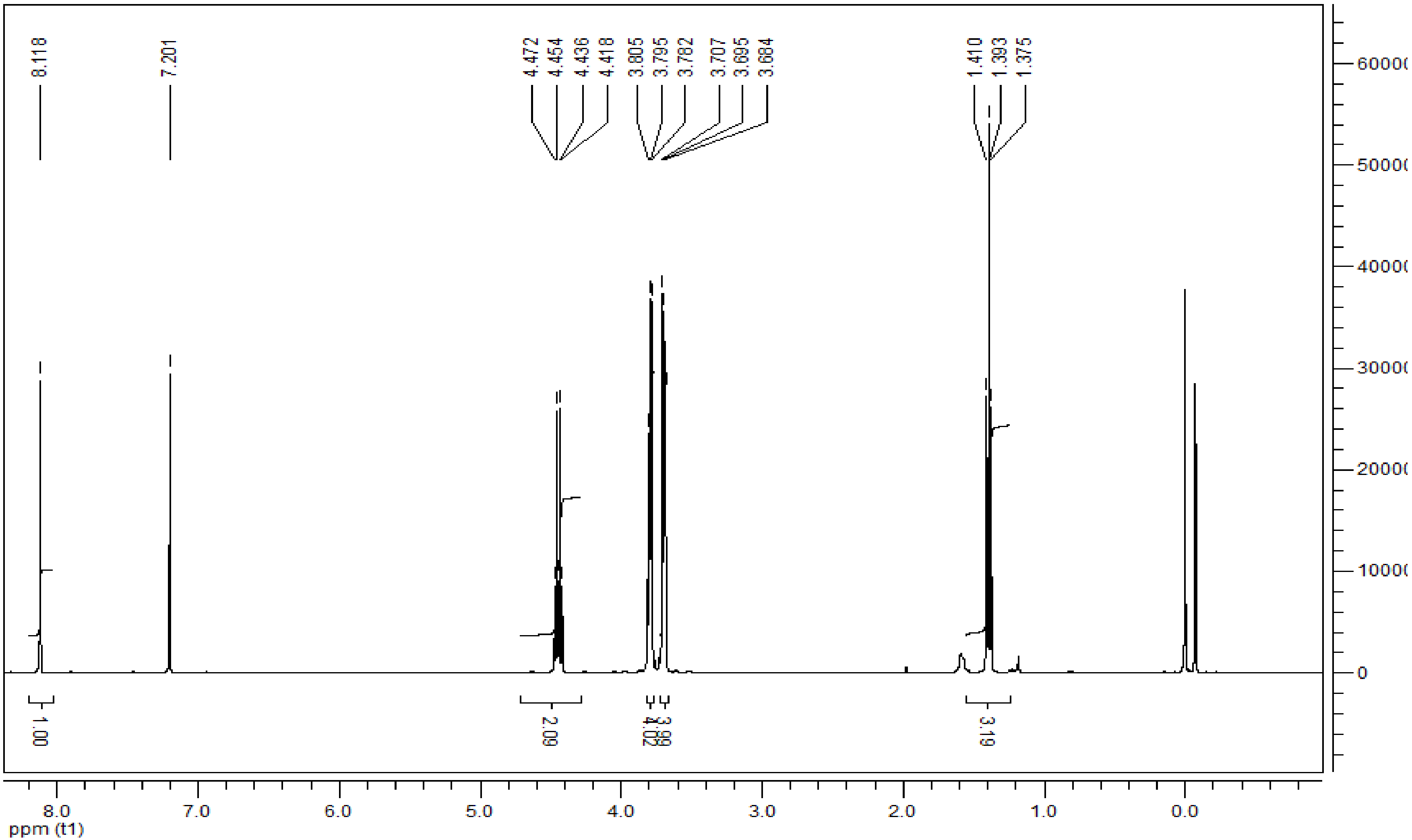

8.118
7.201
4.472
4.454
4.436
4.418
3.805
3.795
3.782
3.707
3.695
3.684
1.410
1.393
1.375
1.00
2.09
4.02
3.99
3.19
60000
50000
40000
30000
20000
10000
0
8.0
7.0
6.0
5.0
4.0
3.0
2.0
1.0
0.0
ppm (t1)
